# ARID1A Is Essential for Endometrial Function during Early Pregnancy

**DOI:** 10.1371/journal.pgen.1005537

**Published:** 2015-09-17

**Authors:** Tae Hoon Kim, Jung-Yoon Yoo, Zhong Wang, John P. Lydon, Shikha Khatri, Shannon M. Hawkins, Richard E. Leach, Asgerally T. Fazleabas, Steven L. Young, Bruce A. Lessey, Bon Jeong Ku, Jae-Wook Jeong

**Affiliations:** 1 Department of Obstetrics, Gynecology and Reproductive Biology, Michigan State University College of Human Medicine, Grand Rapids, Michigan, United States of America; 2 Department of Cardiac Surgery, University of Michigan, Ann Arbor, Michigan, United States of America; 3 Department of Molecular and Cellular Biology, Baylor College of Medicine, Houston, Texas, United States of America; 4 Department of Obstetrics and Gynecology, Baylor College of Medicine, Houston, Texas, United States of America; 5 Department of Women’s Health, Spectrum Health System, Grand Rapids, Michigan, United States of America; 6 Department of Obstetrics and Gynecology, University of North Carolina, Chapel Hill, Chapel Hill, North Carolina, United States of America; 7 Department of Obstetrics and Gynecology, University Medical Group, Greenville Health System, Greenville, South Carolina, United States of America; 8 Department of Internal Medicine, Chungnam National University School of Medicine, Daejeon, South Korea; Vanderbilt University Medical Center, United States of America

## Abstract

AT-rich interactive domain 1A gene (ARID1A) loss is a frequent event in endometriosis-associated ovarian carcinomas. Endometriosis is a disease in which tissue that normally grows inside the uterus grows outside the uterus, and 50% of women with endometriosis are infertile. ARID1A protein levels were significantly lower in the eutopic endometrium of women with endometriosis compared to women without endometriosis. However, an understanding of the physiological effects of *ARID1A* loss remains quite poor, and the function of *Arid1a* in the female reproductive tract has remained elusive. In order to understand the role of *Arid1a* in the uterus, we have generated mice with conditional ablation of *Arid1a* in the *PGR* positive cells (*Pgr* ^*cre/+*^ *Arid1a* ^*f/f*^; *Arid1a* ^*d/d*^). Ovarian function and uterine development of *Arid1a* ^*d/d*^ mice were normal. However, *Arid1a* ^*d/d*^ mice were sterile due to defective embryo implantation and decidualization. The epithelial proliferation was significantly increased in *Arid1a* ^*d/d*^ mice compared to control mice. Enhanced epithelial estrogen activity and reduced epithelial PGR expression, which impedes maturation of the receptive uterus, was observed in *Arid1a* ^*d/d*^ mice at the peri-implantation period. The microarray analysis revealed that ARID1A represses the genes related to cell cycle and DNA replication. We showed that ARID1A positively regulates *Klf15* expression with PGR to inhibit epithelial proliferation at peri-implantation. Our results suggest that *Arid1a* has a critical role in modulating epithelial proliferation which is a critical requisite for fertility. This finding provides a new signaling pathway for steroid hormone regulation in female reproductive biology and furthers our understanding of the molecular mechanisms that underlie dysregulation of hormonal signaling in human reproductive disorders such as endometriosis.

## Author Summary

Endometriosis afflicts about 10% of women of reproductive age and is a major cause of pain and infertility. We showed attenuation of endometrial ARID1A in women with endometriosis as compared to women without endometriosis, and thus hypothesized that ARID1A plays an important role in ensuring normal fertility in the uterus. To test this hypothesis, we generated uterine-specific *Arid1a* knock-out mice, which were infertile due to defective implantation and decidualization. The mutant mice demonstrated increased endometrial epithelial proliferation with enhanced estrogen signaling and attenuation of epithelial PGR. Microarray and ChIP analysis revealed that *Arid1a* suppresses epithelial proliferation with PGR by regulating *Klf15* expression. These data suggest that *Arid1a* plays an important role in steroid hormone signaling in endometrial function and dysfunction. Further investigation of ARID1A will be important for understanding altered endometrial function in infertility and endometriosis and in developing therapies for these disorders.

## Introduction

Endometriosis is one of the most significant diseases affecting females of reproductive-age and affects an estimated 5 million women in the United States. Endometriosis is defined as the presence of endometrium-like tissue outside of the uterine cavity. The incidence increases up to 50% in patients with infertility and up to 45% in patients with chronic pelvic pain [1,2]. Infertility and pregnancy loss are major public health concerns for reproductive-age women. Establishment of uterine receptivity by the sequential actions of estrogen (E2) and progesterone (P4) on uterine cells is critical for successful embryo apposition, attachment, implantation, and pregnancy maintenance. Lack of sufficient E2 and P4 action can result in infertility and pregnancy loss in humans [3,4] and mice [5]. One of the primary effects of E2 on the endometrium is stimulation of epithelial proliferation, while the primary effects of P4 are to inhibit epithelial proliferation and induce differentiation to an embryo receptive state [6,7]. Cellular E2 and P4 actions can occur directly on a specific cell type and indirectly via paracrine activity mediated by another cell type. P4 through its cognate receptor, the progesterone receptor (PGR), have important roles in the establishment and maintenance of pregnancy [7–10]. P4 attenuates E2 stimulated epithelial cell proliferation by epithelial PGR [11].

At the time of embryo implantation, the expression of PGR is promptly downregulated in the luminal epithelium in both humans and mice, and its expression is increased in stromal cells, anticipating the role of PGR in induction of decidualization [12]. Epithelial PGR acts to inhibit E2-induced epithelial proliferation. Epithelial PGR female mice are infertile due to embryo implantation defects indicating that epithelial PGR is essential for uterine function [11].

Endometriosis regression was found in some patients with endometriosis during pregnancy or who were exposed to progestin-based therapeutics [13,14]. However there are endometriosis patients who do not respond to treatment due to progesterone resistance. The molecular changes by P4 in the eutopic endometrium from women with endometriosis are either blunted or undetectable. P4 cannot inhibit E2-dependent growth of endometriosis[15]. The previous microarray studies of comparing women with and without endometriosis reported that many of the P4 target genes were altered at the time of implantation when P4 levels are highest [16,17]. P4 therapy also prevents the development of endometrial cancer associated with unopposed E2 by blocking E2 actions [18]. Expression of PGR was known as positively correlated with a good prognosis and responsiveness to progestin treatment [19]. However, more than 30% of patients with progestin treatment did not respond to progestin due to de novo or acquired progestin resistance [20–24]. The mechanism of progestin resistance is still unknown. Understanding the molecular mechanisms regulating E2 and P4 actions in the endometrium is critical in developing therapeutic approaches to alleviate this women’s health crisis.

Ovarian clear-cell and endometrioid carcinomas are associated with endometriosis through distinct but currently unknown mechanisms [25–27]. One of the possible mechanisms is linked to mutation of the AT-rich interactive domain 1A gene (*ARID1A*) [28]. *ARID1A* encodes BAF250a (ARID1A) protein which is one of the subunits in the switch/sucrose non-fermentable (SWI/SNF) chromatin remodeling complex [29]. *ARID1A* mutations leading to loss of the protein expression [30] have been found in 46% of ovarian clear-cell carcinomas and 30% of endometrioid ovarian carcinomas [28,31]. ARID1A is also critical for embryogenesis in mice and the maintenance of ES cell self-renewal, as well as lineage-specific differentiation of ES cells in vitro [32]. Embryos lacking one allele resulted in late embryonic lethality, complete loss of ARID1A led to developmental arrest around E6.5 without formation of a primitive streak and mesoderm. Ablation of ARID1A in mice ES cells led to altered cell morphology and proliferation [33]. However, little is known about the physiological or pathological effects of ARID1A expression in the endometrium.

We found that ARID1A levels are remarkably lower in endometrium from women with endometriosis compared to women without endometriosis. In an effort to overcome embryonic lethality of *Arid1a* knock-out mice, we have used conditional *Arid1a* knock-out mice in the uterus. In this study, we observed that the mutant mice are sterile due to increased epithelial cell proliferation which resulted in implantation defects. Our results suggest that *Arid1a* suppresses E2 signaling with PGR by modulating KLF15 expression indicating the critical role of *Arid1a* in the peri-implantation period.

## Results

### Attenuation of ARID1A in eutopic endometrial tissue from women with endometriosis

**Fig 1:**
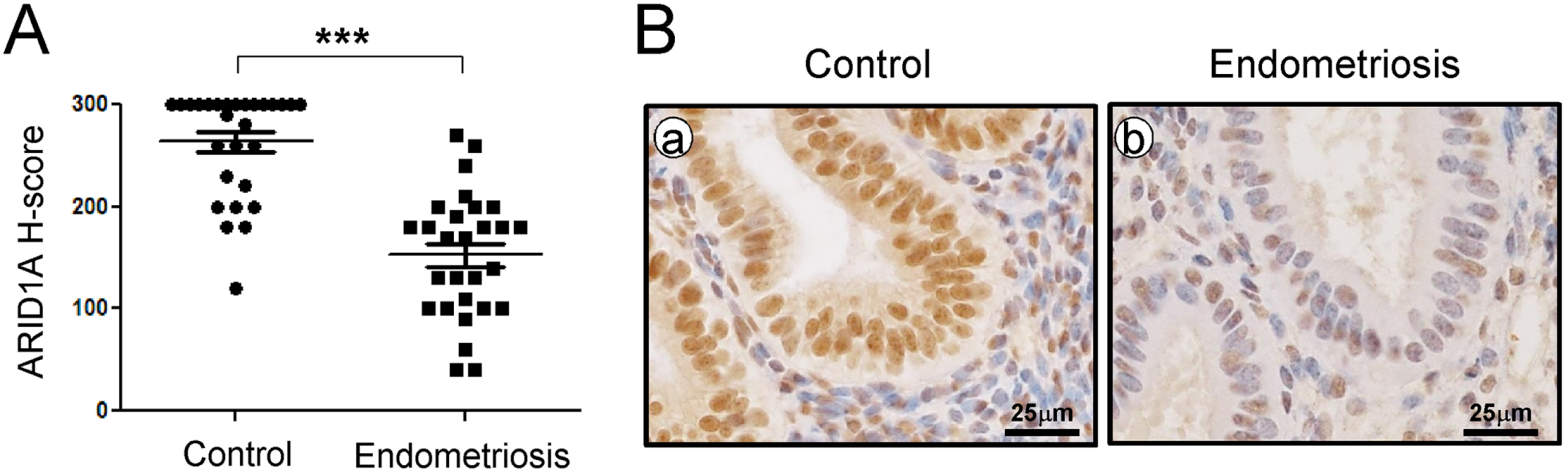
ARID1A loss in eutopic endometrial tissue from infertile women with endometriosis. (A) The immunohistochemical histological score (H-score) of ARID1A proteins. The results represent the mean ± SEM. *** *p*<0.001. (B) Representative photomicrograph of immunohistochemical staining of ARID1A proteins in human endometrium with and without endometriosis.

We examined the levels of ARID1A in endometrium from spontaneously cycling women using immunohistochemical analysis. We observed the most abundant levels of ARID1A protein throughout the menstrual cycle in women without endometriosis (S1 Fig). ARID1A proteins were strongly detected in the stromal and epithelial cells of endometrium from the proliferative phase and early, mid, and late secretory phases in women without endometriosis (n = 7 per stage). However, the levels of ARID1A were significantly lower in both the stromal and epithelial cells of endometrium from proliferative and secretory phase endometriosis patients (n = 28) compared to women without endometriosis (n = 28) (Fig 1).

To determine whether ARID1A is expressed during pregnancy, we next examined the mRNA and protein levels of ARID1A in the uteri of wild-type mice during early pregnancy by real-time RT-PCR and immunohistochemical analysis (S2 Fig). The initiation of pregnancy was marked by the presence of the postcoital vaginal plug (0.5 dpc). The expression of *Arid1a* mRNA was strongly detected on 0.5 dpc, which consistently expressed until 6.5 dpc in the uterus. To further investigate the spatiotemporal expression profiles of ARID1A protein in the uterus during early pregnancy, we performed immunohistochemistry analysis during sequential time points. Consistent with the real-time PCR results, ARID1A proteins were also consistently strong in the nucleus of epithelial and stromal cells during early pregnancy. These data suggest that ARID1A may play an important role during early pregnancy.

### Fertility defect of mice with ablation of *Arid1a* in the PGR-expressing cells

*Arid1a* knock-out mice resulted in embryo lethality [33]. Therefore, in order to investigate the role of *Arid1a* in the uterus, we generated a mouse model in which *Arid1a* gene expression is ablated specifically in the PGR-expressing cells (*Pgr* ^*cre/+*^ *Arid1a* ^*f/f*^; *Arid1a* ^*d/d*^). ARID1A proteins were remarkably reduced in *Arid1a* ^*d/d*^ mice by western blot (S3A Fig). The uteri of *Arid1a* ^*f/f*^ control mice showed abundant ARID1A proteins at the luminal epithelium, glandular epithelium and stroma, whereas this staining was absent in the *Arid1a* ^*d/d*^ mice (S3B Fig). These results confirm our successful ablation of *Arid1a* within the uterus of *Arid1a* ^*d/d*^ mice.

To investigate the impact of ablation of *Arid1a* on female fertility, female control (*Arid1a* ^*f/f*^) and *Arid1a* ^*d/d*^ mice were mated with wild-type male mice for 6 months. *Arid1a* ^*f/f*^ mice (n = 9) had an average of 7.21± 0.29 pups/litter, whereas *Arid1a* ^*d/d*^ mice (n = 9) had no pups (S1 Table). These results revealed that *Arid1a* ^*d/d*^ mice were sterile. To test for an ovarian phenotype, female *Arid1a* ^*d/d*^ mice were examined for their ability to ovulate normally in response to a superovulatory regimen of gonadotropins [34]. *Arid1a* ^*d/d*^ mice yielded 19.86 ± 0.99 oocytes which did not differ significantly from *Arid1a* ^*f/f*^ mice (19.50 ± 1.85) (S2 Table). Also, histological analysis of the *Arid1a* ^*d/d*^ ovary did not show any alterations in ovarian morphology *Arid1a* ^*d/d*^ mice showed normal development of corpora lutea (n = 5) (S4A Fig). The serum level of E2 and P4 were 4.40± 0.71 pg/ml and 11.57± 1.88 ng/ml, respectively in *Arid1a* ^*f/f*^ mice, meanwhile 5.43± 0.50 pg/ml and 15.69± 1.96 ng/ml, respectively in *Arid1a* ^*d/d*^ mice. The serum level of E2 and P4 showed no significant statistical difference between the mice at 3.5 dpc (n = 3 per genotype) (S4B Fig). This result shows that ovarian morphology and functioning were not affected in the *Arid1a* ^*d/d*^ females suggesting that the fertility defect is primarily due to a uterine defect.

### Implantation defect in *Arid1a* ^*d/d*^ mice

**Fig 2:**
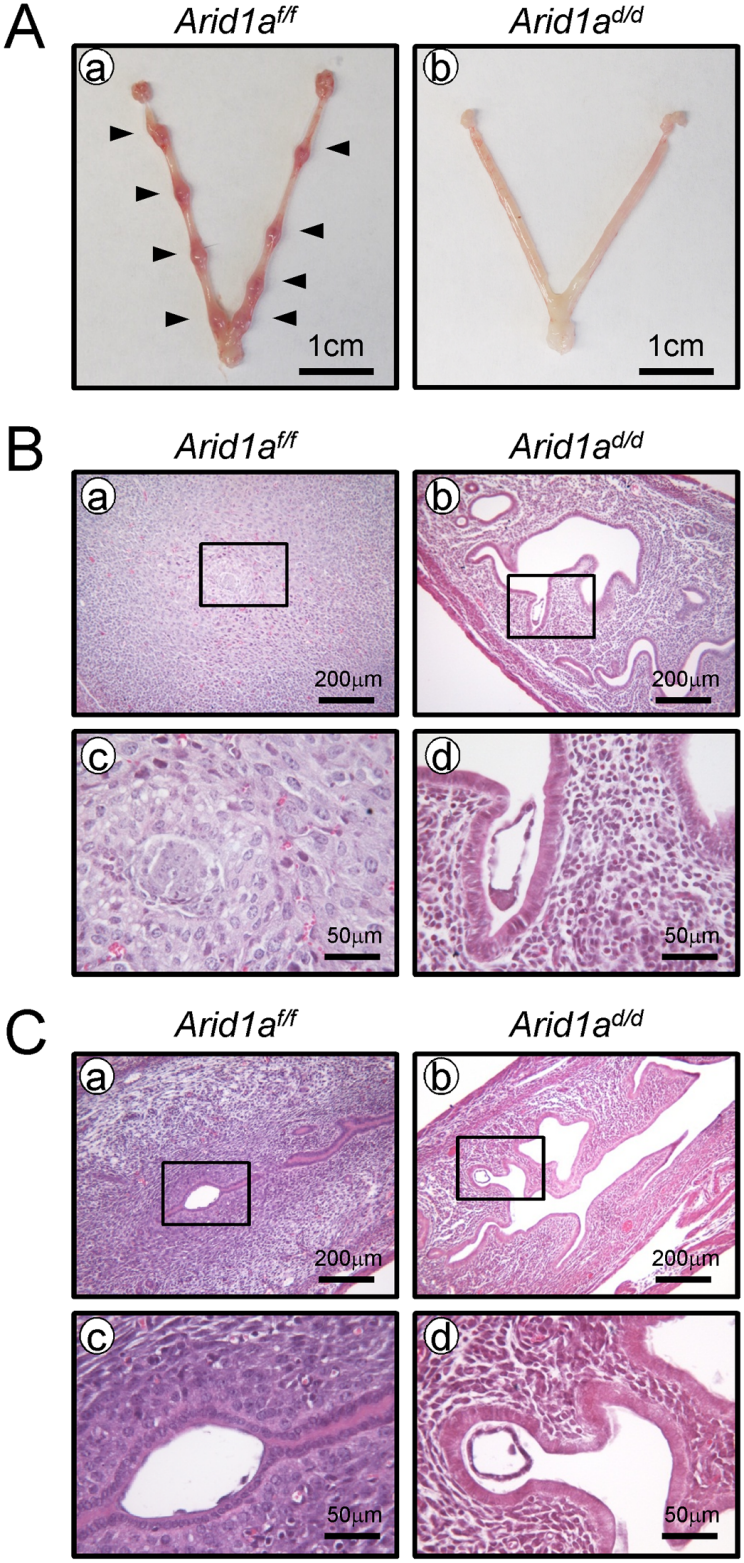
A failure of implantation in *Arid1a* ^*d/d*^ mice. (A) Implantation sites were not detected in the uteri of *Arid1a* ^*d/d*^ mice (n = 5), compared with *Arid1a* ^*f/f*^ mice at 5.5 dpc (n = 5). Arrow heads indicate implantation sites. (B) Histology of implantation site in *Arid1a* ^*f/f*^ (a and c) and *Arid1a* ^*d/d*^ mice (b and d) at 5.5 dpc. (C) While well-attached embryos were found in *Arid1a* ^*f/f*^ (a and c), free-floating embryos were found in the uterine cavity of *Arid1a* ^*d/d*^ mice at 4.5 dpc (b and d).

To determine the cause of infertility in *Arid1a* ^*d/d*^ mice, 8-week-old female *Arid1a* ^*f/f*^ and *Arid1a* ^*d/d*^ mice were mated with intact wild-type male mice. Females were euthanized at 5.5 dpc of pregnancy, and the numbers of implantation sites were counted. Implantation sites were detected in the uterine horn of *Arid1a* ^*f/f*^ mice, whereas there were no implantation sites in *Arid1a* ^*d/d*^ mice (Fig 2A). Histological analysis revealed that embryos could not attach to the uterine horn of *Arid1a* ^*d/d*^ mice while embryos were attached well in *Arid1a* ^*f/f*^ mice and surrounded by decidualized cells (n = 5) (Fig 2B). To address a defect of embryo implantation in *Arid1a* ^*d/d*^ mice, mice were dissected at 4.5 dpc. Free-floating embryos (4.67 ± 1.33 per mouse) were found in the uterine horn of *Arid1a* ^*d/d*^, whereas well attached embryos (5.50 ± 0.65 per mouse) were found in the uterine horn of *Arid1a* ^*f/f*^ mice (n = 5) (Fig 2C). These results suggest that a failure of embryo attachment is one of the causes of the infertility observed in *Arid1a* ^*d/d*^ mice.

### Decidualization defect in *Arid1a* ^*d/d*^ mice

**Fig 3:**
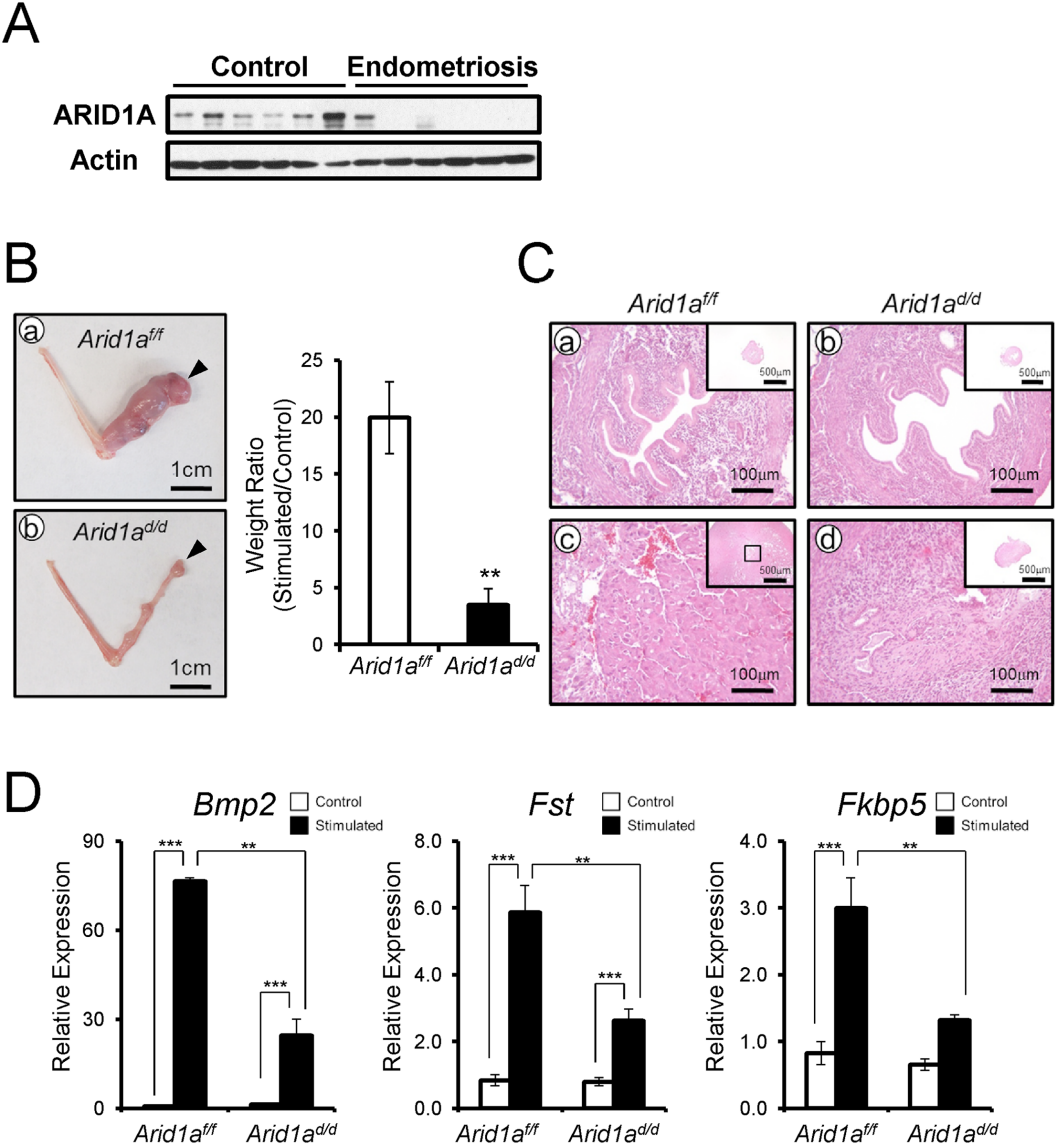
*Arid1a* ^*d/d*^ mice exhibited an altered decidualization. (A) The ARID1A expression were lower in infertile women with endometriosis (n = 6) compared to women without endometriosis (n = 6). (B) The decidualization is highly induced in *Arid1a* ^*f/f*^ mice (a) but not *Arid1a* ^*d/d*^ mice. Arrow heads indicate stimulated horns. (b). The uterine weight ratio was significantly decreased in *Arid1a* ^*d/d*^ mice as compared to *Arid1a* ^*f/f*^ (C) Histology of control and stimulated horn in *Arid1a* ^*f/f*^ (a and c) and *Arid1a* ^*d/d*^ mice (b and d) at day 5, respectively (D) The expression of decidualization marker genes, *Bmp2*, *Fst* and *Fkbp5* was measured in the uteri of control and stimulated horn. The results represent the mean ± SEM of three independent RNA sets. **, *p* < 0.01; ***, *p* < 0.001.

Embryo invasion transforms endometrial stromal cells into a decidual phenotype [35–37]. Patients with gynecological pathologies contributing to infertility, such as endometriosis, display markedly reduced decidualization and impaired uterine receptivity [38]. To access ARID1A function in stroma cells, we examined the levels of ARID1A in human primary endometrial stromal cells (hESCs) from patients with or without endometriosis by Western blot. All 6 hESCs from women without endometriosis showed strong expression of ARID1A in hESCs from women without endometriosis. Interestingly, 5 of 6 hESCs from women with endometriosis did not detect ARID1A protein (Fig 3A). This result suggests that ARID1A loss may cause an impaired decidualization in patients with endometriosis. Therefore, we next examined the role of *Arid1a* in decidualization.

We next examined the ability of *Arid1a* ^*d/d*^ mice to undergo decidualization after artificial hormonal induction. Ovariectomized *Arid1a* ^*f/f*^ and *Arid1a* ^*d/d*^ mice were treated with E2+P4, and the uteri were mechanically stimulated to mimic the presence of an implanting embryo and to induce decidualization [34]. Control mice showed a decidual uterine horn that responded well to this artificial induction. However, *Arid1a* ^*d/d*^ mice exhibited a significant defect of decidual response. The weight ratio of stimulated to control horn was highly decreased in *Arid1a* ^*d/d*^ mice compared to *Arid1a* ^*f/f*^ mice (Fig 3B). Histological analysis confirmed that well-developed decidual cells were detected in the decidual uterine horn of *Arid1a* ^*f/f*^ mice, while differentiation of uterine stromal cells to decidual cells was not observed in the decidual uterine horn of *Arid1a* ^*d/d*^ mice (Fig 3C). In addition, the expression of known markers of decidualization, *Bmp2*, *Fst*, and *Fkbp5*, were significantly decreased in the decidual uterine horn of *Arid1a* ^*d/d*^ mice compared to the decidual uterine horn of *Arid1a* ^*f/f*^ mice (Fig 3D). These data show that *Arid1a* ^*d/d*^ mice have a decidualization defect.

### Aberrant activation of proliferation in the uterine epithelial cells of *Arid1a* ^*d/d*^ mice

**Fig 4:**
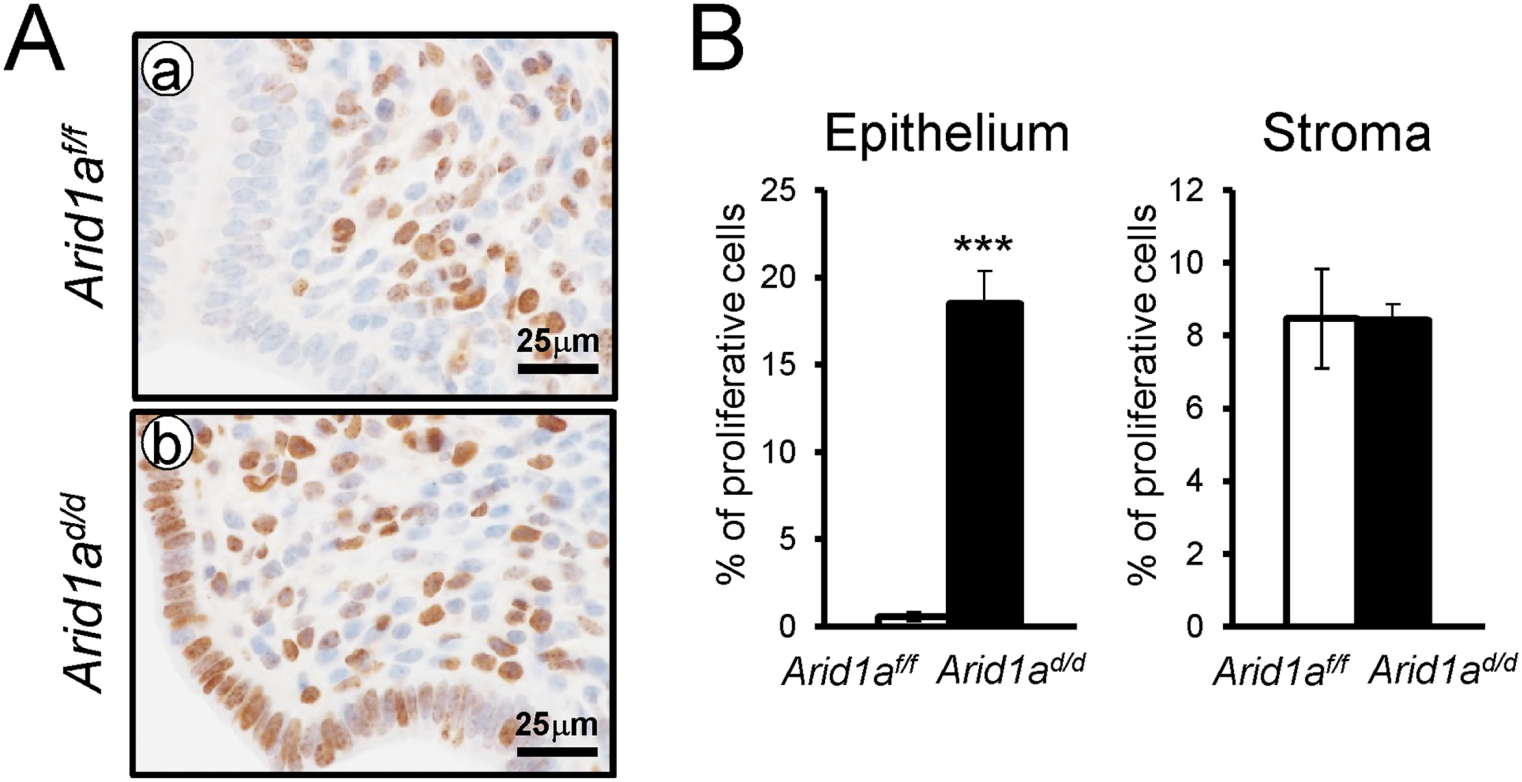
The epithelial proliferation is highly increased in *Arid1a* ^*d/d*^ mice. (A) Immunohistochemical analysis of Ki67 in *Arid1a* ^*f/f*^ (a) and *Arid1a* ^*d/d*^ mice (b). (B) Quantification of Ki67 positive cells in epithelial and stroma cells. The results represent the mean ± SEM. ***, *p* < 0.001.

In normal pregnant uteri, abundant proliferation was detected in epithelial cells and stromal cells at 2.5 dpc. Proliferation is markedly reduced in epithelial cells at 3.5 dpc for embryo attachment [39]. To determine whether a defect of embryo attachment is caused by an alteration in cell proliferation, we examined the expression of Ki67, a proliferative marker, at 3.5 dpc by immunohistochemistry. Ki67 immunohistochemistry showed that proliferation was highly increased in uterine epithelial cells of *Arid1a* ^*d/d*^ mice compared to *Arid1a* ^*f/f*^ mice (Fig 4). These results suggest that abnormal epithelial proliferation in *Arid1a* ^*d/d*^ mice is one of the causes of the embryo attachment defect.

### Estrogen receptor activity is enhanced in the uterine epithelium of *Arid1a* ^*d/d*^ mice

**Fig 5:**
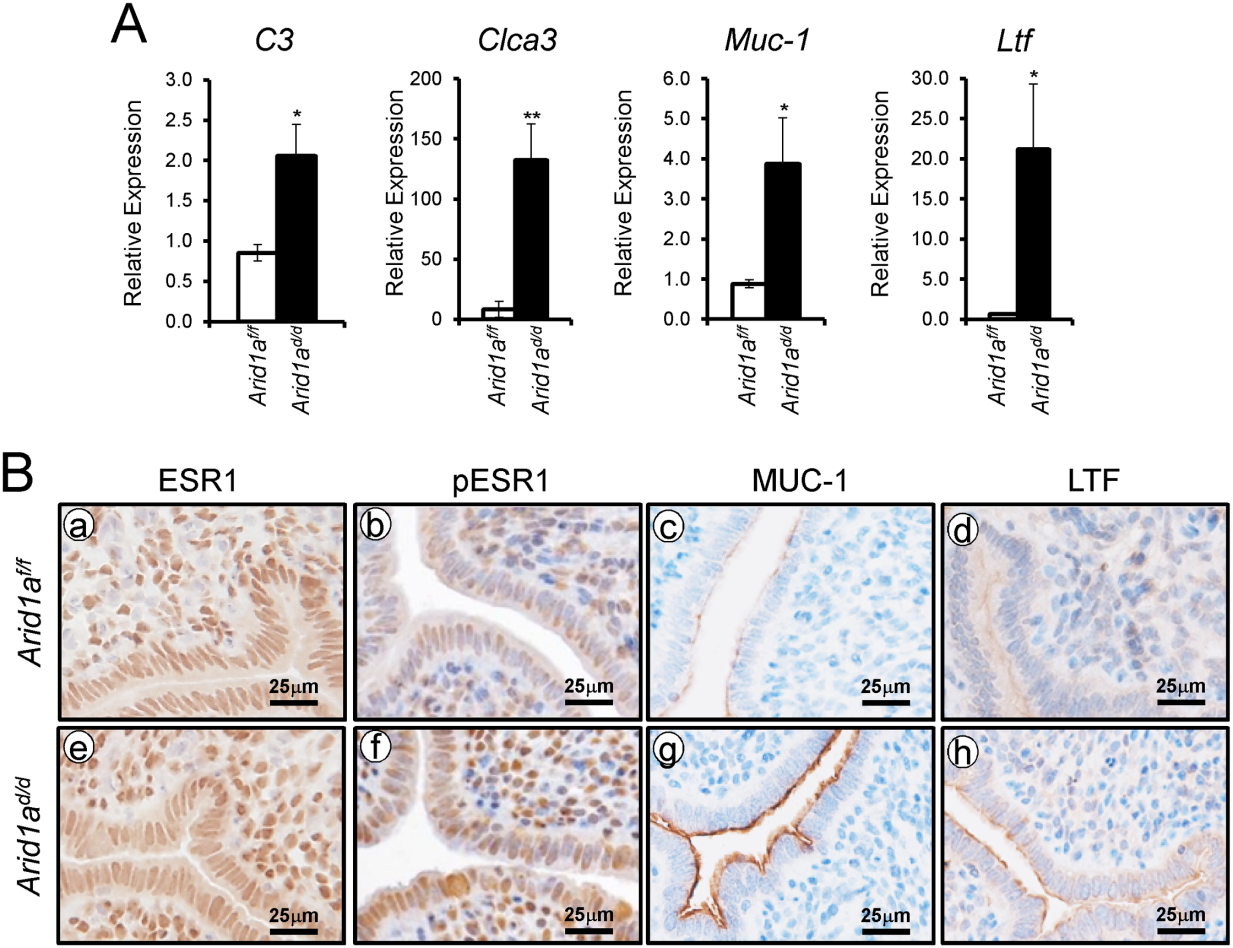
An increase of E2 signaling in *Arid1a* ^*d/d*^ mice. (A) Real-time RT-PCR analysis of *C3*, *Clca3*, *Muc-1*, and *Ltf* were performed on uteri of *Arid1a* ^*f/f*^ and *Arid1a* ^*d/d*^ mice at 3.5 dpc. The results represent the mean ± SEM of six independent mouse sets. *, *p* < 0.05; **, *p* < 0.01. (B) Immunohistochemical analysis of ESR1 (a and e), pESR1 (b and f), MUC-1 (c and g) and LTF (d and h) in uteri of *Arid1a* ^*f/f*^ and *Arid1a* ^*d/d*^ mice at 3.5 dpc.

E2 promotes epithelial cell proliferation in the uterus [6]. Since an increase of epithelial proliferation is observed in *Arid1a* ^*d/d*^ mice, we further investigated whether excess E2 signaling is caused by *Arid1a* ablation. To address excess E2 signaling, the expression of E2 responsive genes, *C3*, *Clca3*, *Muc-1*, and *Ltf*, were examined by real-time RT-PCR analysis. The expression of *C3*, *Clca3*, *Muc-1*, and *Ltf* were highly increased in *Arid1a* ^*d/d*^ mice compared to *Arid1a* ^*f/f*^ mice (Fig 5A). An increase of phospho-ESR1, MUC1 and LTF protein expression was detected in the epithelium of the *Arid1a* ^*d/d*^ mice compared to the *Arid1a* ^*f/f*^ mice, but ESR1 was not changed between the mice (Fig 5B). *Arid1a* ^*f/f*^ mice had an average of 71.39± 2.58%, meanwhile *Arid1a* ^*d/d*^ mice had an average 69.02± 2.90% of positive stromal pESR1 cells. There are no significant differences. These results demonstrate that estrogen receptor activity is enhanced in the uterine epithelial cells of the *Arid1a* ^*d/d*^ mice.

### Epithelial PGR is reduced in the uteri of *Arid1a* ^*d/d*^ mice

**Fig 6:**
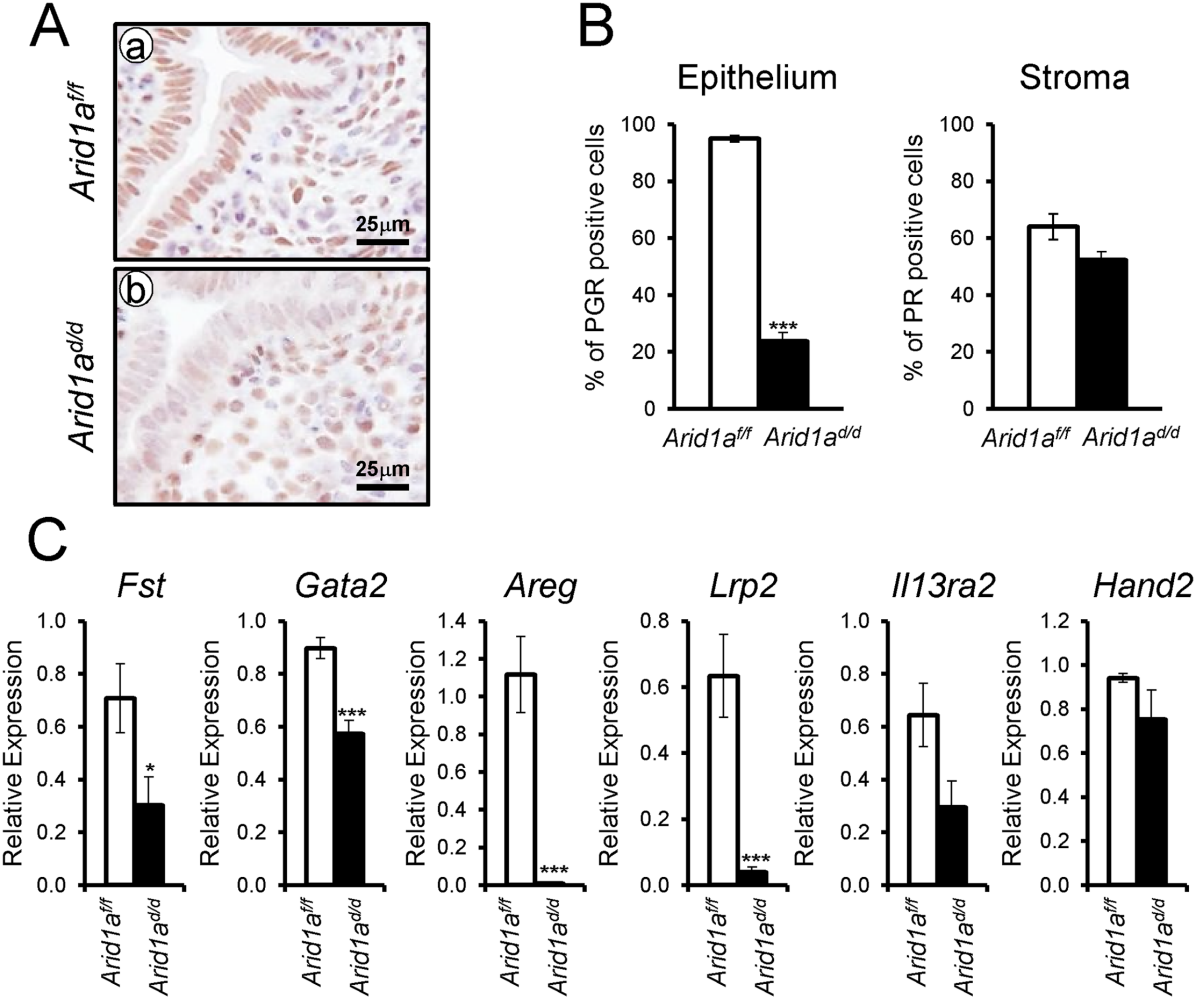
Decreased epithelial PGR expression in *Arid1a* ^*d/d*^ mice. (A) Immunohistochemical analysis of PGR in *Arid1a* ^*f/f*^ (a) and *Arid1a* ^*d/d*^ mice (b). (B) Quantification of PGR positive cells in epithelial and stroma cells. The results represent the mean ± SEM. ***, *p* < 0.001. (C) Real-time RT-PCR analysis of *Fst*, *Gata2*, *Areg*, *Lrp2*, *Il13ra2*, and *Hand2* were performed on uteri of *Arid1a* ^*f/f*^ and *Arid1a* ^*d/d*^ mice at 3.5 dpc.

Since excess E2 signaling is detected in the *Arid1a* ^*d/d*^ mice, we next investigated whether *Arid1a* ablation altered the expression of PGR. We performed PGR immunohistochemistry and real-time RT-PCR to assess the expression of PGR and its target genes in *Arid1a* ^*d/d*^ mice. Interestingly, epithelial PGR expression was highly reduced in *Arid1a* ^*d/d*^ mice compared to control mice (Fig 6A and 6B). The mRNA expression level of epithelial P4 target genes, *Fst*, *Gata2*, *Areg*, and *Lrp2* were highly downregulated in *Arid1a* ^*d/d*^ mice. However the expression of *Il13ra2* and *Hand2* which are known as stromal P4-target genes were not changed (Fig 6C). These results suggest that *Arid1a* mediates estrogen activity by regulating epithelial PGR expression.

### ARID1A suppresses E2 induced epithelial cell proliferation through KLF15

**Fig 7:**
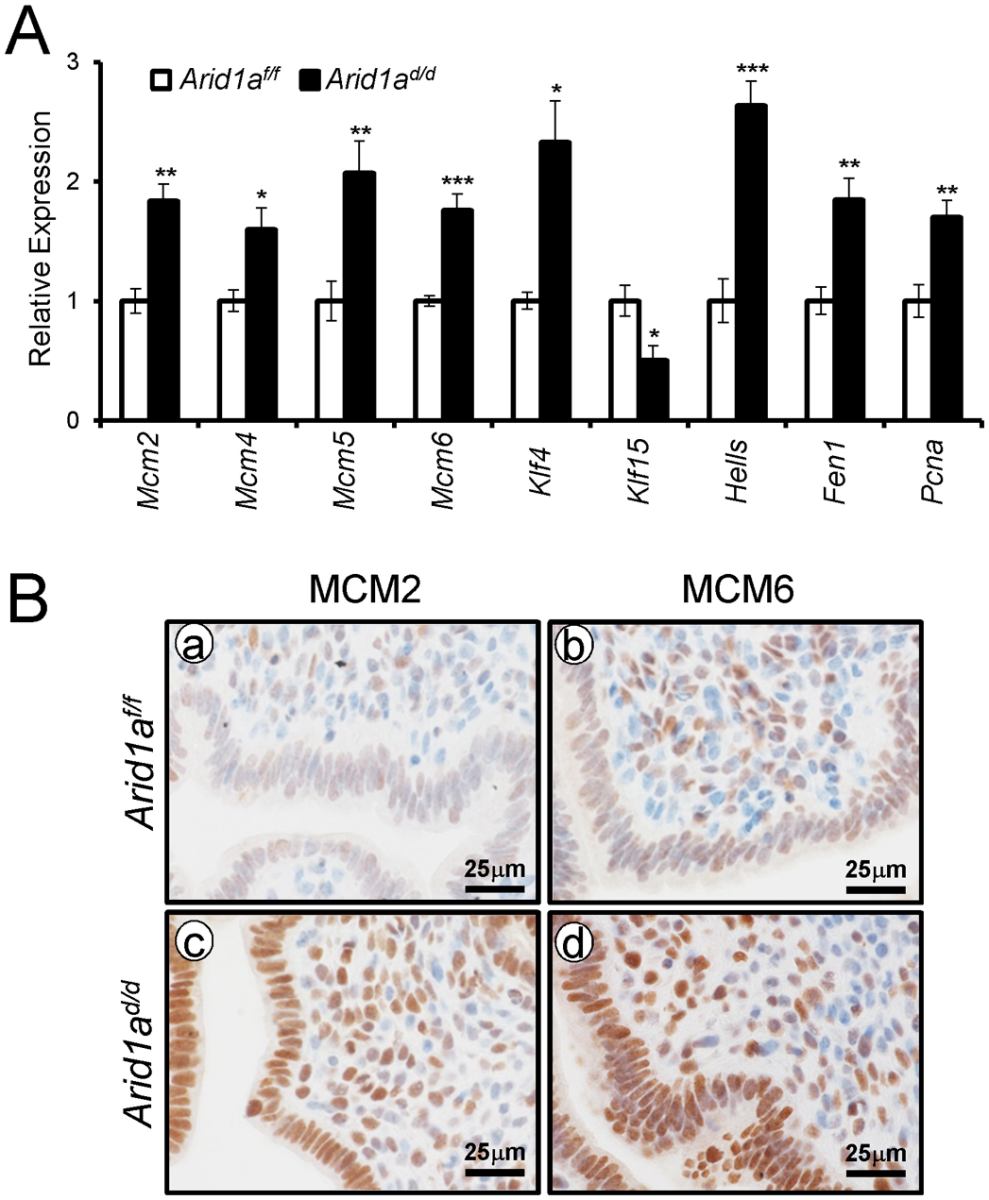
The confirmation of dysregulated genes by *Arid1a* ablation. (A) The validation of microarray analysis by qPCR in *Arid1a* ^*f/f*^ and *Arid1a* ^*d/d*^ mice at 3.5 dpc. The results represent the mean ± SEM. *, *p* < 0.05, **, *p* < 0.01, ***, *p* < 0.001. (B) Immunohistochemical analysis of MCM2 and MCM6 in the uteri of *Arid1a* ^*f/f*^ (a and b) and *Arid1a* ^*d/d*^ (c and d) mice at 3.5 dpc.

In order to identify the pathways that *Arid1a* regulates at implantation, we performed high density DNA microarray analysis on the uteri from *Arid1a* ^*f/f*^ and *Arid1a* ^*d/d*^ mice at 3.5 dpc (GEO accession number: GSE72200). The microarray analysis showed that 1,358 were more highly expressed in *Arid1a* ^*d/d*^ mice and 1,198 genes were decreased by more than 1.5-fold. From the pathway analysis using Ingenuity Pathway Analysis (QIAGEN, Redwood City, CA), the altered pathways including cell-cycle control, DNA replication, and modification processes were identified (Table 1 and S3 Table). The results have been validated by qPCR analysis (Fig 7A). The immunohistochemistry results showed that the levels of MCM2 and MCM6 were increased in *Arid1a* ^*d/d*^ mice at 3.5 dpc (Fig 7B).

**Table 1:** Dysregulation of genes associated with cell cycle and DNA replication whose transcripts are up-regulated by *Arid1a* ablation.

Symbol	Name	Fold Change
**DNA pre-replicative complex licensing genes**		
*Mcm2*	Minichromosome maintenance deficient 2	2.57
*Mcm3*	Minichromosome maintenance deficient 3	1.98
*Mcm4*	Minichromosome maintenance deficient 4	1.90
*Mcm5*	Minichromosome maintenance deficient 5	2.52
*Mcm6*	Minichromosome maintenance deficient 6	2.88
**Chromatin assembly and modification genes**		
*Chaf1b*	Chromatin assembly factor 1, subunit B (p60)	2.17
*Hells*	Helicase, lymphoid specific	3.91
**DNA replication genes**		
*Fen1*	Flap structure specific endonuclease 1	2.12
*Pcna*	Proliferating cell nuclear antigen	1.72
**Other cell-cycle-related genes**		
*Mad2l1*	MAD2 (mitotic arrest deficient, homolog)-like 1	2.54
*Myb*	Myeloblastosis oncogene	4.16
*Tk1*	Thymidine kinase 1	2.04
*Ccnb1*	Cyclin B1	1.67
*Klf4*	Kruppel-like factor 4	1.72
*Klf15*	Kruppel-like factor 15	**-1.61**

**Fig 8:**
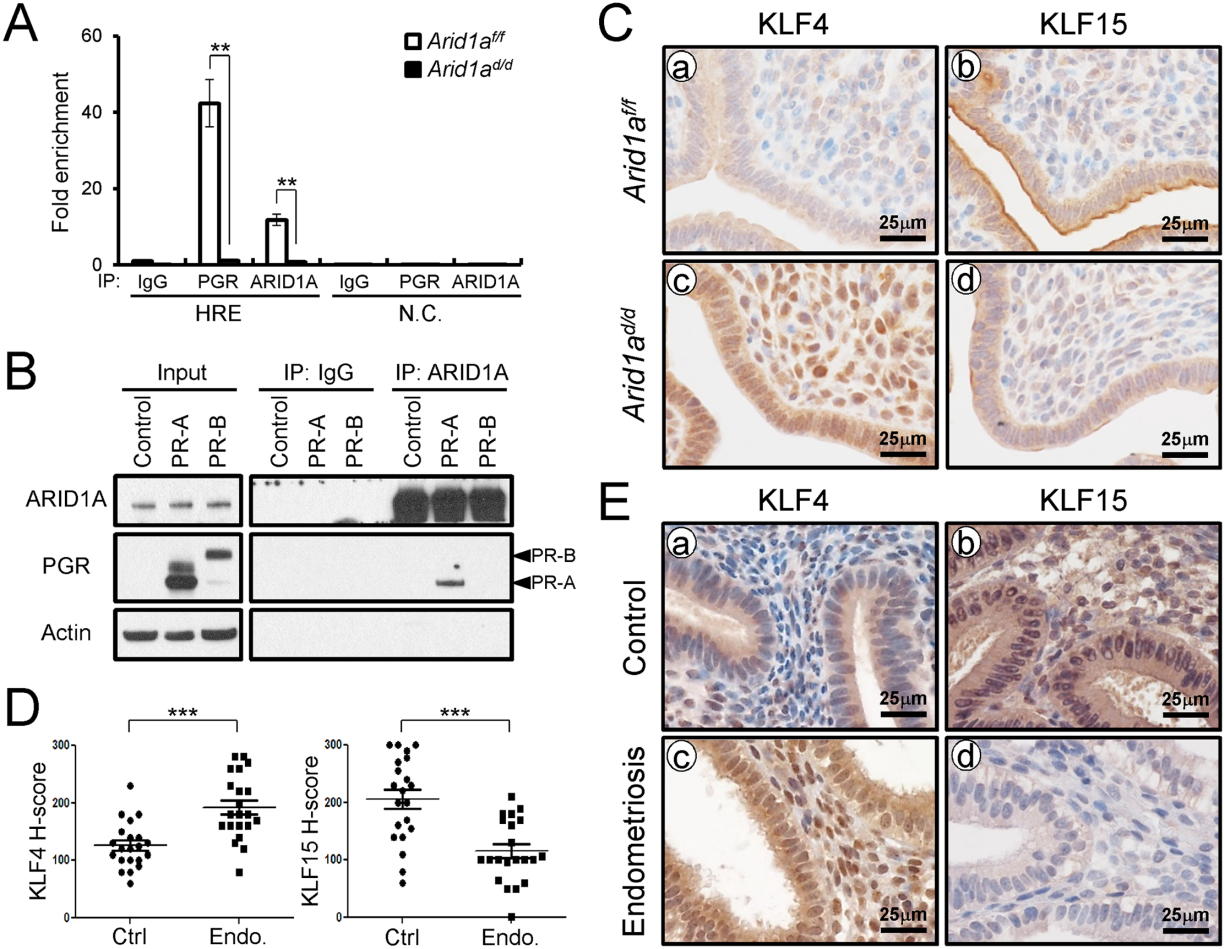
ARID1A regulates epithelial proliferation via modulating KLF15 expression with PGR (A) ChIP assay performed with uterine chromatin isolated *Arid1a* ^*f/f*^ and *Arid1a* ^*d/d*^ mice at 3.5 dpc using IgG, PGR, and ARID1A antibodies followed by qPCR. The results represent the mean ± SEM. **, *p* < 0.01. (B) Protein interaction between ARID1A and PGR was examined by immunoprecipitation and transient transfection in Ishikawa cells. (C) Immunohistochemical analysis of KLF4 and KLF15 were performed on uteri of *Arid1a* ^*f/f*^ (a and b) and *Arid1a* ^*d/d*^ (c and d) mice at 3.5 dpc. (D) The immunohistochemical histological score (H-score) of KLF4 and KLF15 proteins (n = 21 per group at secretory phase). The results represent the mean ± SEM. *** *p*<0.001. (E) The level of KLF4 and KLF15 in human endometrium without (a and b) and with (c and d) endometriosis were performed by immunohistochemical analysis.

Two Kruppel-like factors (KLFs) have been implicated in E2 and P4 modulation of uterine proliferation [40,41]. *Klf4* is increased by E2 and promotes DNA replication, whereas *Klf15* is increased by P4 and inhibits growth via regulation of *Mcm2* [41]. The down-regulation of PGR by ARID1A loss coincides with the down-regulation of *Klf15* transcript abundance, which led to the hypothesis that ARID1A positively regulates *Klf15* expression with PGR. To determine whether ARID1A and PGR bind to the putative *Klf15* promoter, ChIP was performed on uterine chromatin from *Arid1a* ^*f/f*^ and *Arid1a* ^*d/d*^ mice at 3.5 dpc. ChIP analysis exhibited that recruitment of PGR on HRE is significantly decreased by the absence of *Arid1a* indicating that *klf15* is directly regulated by ARID1A and PGR (Fig 8A). We examined whether ARID1A physically interacts with PR-A or PR-B protein using immunoprecipitation analysis. We transfected with PGR constructs expressing either human PR-A or PR-B into Ishikawa cells. The lysates were then immunoprecipitated with anti-ARID1A antibodies, and then performed western blot analysis using anti-PGR antibodies. The immunoprecipitation results showed that ARID1A physically interacts with PR-A, not PR-B (Fig 8B). Next, we examined the protein levels of KLF4 and KLF15 to determine whether their dys-regulation might contribute to aberrant epithelial proliferation in *Arid1a* ^*d/d*^ mice. The expression of KLF4 was remarkably increased in *Arid1a* ^*d/d*^ mice compared to *Arid1a* ^*f/f*^ mice while the expression of KLF15 was decreased in *Arid1a* ^*d/d*^ mice (Fig 8C). These results suggest that ARID1A regulates transcriptional activation of *KLF15* through physical interaction with PR-A.

To better understand the integration of ARID1A in endometriosis, immunohistochemistry analysis for KLF4 and KLF15 was performed with eutopic endometrium from secretory phase women with and without endometriosis (Fig 8D and 8E). As shown in the *Arid1a* ^*d/d*^ mice, eutopic endometrium from women with endometriosis showed increased KLF4 levels compared to control endometrium. The expression of KLF15 was very weak in eutopic endometrium from women with endometriosis, while its expression was strong in endometrial cells in control endometrium. These data suggest that KLF15 is a downstream mediator of the anti-proliferative action of P4 on E2-induced epithelial cell proliferation and ARID1A regulates KLF15 expression with PGR.

## Discussion

Somatic ARID1A mutations are uniquely associated with endometriosis-related ovarian neoplasms [42–45]. ARID1A is located within chromosomal region 1p36, a region frequently deleted in a variety of human cancers [46,47]. Indeed, many studies have analyzed ARID1A expression in a variety of human cancers and demonstrated loss of ARID1A expression [43,48–50]. *ARID1A* was mutated in 46% of ovarian clear-cell carcinomas and 30% of endometrioid ovarian carcinomas [28,31]. Loss of *ARID1A* is also frequent in endometrial carcinoma [51–53]. Interestingly, *Arid1a* ^*d/d*^ mice showed aberrant active epithelial proliferation, but did not develop endometrial hyperplasia or cancer. Our results suggest that *Arid1a* loss alone is not enough to lead to the development of endometrial cancer.

Endometriosis is a common cause of infertility [54]. However, the roles of ARID1A in infertility and endometrial function have not been studied. In the present study, we report that ARID1A protein levels are significantly lower in the eutopic endometrium of women with endometriosis compared to women without endometriosis, and mice with conditional ablation of *Arid1a* in PGR positive cells (*Arid1a* ^*d/d*^) were sterile. These results suggest a relationship between ARID1A loss and infertility. Since PGR^Cre^ mice show Cre recombinase activity in the pituitary, ovary, uterus and mammary glands, these mice may have infertility due to a defect of *Arid1a* in any of these tissues [55]. *Arid1a* ^*d/d*^ mice had normal ovarian function indicating that the conditional loss of *Arid1a* in the granulosa cells of the ovary did not influence ovarian function. Although our study does not rule out a pituitary defect, a failure of embryo attachment and decidualization in *Arid1a* ^*d/d*^ mice suggest that the fertility defect is primarily due to a uterine defect.

Receptivity in the mouse endometrium is dependent on ovarian steroid hormones. On 0.5 and 1.5 dpc, E2 promotes uterine epithelial cell proliferation and growth. On 2.5 dpc, P4 inhibits this epithelial proliferation, promoting receptivity, and inducing stromal cell proliferation [56]. We observed increased proliferation in the epithelium of *Arid1a* ^*d/d*^ mice at 3.5 dpc indicating enhanced epithelial E2 signaling. It is reported that enhanced epithelial E2 activity leads to implantation failure [57,58]. We also showed that conditional ablation of *Arid1a* results in elevated levels of phospho-ESR1, the active form of ESR1, and ESR1 target genes, *C3*, *Clca3*, *Muc-1*, and *Ltf* which plays an essential role in uterine receptivity and embryo attachment [59–61]. These results indicate that the attachment defect observed in *Arid1a* ^*d/d*^ mice is due to a failure of P4 to squelch E2 signaling in luminal epithelium as indicated by altered expression of MUC-1 and LTF which is tightly regulated at pre-implantation [62,63]. COUP-TF II mediates *Bmp2* expression by controlling ESR1 activity in the murine uterus [58], and its expression is promoted by SWI/SNF in vascular endothelium [64]. Thus, we examined the expression of COUP-TF II in *Arid1a* ^*d/d*^ mice at 3.5 dpc by immunohistochemistry. COUP-TF II immunostaining were not different in uterine stroma cells of *Arid1a* ^*d/d*^ mice compared to *Arid1a* ^*f/f*^ mice.

Previous studies have shown that PGR has an important role in inhibiting E2 induced epithelial proliferation [65,66]. A decrease of epithelial PGR is observed in *Arid1a* ^*d/d*^ mice resulting in down-regulated PGR target genes, *Fst*, *Gata2*, *Areg*, and *Lrp2* [67]. *Gata2*, *Areg*, and *Lrp2* localization is limited to the epithelium [68–70]. We examined the expression of *Il13ra2* and *Hand2* [71] which are known as stromal P4-target genes by RT-qPCR. These mRNA levels were not different between *Arid1a* ^*f/f*^ and *Arid1a* ^*d/d*^ mice. These data suggest that *Arid1a* is mainly functional in the epithelial cells at the peri-implantation stage. However, ARID1A is expressed both in epithelium and stroma. It will be useful to ascertain its cell type specific role using epithelium cell specific knockout mouse models [72–74]. Stromal functions including proliferation and the expression of *Hand2* and *Il13ra2* are not altered at the peri-implantation stage of *Arid1a* ^*d/d*^ mice. However, its function in stroma cells may play an important role because the phenotypes of *COUP-TFII* [58] and *Hand2* [71] knockout mice have a similar phenotype.

SWI/SNF complexes interact with several nuclear receptors, including glucocorticoid receptors, estrogen receptors and vitamin D3 receptors, to activate transcription of specific target genes [47,75]. Several studies have linked SWI/SNF and ARID1A to transcriptional regulation, particularly nuclear hormone-induced transcription and expression of cell-cycle regulators [76–78]. Our results suggest that ARID1A is pivotal to regulating transcription of PGR target genes to prepare receptivity in the uterus. Loss of ARID1A may have many effects on SWI/SNF complexes that lead to transcriptional dysfunction, including disruption of nucleosome sliding activity, assembly of variant SWI/SNF complexes, targeting to specific genomic loci, and/or recruitment of coactivator/corepressor activities. An impaired P4 response is seen in the endometrium of women with infertility and endometriosis [79–81]. However, molecular mechanisms of aberrant PGR function in uterine diseases remain uncertain. Although this study has not clearly addressed why the epithelium PGR was decreased in the *Arid1a* knockout uterus, our results show that ARID1A regulates PGR signaling to prepare receptivity in the uterus. ARID1A may regulate stability of PGR proteins. However, it is also possible that PGR is a target gene of ARID1A. Further investigation is required to elucidate the exact mechanism underlying a possible regulatory role of ARID1A in the regulation of steroid hormone signaling.

Interestingly, this *Arid1a* ^*d/d*^ phenotype is similar to *Wnt7a-Cre PGR* ^*f/-*^ mice, with epithelial-specific ablation of PGR. *Wnt7a-Cre PGR* ^*f/-*^ mice were infertile due to defects in embryo attachment, stromal cell decidualization, the inability to cease estrogen-induced epithelial cell proliferation, and the lack of P4 regulated expression of its epithelial target genes [11]. Stromal-epithelial cross talk is critical in pregnancy [11,82] and P4 achieves inhibition of E2-induced epithelial cell proliferation by coordinating stromal-epithelial cross-talk [7,9,10]. The Stromal functions including proliferation and the expression of *Hand2* and *Il13ra2* in *Arid1a* ^*d/d*^ mice are not altered at 3.5 dpc. These results suggest that *Arid1a* is mainly functional in the epithelial cells of the peri-implantation stage. An epithelium cell specific *Arid1a* knockout mouse model using Wnt7a-cre or Lactoferrin-iCre mouse [72–74] will be an invaluable approach to ascertain its cell type specific role. However, ARID1A is expressed both in epithelium and stroma. Its function in stroma cells may play an important role because the phenotypes of *Hand2 [71]* and *COUP-TFII [58]* knockout mice have similar phenotypes. Determining the role of *Arid1a* in stromal-epithelial cross talk will be critical in understanding the role of steroid hormone signaling and dysfunction associated with infertility and endometriosis.

To investigate the global impact on gene expression caused by the loss of *Arid1a*, we conducted microarrays at the peri-implantation stage and identified over 2,500 misregulated genes in the absence of *Arid1a*. Dr. Pollard’s group demonstrated that P4 blocks E2-induced DNA synthesis through the inhibition of replication licensing including MCM proteins [83,84]. There is a significant overlap in the list of genes associated with cell cycle and DNA replication between Dr. Pollard’s and our microarray results. In the uterine epithelium, E2 stimulates the expression of the MCMs while P4 inhibits the transcript abundance of MCM 2 to 6 [83,85]. The immunohistochemistry results showed aberrant overexpression of MCM2 and MCM6 in the epithelial cells of *Arid1a* ^*d/d*^ mice at the peri-implantation stage. A similar action can be ascribed to P4 and E2 in human endometrial epithelium as a loss of MCM proteins occurs in the secretory phase, and therefore P4 dominated this phase of the menstrual cycle [81,86]. However, aberrant overexpression of MCM2 and MCM6 may cause abnormal epithelial proliferation and early pregnancy loss. Despite the importance of this regulation in mice and humans, the molecular basis for the P4 and E2 regulation of DNA replication licensing is not understood. Our results demonstrate that ARID1A loss results in increased E2 sensitivity of the uterus in the presence of P4.

Kruppel-like factors (KLF) family play important roles in cellular proliferation, survival, differentiation, pluripotency, and epithelial-to-mesenchymal interactions [87]. The members of the KLF family are ubiquitously expressed in the uterus and have been increasingly implicated as critical co-regulators and integrators of steroid hormone actions [88]. The expression of KLF9 is lower in eutopic endometrium of women with endometriosis and endometrial KLF9 deficiency promotes endometriotic lesion establishment by the coincident deregulation of Notch-, Hedgehog-, and steroid receptor-regulated pathways [89–91]. However, the expression of *Klf9* is not altered in *Arid1a* ^*d/d*^ mice.

*Klf4* and *Klf15* play a critical role in uterine proliferation by modulating E2 and P4 [40,41]. E2 induces *Klf4* expression and promotes DNA replication, whereas *Klf15* is induced by P4 and inhibits growth via regulation of *Mcm2* [41]. Therefore, we focused on transcriptional regulation of *Klf15* as an E2 regulated transcription factor. KLF15 binds to the *MCM2* promoter in a P4 and E2 dependent fashion, which negatively regulates RNA Pol II association [41]. *Klf15* expression suppresses E2 mediated *MCM2* transcription. In vivo, *Klf15* expression in the E2 exposed uterus mimics P4 action by inhibiting *Mcm2* expression and epithelial cell DNA synthesis. ChIP analysis demonstrated that ARID1A and PGR directly bind to the PRE region of *Klf15* promoter. Immunohistochemistry analysis showed an increase of KLF4 and a decrease of KLF15 expression in *Arid1a* ^*d/d*^ mice at the peri-implantation stage. These data establish *Klf15* as a downstream mediator of the anti-proliferative action of P4 on E2-induced epithelial cell proliferation and ARID1A regulates E2-induced epithelial proliferation by modulating *klf15* expression with PGR.

Following embryo attachment, the uterus again changes during a process known as decidualization whereby the epithelium undergoes apoptosis and the stroma proliferates and differentiates into a more epitheliod cell type [66]. We demonstrated that *Arid1a* ^*d/d*^ mice exhibited a defect of the decidual response. *Bmp2* and *Fkbp4* null females exhibited a defect of implantation and decidualization suggesting a critical role in decidualization [92–94]. *Fst* is a known *Bmp2* target [95]. The decidualization markers, *Bmp2*, *Fst*, and *Fkbp5*, were significantly decreased in *Arid1a* ^*d/d*^ mice indicating that uterine specific ablation of *Arid1a* caused a significant decidualization defect.

In conclusion, ARID1A has a key role in implantation and decidualization, and that ARID1A expression is lost in endometriosis. Ablation of *Arid1a* affects epithelial proliferation in part via dysregulating KLF15 expression with PGR. Aberrant proliferative conditions of the human endometrium are common. Inappropriate proliferation of the uterus is one cause leading to endometriosis [96]. Determining the mechanism of *Arid1a* in uterine dysfunction associated with infertility and endometriosis will be critical to understanding both of these common uterine diseases for future therapy.

## Materials and Methods

### Ethics statement

The study has been approved by Institutional Review Committee of Michigan State University (IRB number: 07–712; r047700), Greenville Health System (IRB number: Pro00013885 and Pro00000993) and University of North Carolina (IRB number: 05–1757), and written informed consent was obtained from all participants. All protocols related to animals were overseen and approved by the Institutional Animal Care and Use Committee at Michigan State University (AUF number: 11/13-248-00). Animals were maintained in a designated animal care facility in accordance with Michigan State University’s institutional guidelines.

### Human endometrium samples

The human endometrial samples were collected from Michigan State University’s Center for Women’s Health Research Female Reproductive Tract Biorepository, the Greenville Hospital System, and the University of North Carolina. Samples were collected as previously reported [97,98]. Briefly, to compare gene expression patterns of eutopic endometrium between those with and without endometriosis, 28 samples were collected from proliferative, early, mid, and late secretory phases (n = 7 per phase). For endometriosis eutopic endometrium, 28 samples were collected from proliferative, early, mid, and late secretory phases (n = 7 per phase). Endometrial biopsies were obtained at the time of surgery from regularly cycling women between the age of 18 and 45. The presence or absence of disease was confirmed during surgery. Women laparoscopically negative for this disease were placed into the control group, whereas women laparoscopically positive for this disease were placed in the endometriosis group. Use of an intrauterine device (IUD) or hormonal therapies in the 3 months preceding surgery was exclusionary for this study. Histologic dating of endometrial samples was done based on the criteria of Noyes [99] and confirmed by subsequent histo-pathological examination by an experienced Fertility specialist (B.A.L.).

Isolation of human primary endometrial stromal cells (hESCs) has been previously described [39]. hESCs were isolated from proliferative phase patients with or without endometriosis. Proteins were extracted using lysis buffer (150 mM NaCl, 0.125% Nonidet P-40 (vol/vol), 2.5 mM EDTA, and 10 mM Tris-HCl (pH 7.4) included with both a phosphatase inhibitor cocktail (Sigma Aldrich, St. Louis, MO) and a protease inhibitor cocktail (Roche, Indianapolis, IN). Twenty μg of protein lysates were electrophoresed via SDS-PAGE and were then transferred onto polyvinylidene difluoride membrane (Millipore Corp., Bedford, MA). Western blot analysis was performed using anti-ARID1A (Abnova, Neihu District, Taipei City, Taiwan) and anti-Actin (Santa Cruz) antibodies.

### Animals and tissue collection

*Arid1a* conditional knockout mice were generated by crossing *Pgr* ^*cre/+*^ [55] with *Arid1a* ^*f/f*^ [8] mice (*Pgr* ^*cre/+*^ *Arid1a* ^*f/f*^; *Arid1a* ^*d/d*^). Pregnant uterine samples were obtained by mating *Arid1a* ^*f/f*^ and *Arid1a* ^*d/d*^ female mice with C57BL/6 male mice with the morning of a vaginal plug being designated as 0.5 dpc. Mice were sacrificed at 3.5, 4.5 and 5.5 dpc and the number of implantation sites identified on 5.5 dpc. The level of progesterone and estrogen in serum were analyzed by the University of Virginia Center for Research in Reproduction Ligand Core. Uterine tissues were snap-frozen at the time of dissection and either stored at -80°C for RNA/protein extraction or fixed with 4% (vol/vol) paraformaldehyde for histology. For the fertility studies, adult female *Arid1a* ^*f/f*^ and *Arid1a* ^*d/d*^ female mice were placed with wild-type male mice (n = 9). The mating cages were maintained for 6 months and the number of litters and pups born during that period was recorded. For ovulation and fertilization test, female mice (n = 3 per genotype) were superovulated by i.p. injection of 5 IU of PMSG (Fisher Sci.) followed 48 h later by 5 IU of hCG (Sigma-Aldrich) and mated with wild-type male mice. The following morning (0.5dpc), ovulated eggs were flushed from the oviducts on 1.5 dpc.

### Induction of decidualization

The hormonally induced decidual response has been previously described [100]. Briefly, *Arid1a* ^*f/f*^ and *Arid1a* ^*d/d*^ female mice at 6-weeks of age were ovariectomized (n = 3 per genotype). Two weeks post ovariectomy, *Arid1a* ^*f/f*^ and *Arid1a* ^*d/d*^ were subjected to the following hormonal regimen: 100 ng of E2 per day for three days; two days rest; then, three daily injections of 1 mg of P4 + 6.7 ng of E2. Six hours following the third P4 and E2 injection, the left uterine horn was mechanically stimulated by scratching the full length of the anti-mesometrial side with a burred needle. The other horn was left unstimulated as a control. Daily injections of P4 (1 mg/mouse) + E2 (6.7 ng/mouse) were continued for five days to maximize the decidual response. Then, mice were sacrificed on day 5. The uteri were then excised, weighed and fixed in 4% paraformaldehyde for histological analysis.

### Quantitative real-time PCR

RNA was extracted from the uterine tissues using the RNeasy total RNA isolation kit (Qiagen, Valencia, CA, USA). mRNA expression levels of decidual marker genes (*Bmp2*, *Fst*, and *Fkbp5*), *Esr1* target genes (*C3*, *Clca3*, *Muc-1*, and *Ltf*) and *Pgr* target genes (*Fst*, *Gata2*, *Areg*, *Lrp2*, *Il13ra2*, and *Hand2*) were measured by real-time PCR TaqMan analysis using an Applied Biosystems StepOnePlus system according to the manufacturer's instructions (Applied Biosystems, Foster City, CA, USA) and using pre-validated probes, primers, 18S RNA and Universal Master mix reagent purchased from Applied Biosystems (Applied Biosystems). Template cDNA was produced from 1 μg of total RNA using random hexamers and MMLV Reverse Transcriptase (Invitrogen Corp.). All real-time PCR was done by using three independent RNA sets. The mRNA quantities were normalized against 18S RNA using ABI rRNA control reagents.

### Immunohistochemistry

Uterine sections from paraffin-embedded tissues were cut at 5 μm and mounted on silane-coated slides, deparaffinized, and rehydrated in a graded alcohol series before blocking with 10% normal goat serum in PBS (pH 7.5) and incubating with primary antibody diluted in 10% normal goat serum in PBS (pH 7.5) overnight at 4°C at the following dilutions: 1:500 for anti-ARID1A (Sc-98441, SantaCruz), 1:100 for anti-Ki67 (ab15580, Abcam), anti-ESR1 (DAKO Corp.), 1:100 for anti-phospho-ESR1 (Ab31477, Abcam), 1:1000 for MUC-1 (ab15481, Abcam), 1:2000 for LTF (07–682, Millipore), MA), 1:20000 for MCM2 (Sc-9839, SantaCruz), 1:20000 for MCM6 (Sc-9843, SantaCruz), 1:5000 for KLF4 (Sc-20691, SantaCruz), 1:5000 for FLK15 (ab2647, Abcam), and 1:1000 for anti-total PGR antibody (A0098, DAKO Corp.). On the following day, sections were washed in PBS and incubated with the appropriate species-specific HRP-conjugated secondary antibody (2 μg/ml; Vector Laboratories) for 1 hr at room temperature. Immunoreactivity was detected using the Vectastain Elite DAB kit (Vector Laboratories). A semiquantitative grading system (H-score) was used to compare the immunohistochemical staining intensities as previously described [101]. The number of PGR and Ki67-positive cells was counted in 200 epithelial cells and eight random fields of stromal cells.

### Microarray analysis

Biotinylated cRNA were prepared according to the standard Affymetrix protocol from 500ng total RNA (Expression Analysis Technical Manual, 2001, Affymetrix). Following fragmentation, 15 ug of aRNA were hybridized for 16 hr at 45C on GeneChip Mouse Genome Array. GeneChips were washed and stained in the Affymetrix Fluidics Station 450. GeneChips were scanned using the Affymetrix GeneChip Scanner 3000 7G. The data were analyzed with RMA using Affymetrix default analysis settings and global scaling as the normalization method. The trimmed mean target intensity of each array was arbitrarily set to 100. The normalized, and log transformed intensity values were then analyzed using GeneSpring GX 12.6 (Agilent technologies, CA). Fold change filters included the requirement that the genes be present in at least 150% of controls for up-regulated genes and lower than 66% of controls for down-regulated genes. Hierarchical clustering data were clustered groups that behave similarly across experiments using GeneSpring GX 12.6 (Agilent technologies, CA). Clustering algorithm was Euclidean distance, average linkage.

### Chromatinimmunoprecipitation (ChIP)

ChIP analysis was conducted by Active Motif (Carlsbad, CA, USA) using frozen mouse uteri of *Arid1a* ^*f/f*^ and *Arid1a* ^*d/d*^ at 3.5 dpc. Uterine tissue samples (approximately 180 mg) were submersed in PBS containing protease inhibitors, cut into small pieces, and treated with fixation solution for 15 min at room temperature. Fixation was stopped by the addition of stop solution for 5 min. The tissue pieces were washed twice with PBS washing buffer, incubated with Chromatin Prep Buffer containing protease inhibitors and PMSF for 10 min on ice, homogenized by glass homogenizer for 30 strock, and finally spun down. Chromatin was isolated from disrupting the cells with a ChIP buffer containing protease inhibitors and PMSF. Lysates were sonicated using a Sonic Dismembrator FB120 (Fisher Scientific, Pittsburgh, PA, USA) to break chromatin into fragments with an average length of 0.5–1 kb. For each ChIP reaction, 100 μg of chromatin was immunoprecipitated by 4 μg of antibodies against PGR (sc7208; Santa Cruz Biotechnology, Santa Cruz, CA, USA) and ARID1A (H00008289-M02; Abnova, Zhongli District, Taoyuan City 320, Taiwan). Following overnight incubation at 4°C, protein G agarose beads were added, and incubation at 4°C continued for another 3 hours. Immune complexes were washed five times with Wash Buffer AM1, eluted from the beads with Elution Buffer AM4 and subjected to RNase treatment and proteinase K treatment. Crosslinks were reversed by incubation for 30 min at 55°C and for 2 hours at 80°C. ChIP DNA was purified by DNA purification column. Purified DNA was used for real-time qPCR. Real-time qPCR was carried out in triplicate using SYBR Green Supermix (Bio-Rad Laboratories, Inc., Hercules, CA). The sequences of the primers used for HRE binding region [40,102] in *Klf15* gene were 5’- TAACCATCTGGGAAGTGGCT-3’ and 5’-GCCACTCTGGAACAGGATG-3’, and for negative control region in *Klf15* gene were 5’-TCTCACTCGGGTGTGAAGCC-3’ and 5’-GTGGGAAGCGATGCACTTTG-3’ (S5 Fig). Immunoprecipitation with normal rabbit IgG was performed as a negative control. The resulting signals were normalized to input DNA.

### Immunoprecipitation analysis

Ishikawa cells were cultured in DMEM/F12 medium (Gibco, Grand Island, NY) containing 10% fetal bovine serum (FBS; Gibco), and 1% penicillin streptomycin (Gibco) at 37°C under 5% CO_2_. The cells were transfected with the human PR-A and PR-B expression vectors using Lipofectamine 2000 (Invitrogen Corp.). The transfected cells were lysed by lysis buffer (150 mM NaCl, 0.125% Nonidet P-40 (vol/vol), 2.5 mM EDTA, and 10 mM Tris-HCl (pH 7.4)) included with both a phosphatase inhibitor cocktail (Sigma Aldrich, St. Louis, MO) and a protease inhibitor cocktail (Roche, Indianapolis, IN). Protein lysates were then immunoprecipitated with ARID1A antibodies (Abnova) with protein A-agarose (Pierce Biotechnology, Rockford, IL) and incubated overnight at 4°C. Immunocomplexes were washed 5 times with 1 ml of lysis buffer and were then subjected to western blot analysis using anti-PGR antibody (SantaCruz). The western blot analysis was performed as described previously [103].

### Statistical analysis

For data with only two groups, the Student’s t test was used. For data containing more than two groups, one way ANOVA was used, followed by Tukey’s post hoc multiple range. All data are presented as means ± SEM. *p* < 0.05 was considered statistically significant. All statistical analyses were performed using the Instat package from GraphPad (San Diego, CA, USA).

## Supporting Information

S1 Table *Arid1a* ^*d/d*^ mice were sterile. (PDF)

S2 Table *Arid1a* ^*d/d*^ has normal ovulation and fertilization. (PDF)

S3 Table List of regulated genes by *Arid1a* ablation. (PDF)

S1 Fig The expression of ARID1A during human menstrual cycle. (TIF)

S2 Fig The expression of ARID1A by real-time RT-PCR and immunohistochemistry in early pregnancy. (A) The expression level of *Arid1a* was measured in uteri of pseudopregnancy. Total RNA used for the RT-PCR assays was prepared from pseudopregnant uteri. (B) The localization pattern of ARID1A by immunohistochemical analysis during early pregnancy.
(TIF)

S3 Fig Generation of uterine specific *Arid1a* ablation in the murine uterus. (A) Western blot analysis of ARID1A and Actin in whole uterine of control (*Arid1a* ^*f/f*^) and *Arid1a* ^*d/d*^ mice at 6 weeks of age. Equal amounts of protein were subjected to SDS-PAGE and Western blot analysis. (B) Immunohistochemical analysis of ARID1A in control and *Arid1a* ^*d/d*^ mice (a and b).
(TIF)

S4 Fig *Arid1a* ^*d/d*^ mice has normal ovary function. (A) Ovarian histology by H&E staining exhibited no difference between control (*Arid1a* ^*f/f*^) (a) and *Arid1a* ^*d/d*^ mice (b). (B) The serum level of E2 and P4 were not different between control (*Arid1a* ^*f/f*^) and *Arid1a* ^*d/d*^ mice at 3.5 dpc. The results represent the mean ± SEM.
(TIF)

S5 Fig Map of PGR (HRE) binding site on the *Klf15*(A) and the conservation of human and mouse (B). (TIF)
