# Supplementary material for: ARID1A Is Essential for Endometrial Function during Early Pregnancy
Source: PLoS Genet. 2015 Sep 17;11(9):e1005537. doi: 10.1371/journal.pgen.1005537 (PMC4574948; doi:10.1371/journal.pgen.1005537)
Supplement: S1 Table — (PDF) [file pgen.1005537.s001.pdf]

Supplemental Table 1. *Arid1a*$^{d/d}$ mice were sterile.

Genotype	Number of Mice Tested	Number of Litters	Number of Pups	Average Pups / Litter	Average Number of Litters /Mouse
Arid1a$^{f/f}$	9	52	375	7.21 ± 0.29	5.78 ± 0.15
Arid1a$^{d/d}$	9	0	0	0	0
