# Supplementary material for: ARID1A Is Essential for Endometrial Function during Early Pregnancy
Source: PLoS Genet. 2015 Sep 17;11(9):e1005537. doi: 10.1371/journal.pgen.1005537 (PMC4574948; doi:10.1371/journal.pgen.1005537)
Supplement: S2 Table — (PDF) [file pgen.1005537.s002.pdf]

Supplemental Table 2. *Arid1a*$^{d/d}$ has normal ovulation and fertilization.

Genotype	Total Eggs	Average Eggs/ Female
Arid1a$^{f/f}$ *(n=4)*	78	19.50 ± 1.85
Arid1a$^{d/d}$ *(n=7)*	139	19.86 ± 0.99

Genotype	Fertilized Eggs/ Total Eggs	Fertilization Rate
Arid1a$^{f/f}$ *(n=3)*	33 / 62	53.16 %
Arid1a$^{d/d}$ *(n=3)*	24 / 59	40.92 %
