# Supplementary material for: ARID1A Is Essential for Endometrial Function during Early Pregnancy
Source: PLoS Genet. 2015 Sep 17;11(9):e1005537. doi: 10.1371/journal.pgen.1005537 (PMC4574948; doi:10.1371/journal.pgen.1005537)
Supplement: S3 Table — (PDF) [file pgen.1005537.s003.pdf]

Supplemental Table 3. List of regulated genes by *Arid1a* ablation

Probe Set ID	Accession_ID	Gene	Description	Fold
1415938_at	NM_009258	Spink3	serine peptidase inhibitor, Kazal type 3	-89.74
1420282_s_at	NM_053260	Prss29	protease, serine, 29	-30.70
1449992_at	NM_053260	Prss29	protease, serine, 29	-25.54
1450802_at	NM_053259	Prss28	protease, serine, 28	-20.69
1455898_x_at	NM_011401	Slc2a3	solute carrier family 2 (facilitated glucose transporter), member 3	-12.16
1437052_s_at	NM_011401	Slc2a3	solute carrier family 2 (facilitated glucose transporter), member 3	-10.64
1454608_x_at	NM_013697	Ttr	transthyretin	-8.34
1423285_at	NM_001198835	Coch	coagulation factor C homolog (Limulus polyphemus)	-7.68
1420338_at	NM_009660	Alox15	arachidonate 15-lipoxygenase	-7.56
1452320_at	NM_001081088	Lrp2	low density lipoprotein receptor-related protein 2	-7.50
1426526_s_at	NM_007696	Ovgp1	oviductal glycoprotein 1	-7.27
1427133_s_at	NM_001081088	Lrp2	low density lipoprotein receptor-related protein 2	-7.20
1421404_at	NM_011339	Cxcl15	chemokine (C-X-C motif) ligand 15	-6.00
1420678_a_at	NM_019583	Il17rb	interleukin 17 receptor B	-5.91
1417094_at	NM_001146057	Acot7	acyl-CoA thioesterase 7	-5.89
1439543_at	XM_622231		RIKEN cDNA 1110064A23 gene	-5.70
1427514_at	XM_888832		hypothetical protein LOC624295	-5.56
1438377_x_at	NM_054055	Slc13a3	solute carrier family 13 (sodium-dependent dicarboxylate transporter), member 3	-5.04
1451502_at	NM_011987	Pla2g10	phospholipase A2, group X	-5.04
1426039_a_at	NM_145684	Alox12e	arachidonate lipoxygenase, epidermal	-4.98
1456335_at	NM_001033288	Gm106	predicted gene 106	-4.94
1418697_at	NM_009349	Inmt	indolethylamine N-methyltransferase	-4.94
1457429_s_at	NM_001033288	Gm106	predicted gene 106	-4.72
1416953_at	NM_010217	Ctgf	connective tissue growth factor	-4.68
1433529_at	NM_173749	Pamr1	peptidase domain containing associated with muscle regeneration 1	-4.64
1448891_at	NM_030707	Fcrls	Fc receptor-like S, scavenger receptor	-4.59
1419606_a_at	NM_011618	Tnnt1	troponin T1, skeletal, slow	-4.49
1427963_s_at	NM_153133	Rdh9	retinol dehydrogenase 9	-4.30
1416612_at	NM_009994	Cyp1b1	cytochrome P450, family 1, subfamily b, polypeptide 1	-4.29
1456428_at	NM_011339	Cxcl15	chemokine (C-X-C motif) ligand 15	-4.22
1448964_at	NM_009789	S100g	S100 calcium binding protein G	-4.18
1420940_x_at	NM_009063	Rgs5	regulator of G-protein signaling 5	-4.09
1418259_a_at	NM_009849	Entpd2	ectonucleoside triphosphate diphosphohydrolase 2	-4.06
1421134_at	NM_009704	Areg	amphiregulin	-3.97
1423100_at	NM_010234	Fos	FBJ osteosarcoma oncogene	-3.91
1420942_s_at	NM_009063	Rgs5	regulator of G-protein signaling 5	-3.89
1437361_at	NM_001105561		predicted gene 11545	-3.87
1427492_at	NM_181579	Pof1b	premature ovarian failure 1B	-3.83
1416560_at	NM_054055	Slc13a3	solute carrier family 13 (sodium-dependent dicarboxylate transporter), member 3	-3.83
1456084_x_at	NM_021355	Fmod	fibromodulin	-3.78
1436124_at	NM_177546	Pcyt1b	phosphate cytidylyltransferase 1, choline, beta isoform	-3.71
1459737_s_at	NM_013697	Ttr	transthyretin	-3.63
1455561_at	NM_177450	Cndp1	carnosine dipeptidase 1 (metallopeptidase M20 family)	-3.58
1416505_at	NM_010444	Nr4a1	nuclear receptor subfamily 4, group A, member 1	-3.57
1453801_at	NM_025416	Them5	thioesterase superfamily member 5	-3.56
1417290_at	NM_029796	Lrg1	leucine-rich alpha-2-glycoprotein 1	-3.53
1417065_at	NM_007913	Egr1	early growth response 1	-3.49
1418753_at	NM_013529	Gfpt2	glutamine fructose-6-phosphate transaminase 2	-3.48
1423858_a_at	NM_008256	Hmgcs2	3-hydroxy-3-methylglutaryl-Coenzyme A synthase 2	-3.46
1425417_x_at	NM_001101620	Klra8	killer cell lectin-like receptor, subfamily A, member 8	-3.44

Probe Set ID	Accession_ID	Gene	Description	Fold
1417466_at	NM_009063	Rgs5	regulator of G-protein signaling 5	-3.44
1428074_at	NM_001002267	Tmem158	transmembrane protein 158	-3.42
1435465_at	NM_029116	Kbtbd11	kelch repeat and BTB (POZ) domain containing 11	-3.39
1425436_x_at	NM_010648		killer cell lectin-like receptor, subfamily A, member 3 /// killer cell lectin-like receptor subfamily A, member 9	-3.34
1452652_at	NM_001002267	Tmem158	transmembrane protein 158	-3.34
1416321_s_at	NM_054077	Prelp	proline arginine-rich end leucine-rich repeat	-3.32
1438796_at	NM_015743	Nr4a3	nuclear receptor subfamily 4, group A, member 3	-3.31
1453003_at	NM_011436	Sorl1	sortilin-related receptor, LDLR class A repeats-containing	-3.28
1415939_at	NM_021355	Fmod	fibromodulin	-3.28
1448729_a_at	NM_011129		septin 4	-3.25
1460012_at	NM_027961	Wfdc3	WAP four-disulfide core domain 3	-3.24
1449279_at	NM_030677	Gpx2	glutathione peroxidase 2	-3.23
1418723_at	NM_022983	Lpar3	lysophosphatidic acid receptor 3	-3.22
1420941_at	NM_009063	Rgs5	regulator of G-protein signaling 5	-3.18
1429235_at	NM_030166	Galntl2	UDP-N-acetyl-alpha-D-galactosamine:polypeptide N-acetylgalactosaminyltransferase-like 2	-3.12
1424683_at	NM_001034851	Fam134b	family with sequence similarity 134, member B	-3.07
1448194_a_at	NR_001592	H19	H19 fetal liver mRNA	-3.06
1431833_a_at	NM_008256	Hmgcs2	3-hydroxy-3-methylglutaryl-Coenzyme A synthase 2	-3.04
1448949_at	NM_007607	Car4	carbonic anhydrase 4	-3.03
1458268_s_at	NM_008343	Igfbp3	insulin-like growth factor binding protein 3	-3.03
1426112_a_at	NM_001110320	Cd72	CD72 antigen	-2.98
1436332_at	NM_001012401	Hspb6	heat shock protein, alpha-crystallin-related, B6	-2.97
1426258_at	NM_011436	Sorl1	sortilin-related receptor, LDLR class A repeats-containing	-2.97
1450505_a_at	NM_001034851	Fam134b	family with sequence similarity 134, member B	-2.96
1418213_at	NM_033373	Krt23	keratin 23	-2.93
1451478_at	NM_001039554	Angptl7	angiopoietin-like 7	-2.92
1450976_at	NM_008681	Ndrg1	N-myc downstream regulated gene 1	-2.91
1433434_at	NM_178737	AW551984	expressed sequence AW551984	-2.91
1422975_at	NM_008604	Mme	membrane metallo endopeptidase	-2.90
1448021_at	NM_001142952	Fam46c	family with sequence similarity 46, member C	-2.90
1429236_at	NM_030166	Galntl2	UDP-N-acetyl-alpha-D-galactosamine:polypeptide N-acetylgalactosaminyltransferase-like 2	-2.88
1455610_at	NM_183312	Synm	synemin, intermediate filament protein	-2.88
1428909_at	NR_002860		RIKEN cDNA A130040M12 gene	-2.85
1433933_s_at	NM_175316	Slco2b1	solute carrier organic anion transporter family, member 2b1	-2.83
1420664_s_at	NM_011171	Procr	protein C receptor, endothelial	-2.82
1449091_at	NM_018778	Cldn8	claudin 8	-2.82
1450872_s_at	NM_001111100	Lipa	lysosomal acid lipase A	-2.82
1427455_x_at	BI107286		immunoglobulin kappa chain variable 28 (V28)	-2.81
1449106_at	NM_001083929	Gpx3	glutathione peroxidase 3	-2.81
1419060_at	NM_013542	Gzmb	granzyme B	-2.80
1418726_a_at	NM_001130174	Tnnt2	troponin T2, cardiac	-2.79
1418858_at	NM_023617	Aox3	aldehyde oxidase 3	-2.79
1435560_at	NM_008400	Itgal	integrin alpha L	-2.78
1429843_at	NM_001099296	Grrp1	glycine/arginine rich protein 1	-2.78
1438651_a_at	NM_011784	Aplnr	apelin receptor	-2.78
1437718_x_at	NM_021355	Fmod	fibromodulin	-2.76
1422637_at	NM_018750	Rassf5	Ras association (RalGDS/AF-6) domain family member 5	-2.76
1451204_at	NM_001168318	Scara5	scavenger receptor class A, member 5 (putative)	-2.76
1419411_at	NM_009312	Tac2	tachykinin 2	-2.75
1419759_at	NM_011076	Abcb1a	ATP-binding cassette, sub-family B (MDR/TAP), member 1A	-2.75
1455587_at	NM_182783	Fam167b	family with sequence similarity 167, member B	-2.74

Probe Set ID	Accession_ID	Gene	Description	Fold
1435386_at	NM_011708	Vwf	Von Willebrand factor homolog	-2.74
1447808_s_at	NM_001145899	Slc15a2	solute carrier family 15 (H+/peptide transporter), member 2	-2.74
1427521_a_at	NM_001164263		RIKEN cDNA 9930023K05 gene	-2.73
1427345_a_at	NM_133670	Sult1a1	sulfotransferase family 1A, phenol-preferring, member 1	-2.73
1425548_a_at	NM_010734	Lst1	leukocyte specific transcript 1	-2.72
1423062_at	NM_008343	Igfbp3	insulin-like growth factor binding protein 3	-2.70
1418674_at	NM_011019	Osmr	oncostatin M receptor	-2.70
1455050_at	NM_178791		RIKEN cDNA E130203B14 gene	-2.70
1433956_at	NM_009868	Cdh5	cadherin 5	-2.69
1424967_x_at	NM_001130174	Tnnt2	troponin T2, cardiac	-2.69
1460259_s_at	NM_009899	Clca1	chloride channel calcium activated 1	-2.69
1436739_at	NM_177322	Agtr1a	angiotensin II receptor, type 1a	-2.69
1460245_at	NM_010654	Klrd1	killer cell lectin-like receptor, subfamily D, member 1	-2.69
1419309_at	NM_010329	Pdpn	podoplanin	-2.66
1421653_a_at	NM_134051		immunoglobulin heavy chain 2 (serum IgA)	-2.65
1418204_s_at	NM_019467	Aif1	allograft inflammatory factor 1	-2.65
1427660_x_at	BC013496		immunoglobulin kappa chain variable 28 (V28)	-2.64
1424730_a_at	NM_001145899	Slc15a2	solute carrier family 15 (H+/peptide transporter), member 2	-2.64
1434647_at	NM_178748	Egflam	EGF-like, fibronectin type III and laminin G domains	-2.63
1420760_s_at	NM_008681	Ndrg1	N-myc downstream regulated gene 1	-2.63
1415996_at	NM_001009935	Txnip	thioredoxin interacting protein	-2.62
1450731_s_at	NM_178589	Tnfrsf21	tumor necrosis factor receptor superfamily, member 21	-2.61
1417481_at	NM_001168392	Ramp1	receptor (calcitonin) activity modifying protein 1	-2.60
1452308_a_at	NM_178405	Atp1a2	ATPase, Na+/K+ transporting, alpha 2 polypeptide	-2.60
1443823_s_at	NM_178405	Atp1a2	ATPase, Na+/K+ transporting, alpha 2 polypeptide	-2.60
1448323_a_at	NM_007542	Bgn	biglycan	-2.60
1438602_s_at	NM_008555	Masp1	mannan-binding lectin serine peptidase 1	-2.59
1425644_at	NM_001122899	Lepr	leptin receptor	-2.59
1419647_a_at	NM_133662	Ier3	immediate early response 3	-2.59
1434893_at	NM_178405	Atp1a2	ATPase, Na+/K+ transporting, alpha 2 polypeptide	-2.59
1455452_x_at	NM_001010826	Kctd14	potassium channel tetramerisation domain containing 14	-2.58
1448433_a_at	NM_008788	Pcolce	procollagen C-endopeptidase enhancer protein	-2.58
1425763_x_at	BC019425		immunoglobulin heavy chain 2 (serum IgA)	-2.57
1452514_a_at	NM_001122733	Kit	kit oncogene	-2.57
1437685_x_at	NM_021355	Fmod	fibromodulin	-2.56
1424613_at	NM_001195774	Gprc5b	G protein-coupled receptor, family C, group 5, member B	-2.56
1460632_at	NM_133832	Rdh10	retinol dehydrogenase 10 (all-trans)	-2.53
1423233_at	NM_007679	Cebpd	CCAAT/enhancer binding protein (C/EBP), delta	-2.52
1437123_at	NM_153127	Mmrn2	multimerin 2	-2.52
1427465_at	NM_178405	Atp1a2	ATPase, Na+/K+ transporting, alpha 2 polypeptide	-2.51
1455271_at	NM_001145034		predicted gene 13889	-2.51
1426633_s_at	NM_001010826	Kctd14	potassium channel tetramerisation domain containing 14	-2.50
1451161_a_at	NM_010130	Emr1	EGF-like module containing, mucin-like, hormone receptor-like sequence 1	-2.49
1460356_at	NM_027102	Esam	endothelial cell-specific adhesion molecule	-2.49
1456174_x_at	NM_008681	Ndrg1	N-myc downstream regulated gene 1	-2.48
1418059_at	NM_133222	Eltd1	EGF, latrophilin seven transmembrane domain containing 1	-2.47
1428332_at	NM_178149	Pik3ip1	phosphoinositide-3-kinase interacting protein 1	-2.47
1417852_x_at	NM_009899	Clca1	chloride channel calcium activated 1	-2.47
1424034_at	NM_013646	Rora	RAR-related orphan receptor alpha	-2.46
1449591_at	NM_007609	Casp4	caspase 4, apoptosis-related cysteine peptidase	-2.46
1455451_at	NM_001010826	Kctd14	potassium channel tetramerisation domain containing 14	-2.45
1421287_a_at	NM_001032378	Pecam1	platelet/endothelial cell adhesion molecule 1	-2.44

Probe Set ID	Accession_ID	Gene	Description	Fold
1425985_s_at	NM_008555	Masp1	mannan-binding lectin serine peptidase 1	-2.44
1424737_at	NM_009381	Thrsp	thyroid hormone responsive SPOT14 homolog (Rattus)	-2.43
1450627_at	NM_020332	Ank	progressive ankylosis	-2.43
1425603_at	NM_001098271		transmembrane protein 176A	-2.43
1443937_at	BE634648			-2.43
1428136_at	NM_013834	Sfrp1	secreted frizzled-related protein 1	-2.42
1432579_at	NM_025789	Rsph3a	radial spoke 3A homolog (Chlamydomonas)	-2.42
1437378_x_at	NM_016741	Scarb1	scavenger receptor class B, member 1	-2.42
1423037_at	NM_011784	Aplnr	apelin receptor	-2.42
1436941_at	NM_001134457	Fam55c	family with sequence similarity 55, member C	-2.41
1450276_a_at	NM_001146196	Scin	scinderin	-2.41
1424067_at	NM_010493	Icam1	intercellular adhesion molecule 1	-2.41
1418945_at	NM_010809	Mmp3	matrix metallopeptidase 3	-2.40
1422625_at	NM_001135688	Ly6h	lymphocyte antigen 6 complex, locus H	-2.40
1419569_a_at	NM_001113527	Isg20	interferon-stimulated protein	-2.40
1420994_at	NM_001159407	B3gnt5	UDP-GlcNAc:betaGal beta-1,3-N-acetylglucosaminyltransferase 5	-2.40
1452202_at	NM_001008548	Pde2a	phosphodiesterase 2A, cGMP-stimulated	-2.39
1459989_at	AV271189			-2.38
1428016_a_at	NM_028544	Rasip1	Ras interacting protein 1	-2.38
1455582_at	BI156044			-2.37
1420788_at	NM_016970	Klrg1	killer cell lectin-like receptor subfamily G, member 1	-2.37
1435361_at	NM_001025379	Sema3g	sema domain, immunoglobulin domain (Ig), short basic domain, secreted, (semaphorin) 3G	-2.36
1419272_at	NM_010851	Myd88	myeloid differentiation primary response gene 88	-2.36
1460218_at	NM_013706	Cd52	CD52 antigen	-2.36
1415856_at	NM_010330	Emb	embigin	-2.36
1429381_x_at	AK007826		immunoglobulin heavy chain 2 (serum IgA)	-2.35
1426171_x_at	NM_001110323	Klra7	killer cell lectin-like receptor, subfamily A, member 7	-2.35
1422632_at	NM_009985	Ctsw	cathepsin W	-2.35
1451411_at	NM_001195774	Gprc5b	G protein-coupled receptor, family C, group 5, member B	-2.35
1416411_at	NM_008183	Gstm2	glutathione S-transferase, mu 2	-2.34
1422638_s_at	NM_018750	Rassf5	Ras association (RalGDS/AF-6) domain family member 5	-2.34
1456156_at	NM_001122899	Lepr	leptin receptor	-2.34
1420484_a_at	NM_011707	Vtn	vitronectin	-2.34
1417620_at	NM_009008	Rac2	RAS-related C3 botulinum substrate 2	-2.33
1440911_at	NM_153393	Col23a1	collagen, type XXIII, alpha 1	-2.33
1428776_at	NM_029415	Slc10a6	solute carrier family 10 (sodium/bile acid cotransporter family), member 6	-2.33
1455961_at	AV174022			-2.33
1434202_a_at	NM_183187	Fam107a	family with sequence similarity 107, member A	-2.33
1423319_at	NM_008245	Hhex	hematopoietically expressed homeobox	-2.33
1440830_at	NM_001081178	Gpr116	G protein-coupled receptor 116	-2.32
1422973_a_at	NM_009381	Thrsp	thyroid hormone responsive SPOT14 homolog (Rattus)	-2.32
1450855_at	NM_033474	Arvcf	armadillo repeat gene deleted in velo-cardio-facial syndrome	-2.32
1455447_at	NR_015481		RIKEN cDNA D430019H16 gene	-2.32
1416405_at	NM_007542	Bgn	biglycan	-2.31
1418004_a_at	NM_001164207	Tmem176b	transmembrane protein 176B	-2.30
1415997_at	NM_001009935	Txnip	thioredoxin interacting protein	-2.30
1448562_at	NM_001159401	Upp1	uridine phosphorylase 1	-2.30
1448669_at	NM_015814	Dkk3	dickkopf homolog 3 (Xenopus laevis)	-2.29
1429761_at	NM_001007596	Rtn1	reticulon 1	-2.29
1418021_at	NM_009780	C4b	complement component 4B (Childo blood group)	-2.29
1447410_at	BB712583			-2.29

Probe Set ID	Accession_ID	Gene	Description	Fold
1434141_at	NM_021896	Gucy1a3	guanylate cyclase 1, soluble, alpha 3	-2.29
1434800_at	NM_001109753	Sv2b	synaptic vesicle glycoprotein 2 b	-2.29
1449131_s_at	NM_007639	Cd1d1	CD1d1 antigen	-2.29
1419836_at	AU040583			-2.29
1426640_s_at	NM_144551	Trib2	tribbles homolog 2 (Drosophila)	-2.28
1452400_a_at	NR_015348	Hoxa11as	HOXA11 antisense RNA (non-protein coding)	-2.28
1417590_at	NM_024264	Cyp27a1	cytochrome P450, family 27, subfamily a, polypeptide 1	-2.28
1448759_at	NM_008368	Il2rb	interleukin 2 receptor, beta chain	-2.28
1455913_x_at	NM_013697	Ttr	transthyretin	-2.28
1418340_at	NM_010185	Fcer1g	Fc receptor, IgE, high affinity I, gamma polypeptide	-2.28
1419758_at	NM_011076	Abcb1a	ATP-binding cassette, sub-family B (MDR/TAP), member 1A	-2.28
1437418_at	XR_105368	Gm3515	predicted gene 3515	-2.27
1427381_at	NM_008392	Irg1	immunoresponsive gene 1	-2.27
1455467_at	NM_001142781	Fam188b	family with sequence similarity 188, member B	-2.27
1428896_at	NM_026840	Pdgfrl	platelet-derived growth factor receptor-like	-2.27
1416121_at	NM_010728	Lox	lysyl oxidase	-2.25
1451031_at	NM_016687	Sfrp4	secreted frizzled-related protein 4	-2.25
1436515_at	NM_001109661	Bach2	BTB and CNC homology 2	-2.25
1453839_a_at	NM_023734	Pi16	peptidase inhibitor 16	-2.24
1416811_s_at	NM_001145799	Ctla2a	cytotoxic T lymphocyte-associated protein 2 alpha	-2.23
1449135_at	NM_009236	Sox18	SRY-box containing gene 18	-2.23
1442012_at	AV381755		expressed sequence AU015791	-2.23
1450769_s_at	NM_023377	Stard5	StAR-related lipid transfer (START) domain containing 5	-2.23
1426852_x_at	NM_010930	Nov	nephroblastoma overexpressed gene	-2.23
1426851_a_at	NM_010930	Nov	nephroblastoma overexpressed gene	-2.23
1418058_at	NM_133222	Eltd1	EGF, latrophilin seven transmembrane domain containing 1	-2.22
1418932_at	NM_017373	Nfil3	nuclear factor, interleukin 3, regulated	-2.22
1418788_at	NM_013690	Tek	endothelial-specific receptor tyrosine kinase	-2.22
1426632_at	NM_001010826	Kctd14	potassium channel tetramerisation domain containing 14	-2.22
1436713_s_at	NR_003633	Meg3	maternally expressed 3	-2.22
1428455_at	NM_181277	Col14a1	collagen, type XIV, alpha 1	-2.22
1437165_a_at	NM_008788	Pcolce	procollagen C-endopeptidase enhancer protein	-2.22
1416576_at	NM_007707	Socs3	suppressor of cytokine signaling 3	-2.22
1449360_at	NM_007781	Csf2rb2	colony stimulating factor 2 receptor, beta 2, low-affinity (granulocyte-macrophage)	-2.21
1447707_s_at	NM_001008548	Pde2a	phosphodiesterase 2A, cGMP-stimulated	-2.21
1437889_x_at	NM_007542	Bgn	biglycan	-2.21
1436544_at	NM_153389	Atp10d	ATPase, class V, type 10D	-2.21
1419442_at	NM_016762	Matn2	matrilin 2	-2.21
1426766_at	NM_134022		RIKEN cDNA 6330403K07 gene	-2.20
1454646_at	NM_146008	Tcp11l2	t-complex 11 (mouse) like 2	-2.20
1419476_at	NM_021475	Adamdec1	ADAM-like, decysin 1	-2.20
1434354_at	NM_172778	Maob	monoamine oxidase B	-2.19
1423585_at	NM_001159518	Igfbp7	insulin-like growth factor binding protein 7	-2.19
1436279_at	NM_145947	Slc26a7	solute carrier family 26, member 7	-2.19
1437324_x_at	NM_021355	Fmod	fibromodulin	-2.19
1422833_at	NM_010446	Foxa2	forkhead box A2	-2.18
1452417_x_at	AV057155		immunoglobulin kappa chain variable 28 (V28)	-2.18
1427298_at	NR_002870	Dnm3os	dynamin 3, opposite strand	-2.18
1455422_x_at	NM_011129		septin 4	-2.17
1424596_s_at	NM_144799	Lmcd1	LIM and cysteine-rich domains 1	-2.17
1428394_at	NM_172267	Phyhd1	phytanoyl-CoA dioxygenase domain containing 1	-2.17
1460412_at	NM_024237	Fbln7	fibulin 7	-2.17

Probe Set ID	Accession_ID	Gene	Description	Fold
1449466_at	NM_011606	Clec3b	C-type lectin domain family 3, member b	-2.17
1447623_s_at	NM_008858	Prkd1	protein kinase D1	-2.17
1433600_at	NM_007417	Adra2a	adrenergic receptor, alpha 2a	-2.17
1456014_s_at	NM_153795	Fermt3	fermitin family homolog 3 (Drosophila)	-2.16
1417234_at	NM_008606	Mmp11	matrix metallopeptidase 11	-2.16
1440343_at	NM_153587	Rps6ka5	ribosomal protein S6 kinase, polypeptide 5	-2.16
1417343_at	NM_022004	Fxyd6	FXYD domain-containing ion transport regulator 6	-2.16
1419435_at	NM_009676	Aox1	aldehyde oxidase 1	-2.16
1418094_s_at	NM_007607	Car4	carbonic anhydrase 4	-2.15
1449403_at	NM_001163748	Pde9a	phosphodiesterase 9A	-2.15
1448390_a_at	NM_001172424	Dhrs3	dehydrogenase/reductase (SDR family) member 3	-2.15
1435148_at	NM_013415	Atp1b2	ATPase, Na+/K+ transporting, beta 2 polypeptide	-2.15
1458324_x_at	BB208251			-2.15
1440355_at	NM_175429	Kctd12b	potassium channel tetramerisation domain containing 12b	-2.14
1427168_a_at	NM_181277	Col14a1	collagen, type XIV, alpha 1	-2.13
1436329_at	NM_018781	Egr3	early growth response 3	-2.13
1416211_a_at	NM_008973	Ptn	pleiotrophin	-2.13
1435026_at	NM_052994	Spock2	sparc/osteonectin, cwcv and kazal-like domains proteoglycan 2	-2.13
1428922_at	NM_025817		RIKEN cDNA 1200009O22 gene	-2.13
1454880_s_at	NM_138313	Bmf	BCL2 modifying factor	-2.13
1435040_at	NM_028679	Irak3	interleukin-1 receptor-associated kinase 3	-2.13
1454967_at	NM_029870		RIKEN cDNA A930001N09 gene	-2.12
1449065_at	NM_012006	Acot1	acyl-CoA thioesterase 1	-2.12
1419706_a_at	NM_031185	Akap12	A kinase (PRKA) anchor protein (gravin) 12	-2.11
1460444_at	NM_177231	Arrb1	arrestin, beta 1	-2.11
1423915_at	NM_177068	Olfml2b	olfactomedin-like 2B	-2.11
1425400_a_at	NM_019563	Cited4	Cbp/p300-interacting transactivator, with Glu/Asp-rich carboxy-terminal domain, 4	-2.11
1431213_a_at	XM_001477842	Gm3579	predicted gene 3579	-2.11
1419040_at	NM_001163472	Cyp2d22	cytochrome P450, family 2, subfamily d, polypeptide 22	-2.11
1457825_x_at	NM_001130458	Tcn2	transcobalamin 2	-2.10
1455251_at	NM_001033228	Itga1	integrin alpha 1	-2.10
1419589_at	NM_010740	Cd93	CD93 antigen	-2.10
1451978_at	NM_010729	Loxl1	lysyl oxidase-like 1	-2.10
1416050_a_at	NM_016741	Scarb1	scavenger receptor class B, member 1	-2.10
1427351_s_at	BB226392	Igh-6	immunoglobulin heavy chain 6 (heavy chain of IgM)	-2.10
1434140_at	NM_001159485	Mcf2l	mcf.2 transforming sequence-like	-2.09
1447725_at	BE948505		RIKEN cDNA C030034E14 gene	-2.09
1456768_a_at	NM_153127	Mmrn2	multimerin 2	-2.09
1452264_at	NM_153533	Tenc1	tensin like C1 domain-containing phosphatase	-2.09
1428651_at	NM_029436	Klhl24	kelch-like 24 (Drosophila)	-2.09
1449368_at	NM_001190451	Dcn	decorin	-2.09
1449528_at	NM_010216	Figf	c-fos induced growth factor	-2.09
1419833_s_at	NM_139206	Arap3	ArfGAP with RhoGAP domain, ankyrin repeat and PH domain 3	-2.08
1424305_at	NM_152839	Igj	immunoglobulin joining chain	-2.08
1453486_a_at	NM_020052	Scube2	signal peptide, CUB domain, EGF-like 2	-2.07
1421571_a_at	NM_001099217		lymphocyte antigen 6 complex, locus C1	-2.07
1460255_at	NM_033622	Tnfsf13b	tumor necrosis factor (ligand) superfamily, member 13b	-2.07
1426886_at	NM_001033242	Cln5	ceroid-lipofuscinosis, neuronal 5	-2.07
1428988_at	NM_029600	Abcc3	ATP-binding cassette, sub-family C (CFTR/MRP), member 3	-2.07
1455137_at	NM_175930	Rapgef5	Rap guanine nucleotide exchange factor (GEF) 5	-2.07
1455820_x_at	NM_016741	Scarb1	scavenger receptor class B, member 1	-2.07
1460163_at	NM_001135172	C1qtnf7	C1q and tumor necrosis factor related protein 7	-2.06

Probe Set ID	Accession_ID	Gene	Description	Fold
1431050_at	NM_153587	Rps6ka5	ribosomal protein S6 kinase, polypeptide 5	-2.06
1428460_at	NM_001111015	Syn2	synapsin II	-2.06
1459557_at	BM115255			-2.06
1417898_a_at	NM_010370	Gzma	granzyme A	-2.06
1453285_at	NM_025915	Tmem88	transmembrane protein 88	-2.06
1436196_at	BE691546		hypothetical protein C030046G05	-2.05
1415855_at	NM_013598	Kitl	kit ligand	-2.05
1415857_at	NM_010330	Emb	embigin	-2.05
1424824_at	NM_198014	Slain1	SLAIN motif family, member 1	-2.05
1422997_s_at	NM_012006		acyl-CoA thioesterase 1	-2.05
1433942_at	NM_001039546	Myo6	myosin VI	-2.05
1437967_at	NM_001025576	Ccdc141	coiled-coil domain containing 141	-2.05
1429022_at	NM_001025372	Adcyap1r1	adenylate cyclase activating polypeptide 1 receptor 1	-2.05
1429097_at	NM_177378	Rnf150	ring finger protein 150	-2.05
1454866_s_at	NM_172469	Clic6	chloride intracellular channel 6	-2.05
1447806_s_at	NM_019684	Srpk3	serine/arginine-rich protein specific kinase 3	-2.05
1416164_at	NM_011812	Fbln5	fibulin 5	-2.05
1447936_at	NR_030738		RIKEN cDNA 2410006H16 gene	-2.04
1456111_at	NM_172921	Fam55d	family with sequence similarity 55, member D	-2.04
1435459_at	NM_018881	Fmo2	flavin containing monooxygenase 2	-2.04
1419182_at	NM_022814	Svep1	sushi, von Willebrand factor type A, EGF and pentraxin domain containing 1	-2.04
1419132_at	NM_011905	Tlr2	toll-like receptor 2	-2.04
1426968_a_at	NM_133832	Rdh10	retinol dehydrogenase 10 (all-trans)	-2.04
1419315_at	NM_029612	Slamf9	SLAM family member 9	-2.03
1423140_at	NM_001111100	Lipa	lysosomal acid lipase A	-2.03
1433907_at	NM_001029838	Pknox2	Pbx/knotted 1 homeobox 2	-2.03
1441506_at	NM_001190451	Dcn	decorin	-2.03
1448862_at	NM_010494	Icam2	intercellular adhesion molecule 2	-2.03
1436501_at	NM_001005863	Mtus1	mitochondrial tumor suppressor 1	-2.03
1416255_at	NM_008120	Gja4	gap junction protein, alpha 4	-2.03
1417220_at	NM_010176	Fah	fumarylacetoacetate hydrolase	-2.02
1425582_a_at	NM_001163522	Emcn	endomucin	-2.02
1448737_at	NM_019634	Tspan7	tetraspanin 7	-2.02
1418186_at	NM_008185	Gstt1	glutathione S-transferase, theta 1	-2.01
1435547_at	NM_001122667	Mkl2	MKL/myocardin-like 2	-2.01
1431211_s_at	NM_025416	Them5	thioesterase superfamily member 5	-2.01
1426285_at	NM_008481	Lama2	laminin, alpha 2	-2.01
1458642_at	NM_153590	Klre1	killer cell lectin-like receptor family E member 1	-2.01
1435603_at	NM_172463	Sned1	sushi, nidogen and EGF-like domains 1	-2.01
1456532_at	NM_027924	Pdgfd	platelet-derived growth factor, D polypeptide	-2.01
1436312_at	NM_001025597	Ikzf1	IKAROS family zinc finger 1	-2.01
1435701_at	NM_144853	Cyyr1	cysteine and tyrosine-rich protein 1	-2.01
1450770_at	NM_026142		RIKEN cDNA 3632451O06 gene	-2.00
1455869_at	BG862223			-2.00
1450698_at	NM_010090	Dusp2	dual specificity phosphatase 2	-1.99
1449037_at	NM_001110850	Crem	cAMP responsive element modulator	-1.99
1457094_at	NM_172530	She	src homology 2 domain-containing transforming protein E	-1.99
1422622_at	NM_008713	Nos3	nitric oxide synthase 3, endothelial cell	-1.99
1421326_at	NM_007780	Csf2rb	colony stimulating factor 2 receptor, beta, low-affinity (granulocyte-macrophage)	-1.99
1426251_at	NM_153107	Cpz	carboxypeptidase Z	-1.99
1448823_at	NM_001012477	Cxcl12	chemokine (C-X-C motif) ligand 12	-1.98
1451428_x_at	NM_001164564	Egfl7	EGF-like domain 7	-1.98

Probe Set ID	Accession_ID	Gene	Description	Fold
1416101_a_at	NM_015786	Hist1h1c	histone cluster 1, H1c	-1.98
1455542_at	NR_027923		RIKEN cDNA C630043F03 gene	-1.98
1436555_at	NM_001044740	Slc7a2	solute carrier family 7 (cationic amino acid transporter, y+ system), member 2	-1.98
1452244_at	NM_027519		RIKEN cDNA 6330406I15 gene	-1.97
1449079_s_at	NM_018784	St3gal6	ST3 beta-galactoside alpha-2,3-sialyltransferase 6	-1.97
1418796_at	NM_009131	Clec11a	C-type lectin domain family 11, member a	-1.97
1437785_at	NM_175314	Adamts9	a disintegrin-like and metallopeptidase (reprolysin type) with thrombospondin type 1 motif, 9	-1.97
1415935_at	NM_022315	Smoc2	SPARC related modular calcium binding 2	-1.97
1448883_at	NM_011175	Lgmn	legumain	-1.97
1451453_at	NM_010019	Dapk2	death-associated protein kinase 2	-1.97
1455090_at	NM_011923	Angptl2	angiopoietin-like 2	-1.97
1449475_at	NM_138652	Atp12a	ATPase, H+/K+ transporting, nongastric, alpha polypeptide	-1.96
1437308_s_at	NM_010169	F2r	coagulation factor II (thrombin) receptor	-1.96
1429831_at	NM_031376	Pik3ap1	phosphoinositide-3-kinase adaptor protein 1	-1.96
1438954_x_at	NM_010216	Figf	c-fos induced growth factor	-1.96
1451332_at	NM_145492	Zfp521	zinc finger protein 521	-1.96
1418162_at	NM_021297	Tlr4	toll-like receptor 4	-1.96
1435385_at	NM_080455	Tshz2	teashirt zinc finger family member 2	-1.96
1429178_at	NM_001145937	Odz3	odd Oz/ten-m homolog 3 (Drosophila)	-1.96
1442077_at	BB197581		RIKEN cDNA 2310076G05 gene	-1.96
1424807_at	NM_010681	Lama4	laminin, alpha 4	-1.95
1426235_a_at	NM_008131	Glul	glutamate-ammonia ligase (glutamine synthetase)	-1.95
1423061_at	NM_033474	Arvcf	armadillo repeat gene deleted in velo-cardio-facial syndrome	-1.95
1438966_x_at	NM_021355	Fmod	fibromodulin	-1.95
1452191_at	NM_028243	Prcp	prolylcarboxypeptidase (angiotensinase C)	-1.95
1422952_at	NM_023893	Ng23	Ng23 protein	-1.95
1449078_at	NM_018784	St3gal6	ST3 beta-galactoside alpha-2,3-sialyltransferase 6	-1.95
1427891_at	NM_153175	Gimap6	GTPase, IMAP family member 6	-1.95
1433741_at	NM_007646	Cd38	CD38 antigen	-1.94
1457156_at	NM_146241	Trhde	TRH-degrading enzyme	-1.94
1454086_a_at	NM_001142335	Lmo2	LIM domain only 2	-1.94
1416203_at	NM_007472	Aqp1	aquaporin 1	-1.94
1450753_at	NM_024253	Nkg7	natural killer cell group 7 sequence	-1.93
1438672_at	NM_133167	Parvb	parvin, beta	-1.93
1427371_at	NM_153145	Abca8a	ATP-binding cassette, sub-family A (ABC1), member 8a	-1.93
1449805_at	NM_053260	Prss29	Protease, serine, 29	-1.93
1457141_at	BB229969			-1.93
1452352_at	NM_001145801	Ctla2b	cytotoxic T lymphocyte-associated protein 2 beta	-1.92
1455660_at	NM_007780	Csf2rb	colony stimulating factor 2 receptor, beta, low-affinity (granulocyte-macrophage)	-1.92
1436939_at	NM_178680	Unc45b	unc-45 homolog B (C. elegans)	-1.92
1420388_at	NM_008939	Prss12	protease, serine, 12 neurotrypsin (motopsin)	-1.92
1418483_a_at	NM_001145821	Ggta1	glycoprotein galactosyltransferase alpha 1, 3	-1.92
1455899_x_at	NM_007707	Socs3	suppressor of cytokine signaling 3	-1.92
1423844_s_at	NM_144855	Cbs	cystathionine beta-synthase	-1.92
1437360_at	NM_001105245	Pcdh19	protocadherin 19	-1.92
1427074_at	NM_153594	Pcmtd2	protein-L-isoaspartate (D-aspartate) O-methyltransferase domain containing 2	-1.92
1449169_at	NM_008216	Has2	hyaluronan synthase 2	-1.92
1423909_at	NM_001098271	Tmem176a	transmembrane protein 176A	-1.92
1418981_at	NM_009808	Casp12	caspase 12	-1.91
1444260_at	BF661746			-1.91

Probe Set ID	Accession_ID	Gene	Description	Fold
1426624_a_at	NM_025347	Ypel3	yippee-like 3 (Drosophila)	-1.91
1418098_at	NM_080435	Adcy4	adenylate cyclase 4	-1.91
1423467_at	NM_021718	Ms4a4b	membrane-spanning 4-domains, subfamily A, member 4B	-1.91
1435823_x_at	NM_001164564	Egfl7	EGF-like domain 7	-1.91
1456629_at	NM_030697	Kank3	KN motif and ankyrin repeat domains 3	-1.91
1435805_at	NM_001033223	Lin7a	lin-7 homolog A (C. elegans)	-1.90
1450798_at	NM_031176	Tnxb	tenascin XB	-1.90
1433939_at	NM_010678	Aff3	AF4/FMR2 family, member 3	-1.90
1437595_at	NM_183160		RIKEN cDNA E030010A14 gene	-1.90
1435559_at	NM_001039546	Myo6	myosin VI	-1.90
1429159_at	NM_172471	Itih5	inter-alpha (globulin) inhibitor H5	-1.90
1434939_at	NM_010426	Foxf1a	forkhead box F1a	-1.89
1452937_s_at	NM_025455	Ccdc28b	coiled coil domain containing 28B	-1.89
1456061_at	NM_001077410	Gimap8	GTPase, IMAP family member 8	-1.89
1424375_s_at	NM_174990	Gimap4	GTPase, IMAP family member 4	-1.89
1452905_at	NR_003633	Meg3	maternally expressed 3	-1.89
1431413_at	NM_001168392	Ramp1	receptor (calcitonin) activity modifying protein 1	-1.89
1449389_at	NM_011527	Tal1	T-cell acute lymphocytic leukemia 1	-1.89
1419358_at	NM_030889	Sorcs2	sortilin-related VPS10 domain containing receptor 2	-1.89
1448529_at	NM_009378	Thbd	thrombomodulin	-1.88
1451019_at	NM_019861	Ctsf	cathepsin F	-1.88
1426758_s_at	NR_003633	Meg3	maternally expressed 3	-1.88
1435060_at	NM_001038710	Tmod2	tropomodulin 2	-1.88
1450958_at	NM_008536	Tm4sf1	transmembrane 4 superfamily member 1	-1.88
1426206_at	NM_028783	Robo4	roundabout homolog 4 (Drosophila)	-1.88
1427038_at	NM_001002927	Penk	preproenkephalin	-1.88
1424265_at	NM_028749	Npl	N-acetylneuraminate pyruvate lyase	-1.87
1450663_at	NM_011581	Thbs2	thrombospondin 2	-1.87
1442025_a_at	AI467657			-1.87
1424763_at	NM_029338	Rsph9	radial spoke head 9 homolog (Chlamydomonas)	-1.87
1456046_at	NM_010740	Cd93	CD93 antigen	-1.87
1426734_at	NM_177632	Fam43a	family with sequence similarity 43, member A	-1.87
1419292_at	NM_001042615	Htra3	HtrA serine peptidase 3	-1.87
1429185_at	NM_001081295		RIKEN cDNA 4631416L12 gene	-1.87
1427240_at	NM_177030	Dock6	dedicator of cytokinesis 6	-1.87
1433455_at	NM_008507	Sh2b3	SH2B adaptor protein 3	-1.87
1423555_a_at	NM_133871	Ifi44	interferon-induced protein 44	-1.87
1450044_at	NM_008057	Fzd7	frizzled homolog 7 (Drosophila)	-1.86
1421037_at	NM_008719	Npas2	neuronal PAS domain protein 2	-1.86
1435511_at	NM_001111015	Syn2	synapsin II	-1.86
1450641_at	NM_011701	Vim	vimentin	-1.86
1428547_at	NM_011851	Nt5e	5' nucleotidase, ecto	-1.86
1428942_at	NM_008630	Mt2	metallothionein 2	-1.86
1434873_a_at	NM_153788	Acap1	ArfGAP with coiled-coil, ankyrin repeat and PH domains 1	-1.86
1434457_at	NM_013673	Sp100	nuclear antigen Sp100	-1.86
1449988_at	NM_008376	Gimap1	GTPase, IMAP family member 1	-1.86
1439096_at	NM_027442	Ddo	D-aspartate oxidase	-1.85
1427329_a_at	AI326478	Igh-6	immunoglobulin heavy chain 6 (heavy chain of IgM)	-1.85
1449852_a_at	NM_133838	Ehd4	EH-domain containing 4	-1.85
1420459_at	NM_133229	Ripply3	ripply3 homolog (zebrafish)	-1.85
1452330_a_at	NM_024263	Mxra8	matrix-remodelling associated 8	-1.85
1451069_at	NM_145478	Pim3	proviral integration site 3	-1.85
1421840_at	NM_013454	Abca1	ATP-binding cassette, sub-family A (ABC1), member 1	-1.85

Probe Set ID	Accession_ID	Gene	Description	Fold
1438684_at	NM_001004363	Nuak1	NUAK family, SNF1-like kinase, 1	-1.85
1455466_at	NM_001081342	Gpr133	G protein-coupled receptor 133	-1.85
1424289_at	NM_145950	Osgin2	oxidative stress induced growth inhibitor family member 2	-1.85
1436044_at	NM_009135	Scn7a	sodium channel, voltage-gated, type VII, alpha	-1.85
1450678_at	NM_008404	Itgb2	integrin beta 2	-1.85
1451500_at	NM_181418	Ushbp1	Usher syndrome 1C binding protein 1	-1.85
1428393_at	NM_153529	Nrn1	neuritin 1	-1.85
1423946_at	NM_145978	Pdlim2	PDZ and LIM domain 2	-1.85
1436324_at	XM_001004201	Stard9	START domain containing 9	-1.84
1439789_at	NM_007897	Ebf1	early B-cell factor 1	-1.84
1432517_a_at	NM_010924	Nnmt	nicotinamide N-methyltransferase	-1.84
1429987_at	NM_030728		RIKEN cDNA 9930013L23 gene	-1.84
1426743_at	NM_145220	Appl2	adaptor protein, phosphotyrosine interaction, PH domain and leucine zipper containing 2	-1.84
1416371_at	NM_007470	Apod	apolipoprotein D	-1.84
1438972_x_at	NR_030682		RIKEN cDNA 2810410L24 gene	-1.84
1449878_a_at	NM_133648	Slc12a6	solute carrier family 12, member 6	-1.84
1418207_at	NM_001173372	Fxyd4	FXYD domain-containing ion transport regulator 4	-1.84
1421006_at	NM_053185	Col4a6	collagen, type IV, alpha 6	-1.84
1423135_at	NM_009382	Thy1	thymus cell antigen 1, theta	-1.83
1422557_s_at	NM_013602	Mt1	metallothionein 1	-1.83
1452834_at	NM_001083810	Prr5l	proline rich 5 like	-1.83
1437181_at	NM_033602	Peli2	pellino 2	-1.83
1451611_at	NM_139269	Pla2g16	phospholipase A2, group XVI	-1.83
1418257_at	NM_011390	Slc12a7	solute carrier family 12, member 7	-1.83
1417801_a_at	NM_001163557	Ppfibp2	PTPRF interacting protein, binding protein 2 (liprin beta 2)	-1.83
1449146_at	NM_010929	Notch4	Notch gene homolog 4 (Drosophila)	-1.83
1437241_at	NM_178357	Klf11	Kruppel-like factor 11	-1.83
1434248_at	NM_008856	Prkch	protein kinase C, eta	-1.83
1432176_a_at	NM_001146348	Eng	endoglin	-1.82
1451604_a_at	NM_009612	Acvrl1	activin A receptor, type II-like 1	-1.82
1434188_at	NM_172838	Slc16a12	solute carrier family 16 (monocarboxylic acid transporters), member 12	-1.82
1419128_at	NM_021334	Itgax	integrin alpha X	-1.82
1429954_at	NM_153197	Clec4a3	C-type lectin domain family 4, member a3	-1.82
1416246_a_at	NM_009898	Coro1a	coronin, actin binding protein 1A	-1.82
1430302_at	NM_029861	Cnrip1	cannabinoid receptor interacting protein 1	-1.82
1422673_at	NM_008858	Prkd1	protein kinase D1	-1.82
1416069_at	NM_019703	Pfkp	phosphofructokinase, platelet	-1.81
1451336_at	NM_010706	Lgals4	lectin, galactose binding, soluble 4	-1.81
1453022_at	NM_026730	Gpihbp1	GPI-anchored HDL-binding protein 1	-1.81
1416111_at	NM_009856	Cd83	CD83 antigen	-1.81
1437197_at	NM_172752	Sorbs2	sorbin and SH3 domain containing 2	-1.81
1432566_at	NM_030106	Tmsb15a	thymosin beta 15a	-1.81
1422545_at	NM_009324	Tbx2	T-box 2	-1.81
1433762_at	NR_027923		RIKEN cDNA C630043F03 gene	-1.81
1439622_at	NM_178045	Rassf4	Ras association (RalGDS/AF-6) domain family member 4	-1.81
1449408_at	NM_023844	Jam2	junction adhesion molecule 2	-1.81
1440527_at	BI440542			-1.81
1426127_x_at	NM_053153	Klra18	killer cell lectin-like receptor, subfamily A, member 18	-1.81
1417148_at	NM_001146268	Pdgfrb	platelet derived growth factor receptor, beta polypeptide	-1.81
1416625_at	NM_009776	Serping1	serine (or cysteine) peptidase inhibitor, clade G, member 1	-1.80
1457321_at	XM_001474094		RIKEN cDNA D130037M23 gene	-1.80
1421899_a_at	NM_008209	Mr1	major histocompatibility complex, class I-related	-1.80

Probe Set ID	Accession_ID	Gene	Description	Fold
1436948_a_at	NM_172930	Fam70a	family with sequence similarity 70, member A	-1.80
1420617_at	NM_026252	Cpeb4	cytoplasmic polyadenylation element binding protein 4	-1.80
1454699_at	NM_001013370	Sesn1	sestrin 1	-1.80
1417185_at	NM_010738	Ly6a	lymphocyte antigen 6 complex, locus A	-1.80
1434411_at	NM_007730	Col12a1	collagen, type XII, alpha 1	-1.80
1423584_at	NM_001159518	Igfbp7	insulin-like growth factor binding protein 7	-1.80
1435265_at	XR_105720		hypothetical LOC100504698	-1.79
1454942_at	NM_022018	Fam129a	family with sequence similarity 129, member A	-1.79
1437661_at	NM_001033220	AU021092	expressed sequence AU021092	-1.79
1421007_at	NM_053185	Col4a6	collagen, type IV, alpha 6	-1.79
1416416_x_at	NM_010358	Gstm1	glutathione S-transferase, mu 1	-1.79
1433711_s_at	NM_001013370	Sesn1	sestrin 1	-1.79
1459903_at	NM_011352	Sema7a	sema domain, immunoglobulin domain (Ig), and GPI membrane anchor, (semaphorin) 7A	-1.79
1422710_a_at	NM_001163691	Cacna1h	calcium channel, voltage-dependent, T type, alpha 1H subunit	-1.79
1424010_at	NM_029568	Mfap4	microfibrillar-associated protein 4	-1.79
1456295_at	BB304874		RIKEN cDNA B230114P17 gene	-1.79
1441584_at	BB555654			-1.79
1427005_at	NM_152804	Plk2	polo-like kinase 2 (Drosophila)	-1.78
1433525_at	NM_010332	Ednra	endothelin receptor type A	-1.78
1460227_at	NM_001044384	Timp1	tissue inhibitor of metalloproteinase 1	-1.78
1427405_s_at	NM_001003955	Rab11fip5	RAB11 family interacting protein 5 (class I)	-1.78
1435435_at	NM_080285	Cttnbp2	cortactin binding protein 2	-1.78
1448293_at	NM_007897	Ebf1	early B-cell factor 1	-1.78
1439518_at	NM_153127	Mmrn2	multimerin 2	-1.78
1417066_at	NM_001163290	Adck3	aarF domain containing kinase 3	-1.78
1417312_at	NM_015814	Dkk3	dickkopf homolog 3 (Xenopus laevis)	-1.78
1435254_at	NM_172775	Plxnb1	plexin B1	-1.78
1420819_at	NM_001029841	Sla	src-like adaptor	-1.78
1438648_x_at	NM_026860	Gkn3	gastrokine 3	-1.78
1452595_at	NM_172845	Adamts4	a disintegrin-like and metallopeptidase (reprolysin type) with thrombospondin type 1 motif, 4	-1.78
1423890_x_at	NM_009721	Atp1b1	ATPase, Na+/K+ transporting, beta 1 polypeptide	-1.78
1460700_at	NM_011486	Stat3	signal transducer and activator of transcription 3	-1.78
1449461_at	NM_022020	Rbp7	retinol binding protein 7, cellular	-1.78
1435600_s_at	NM_145536		cDNA sequence BC020535	-1.78
1455269_a_at	NM_009898	Coro1a	coronin, actin binding protein 1A	-1.78
1416318_at	NM_025429	Serpinb1a	serine (or cysteine) peptidase inhibitor, clade B, member 1a	-1.77
1455398_at	NM_133897	Lrrc8c	leucine rich repeat containing 8 family, member C	-1.77
1457025_at	NM_001080710	Sdr16c6	short chain dehydrogenase/reductase family 16C, member 6	-1.77
1456144_at	NM_001081035	Nav3	neuron navigator 3	-1.77
1417933_at	NM_008344	Igfbp6	insulin-like growth factor binding protein 6	-1.77
1418478_at	NM_057173	Lmo1	LIM domain only 1	-1.77
1434997_at	NM_001168304	Cdk19	cyclin-dependent kinase 19	-1.77
1436367_at	NM_029928	Ptprb	protein tyrosine phosphatase, receptor type, B	-1.77
1425822_a_at	NM_008052	Dtx1	deltex 1 homolog (Drosophila)	-1.77
1441975_at	NM_019807	Acpp	acid phosphatase, prostate	-1.77
1451355_at	NM_139306	Acer2	alkaline ceramidase 2	-1.77
1415983_at	NM_008879	Lcp1	lymphocyte cytosolic protein 1	-1.77
1433902_at	NM_001008785	Kbtbd8	kelch repeat and BTB (POZ) domain containing 8	-1.77
1418163_at	NM_021297	Tlr4	toll-like receptor 4	-1.77
1416194_at	NM_007823	Cyp4b1	cytochrome P450, family 4, subfamily b, polypeptide 1	-1.77
1435935_at	NM_001081236		RIKEN cDNA 2410131K14 gene	-1.77
1450065_at	NM_001037723	Adcy7	adenylate cyclase 7	-1.77

Probe Set ID	Accession_ID	Gene	Description	Fold
1460011_at	NM_001177713	Cyp26b1	cytochrome P450, family 26, subfamily b, polypeptide 1	-1.77
1428479_at	NM_001164109	Nfatc1	nuclear factor of activated T-cells, cytoplasmic, calcineurin-dependent 1	-1.77
1434756_at	AV292354		hypothetical protein 5430421B17	-1.77
1449310_at	NM_008964	Ptger2	prostaglandin E receptor 2 (subtype EP2)	-1.77
1449297_at	NM_009808	Casp12	caspase 12	-1.77
1436448_a_at	NM_008969	Ptgs1	prostaglandin-endoperoxide synthase 1	-1.77
1451340_at	NM_001172205	Arid5a	AT rich interactive domain 5A (MRF1-like)	-1.77
1428426_s_at	NM_001013025	Tgfbrap1	transforming growth factor, beta receptor associated protein 1	-1.77
1449455_at	NM_001172117	Hck	hemopoietic cell kinase	-1.76
1460180_at	NM_010422	Hexb	hexosaminidase B	-1.76
1460578_at	NM_172731	Fgd5	FYVE, RhoGEF and PH domain containing 5	-1.76
1416136_at	NM_008610	Mmp2	matrix metallopeptidase 2	-1.76
1440244_at	NM_133659	Erg	avian erythroblastosis virus E-26 (v-ets) oncogene related	-1.76
1450429_at	NM_007603	Capn6	calpain 6	-1.76
1416488_at	NM_007635	Ccng2	cyclin G2	-1.76
1428750_at	NM_026772	Cdc42ep2	CDC42 effector protein (Rho GTPase binding) 2	-1.76
1427944_at	NM_181541	Caprin2	caprin family member 2	-1.76
1428662_a_at	NM_001159900	Hopx	HOP homeobox	-1.76
1419056_at	NM_001025364	Rtn2	reticulon 2 (Z-band associated protein)	-1.76
1448617_at	NM_007651	Cd53	CD53 antigen	-1.75
1421965_s_at	NM_008716	Notch3	Notch gene homolog 3 (Drosophila)	-1.75
1415949_at	NM_013494	Cpe	carboxypeptidase E	-1.75
1416926_at	NM_021897	Trp53inp1	transformation related protein 53 inducible nuclear protein 1	-1.75
1418758_a_at	NM_001163548	Cyth3	cytohesin 3	-1.75
1423718_at	NM_021299	Ak3	adenylate kinase 3	-1.75
1460302_at	NM_011580	Thbs1	thrombospondin 1	-1.75
1454824_s_at	NM_001005863	Mtus1	mitochondrial tumor suppressor 1	-1.75
1422906_at	NM_011920	Abcg2	ATP-binding cassette, sub-family G (WHITE), member 2	-1.75
1424699_at	NM_145574	Ccdc136	coiled-coil domain containing 136	-1.74
1428391_at	NM_144538	Rab3il1	RAB3A interacting protein (rabin3)-like 1	-1.74
1448681_at	NM_008358	Il15ra	interleukin 15 receptor, alpha chain	-1.74
1452183_a_at	NR_003633	Meg3	maternally expressed 3	-1.74
1455965_at	NM_172845	Adamts4	a disintegrin-like and metallopeptidase (reprolysin type) with thrombospondin type 1 motif, 4	-1.74
1432332_a_at	NM_033080	Nudt19	nudix (nucleoside diphosphate linked moiety X)-type motif 19	-1.74
1427007_at	NM_028773	Sash3	SAM and SH3 domain containing 3	-1.74
1448715_x_at	NM_009834		SH3-domain GRB2-like (endophilin) interacting protein 1	-1.74
1417291_at	NM_011609	Tnfrsf1a	tumor necrosis factor receptor superfamily, member 1a	-1.74
1436568_at	NM_023844	Jam2	junction adhesion molecule 2	-1.74
1423484_at	NM_031397	Bicc1	bicaudal C homolog 1 (Drosophila)	-1.74
1433780_at	NM_026666	Ubn1	ubinuclein 1	-1.74
1418109_at	NM_008179	Gspt2	G1 to S phase transition 2	-1.74
1460242_at	NM_010016	Cd55	CD55 antigen	-1.74
1426708_at	NM_133738	Antxr2	anthrax toxin receptor 2	-1.74
1417816_s_at	NM_012032	Serinc3	serine incorporator 3	-1.74
1435059_at	BM937495			-1.74
1437451_at	NM_001033141	Ecscr	endothelial cell-specific chemotaxis regulator	-1.74
1427056_at	NM_001024139	Adamts15	a disintegrin-like and metallopeptidase (reprolysin type) with thrombospondin type 1 motif, 15	-1.74
1449141_at	NM_001163256	Fblim1	filamin binding LIM protein 1	-1.74
1442033_at	NM_183175	C1qtnf9	C1q and tumor necrosis factor related protein 9	-1.74
1458933_at	NM_001039371	Slc22a15	solute carrier family 22 (organic anion/cation transporter), member 15	-1.74

Probe Set ID	Accession_ID	Gene	Description	Fold
1422124_a_at	NM_001111316	Ptprc	protein tyrosine phosphatase, receptor type, C	-1.73
1448688_at	NM_013723	Podxl	podocalyxin-like	-1.73
1438705_at	NM_001109873	Cbfa2t3	core-binding factor, runt domain, alpha subunit 2, translocated to, 3 (human)	-1.73
1419380_at	NM_033327	Zfp423	zinc finger protein 423	-1.73
1448321_at	NM_001146217	Smoc1	SPARC related modular calcium binding 1	-1.73
1429286_at	NM_026860	Gkn3	gastrokine 3	-1.73
1449130_at	NM_007639	Cd1d1	CD1d1 antigen	-1.73
1422562_at	NM_019662	Rrad	Ras-related associated with diabetes	-1.73
1449363_at	NM_007498	Atf3	activating transcription factor 3	-1.73
1424443_at	NM_145375	Tm6sf1	transmembrane 6 superfamily member 1	-1.73
1459679_s_at	NM_001161817	Myo1b	myosin IB	-1.73
1443870_at	NM_001033336	Abcc4	ATP-binding cassette, sub-family C (CFTR/MRP), member 4	-1.73
1441305_at	NM_001114386	Nedd4l	neural precursor cell expressed, developmentally down-regulated gene 4-like	-1.73
1419674_a_at	NM_007876	Dpep1	dipeptidase 1 (renal)	-1.73
1436819_at	NM_001177323		septin 6	-1.73
1433501_at	NM_177662	Ctso	cathepsin O	-1.73
1422822_at	NM_023377	Stard5	StAR-related lipid transfer (START) domain containing 5	-1.73
1449533_at	NM_026433	Tmem100	transmembrane protein 100	-1.73
1443906_at	NM_010016	Cd55	CD55 antigen	-1.73
1439380_x_at	NR_003633	Meg3	maternally expressed 3	-1.72
1445597_s_at	NM_139269	Pla2g16	phospholipase A2, group XVI	-1.72
1426063_a_at	NM_010276	Gem	GTP binding protein (gene overexpressed in skeletal muscle)	-1.72
1436346_at	NM_153098	Cd109	CD109 antigen	-1.72
1428452_at	NR_027984		RIKEN cDNA 2810025M15 gene	-1.72
1434115_at	NM_019707	Cdh13	cadherin 13	-1.72
1434501_at	NM_001005342	Ypel4	yippee-like 4 (Drosophila)	-1.72
1448557_at	NM_001143776	Fam13c	family with sequence similarity 13, member C	-1.72
1430520_at	NM_001033851	Cpne8	copine VIII	-1.72
1450852_s_at	NM_010169	F2r	coagulation factor II (thrombin) receptor	-1.72
1451691_at	NM_010332	Ednra	endothelin receptor type A	-1.72
1426510_at	NM_178653	Sccpdh	saccharopine dehydrogenase (putative)	-1.72
1418651_at	NM_026470	Spata6	spermatogenesis associated 6	-1.72
1416978_at	NM_010189	Fcgrt	Fc receptor, IgG, alpha chain transporter	-1.72
1451152_a_at	NM_009721	Atp1b1	ATPase, Na+/K+ transporting, beta 1 polypeptide	-1.72
1428334_at	NM_172416	Ostm1	osteopetrosis associated transmembrane protein 1	-1.72
1434474_at	NM_147219	Abca5	ATP-binding cassette, sub-family A (ABC1), member 5	-1.72
1442051_at	NM_178216	Hist2h3c1	histone cluster 2, H3c1	-1.72
1434069_at	NM_177782	Prex1	phosphatidylinositol-3,4,5-trisphosphate-dependent Rac exchange factor 1	-1.72
1439866_at	NM_001081335	Cul9	cullin 9	-1.72
1426937_at	NM_027519		RIKEN cDNA 6330406I15 gene	-1.71
1449314_at	NM_011766	Zfpm2	zinc finger protein, multitype 2	-1.71
1418983_at	NM_001005784	Inadl	InaD-like (Drosophila)	-1.71
1448117_at	NM_013598	Kitl	kit ligand	-1.71
1441972_at	BE949277		RIKEN cDNA 6230424C14 gene	-1.71
1427287_s_at	NM_010586	Itpr2	inositol 1,4,5-triphosphate receptor 2	-1.71
1434478_at	NM_001033432	Heca	headcase homolog (Drosophila)	-1.71
1436101_at	NM_178607	Rnf24	ring finger protein 24	-1.71
1417780_at	NM_026058	Lass4	LAG1 homolog, ceramide synthase 4	-1.71
1438532_at	NM_001024720	Hmcn1	hemicentin 1	-1.71
1439117_at	NM_001040682	Clmn	calmin	-1.71
1434033_at	NM_011599	Tle1	transducin-like enhancer of split 1, homolog of Drosophila E(spl)	-1.71

Probe Set ID	Accession_ID	Gene	Description	Fold
1431777_a_at	NM_026122	Hmgn3	high mobility group nucleosomal binding domain 3	-1.71
1416053_at	NM_008516	Lrrn1	leucine rich repeat protein 1, neuronal	-1.71
1426724_at	NM_028044	Cnn3	calponin 3, acidic	-1.71
1434283_at	NM_023598	Arid5b	AT rich interactive domain 5B (MRF1-like)	-1.71
1426221_at	NM_001145957	Vwa5a	von Willebrand factor A domain containing 5A	-1.71
1418417_at	NM_010827	Msc	musculin	-1.71
1454727_at	NM_178928	Afap1l1	actin filament associated protein 1-like 1	-1.71
1426368_at	NM_028724	Rin2	Ras and Rab interactor 2	-1.71
1418762_at	NM_010016	Cd55	CD55 antigen	-1.70
1418406_at	NM_008803	Pde8a	phosphodiesterase 8A	-1.70
1454613_at	NM_001136086	Dpysl3	dihydropyrimidinase-like 3	-1.70
1415776_at	NM_007437	Aldh3a2	aldehyde dehydrogenase family 3, subfamily A2	-1.70
1455316_x_at	XM_915804		cDNA sequence BC094435	-1.70
1448330_at	NM_010358	Gstm1	glutathione S-transferase, mu 1	-1.70
1424383_at	NM_145402	Tmem51	transmembrane protein 51	-1.70
1454708_at	NM_001103177	Ablim1	actin-binding LIM protein 1	-1.70
1448200_at	NM_001130458	Tcn2	transcobalamin 2	-1.70
1456220_at	NM_176959	Fbxl7	F-box and leucine-rich repeat protein 7	-1.70
1451353_at	NM_145375	Tm6sf1	transmembrane 6 superfamily member 1	-1.70
1434903_s_at	NM_133193	Il1rl2	interleukin 1 receptor-like 2	-1.70
1416759_at	NM_001164433	Mical1	microtubule associated monoxygenase, calponin and LIM domain containing 1	-1.70
1418181_at	NM_001166388	Ptp4a3	protein tyrosine phosphatase 4a3	-1.69
1449025_at	NM_010501	Ifit3	interferon-induced protein with tetratricopeptide repeats 3	-1.69
1448709_at	NM_001080819	Arid1a	AT rich interactive domain 1A (SWI-like)	-1.69
1449124_at	NM_016846	Rgl1	ral guanine nucleotide dissociation stimulator,-like 1	-1.69
1456934_at	NM_009788	Calb1	calbindin 1	-1.69
1435084_at	BB200607		RIKEN cDNA C730049O14 gene	-1.69
1460580_at	NM_018814	Pcnx	pecanex homolog (Drosophila)	-1.69
1441811_x_at	NM_001098271	Tmem176a	transmembrane protein 176A	-1.69
1418511_at	NM_019759	Dpt	dermatopontin	-1.69
1439827_at	NM_175501	Adamts12	a disintegrin-like and metallopeptidase (reprolysin type) with thrombospondin type 1 motif, 12	-1.69
1422013_at	NM_001170332	Clec4a2	C-type lectin domain family 4, member a2	-1.69
1417702_a_at	NM_080462	Hnmt	histamine N-methyltransferase	-1.69
1427054_s_at	NM_001014399	Abi3bp	ABI gene family, member 3 (NESH) binding protein	-1.69
1436029_at	NM_031397	Bicc1	bicaudal C homolog 1 (Drosophila)	-1.69
1437498_at	BB327336	Gm9971	predicted gene 9971	-1.69
1427996_at	NM_153513	BC028528	cDNA sequence BC028528	-1.69
1451229_at	NM_144919	Hdac11	histone deacetylase 11	-1.69
1430526_a_at	NM_011416	Smarca2	SWI/SNF related, matrix associated, actin dependent regulator of chromatin, subfamily a, member 2	-1.69
1449491_at	NM_130859	Card10	caspase recruitment domain family, member 10	-1.69
1428508_at	NM_194334	Tbc1d2b	TBC1 domain family, member 2B	-1.69
1436223_at	NM_177290	Itgb8	integrin beta 8	-1.69
1417785_at	NM_134102	Pla1a	phospholipase A1 member A	-1.68
1450816_at	NM_015810	Polg2	polymerase (DNA directed), gamma 2, accessory subunit	-1.68
1449027_at	NM_133955	Rhou	ras homolog gene family, member U	-1.68
1456307_s_at	NM_001037723	Adcy7	adenylate cyclase 7	-1.68
1417174_at	NM_025464	Tmem218	transmembrane protein 218	-1.68
1435943_at	NM_007876	Dpep1	dipeptidase 1 (renal)	-1.68
1456978_s_at	NM_027559		DNA segment, Chr 19, ERATO Doi 652, expressed	-1.68
1446014_at	AU067791			-1.68
1421217_a_at	NM_001159301	Lgals9	lectin, galactose binding, soluble 9	-1.68

Probe Set ID	Accession_ID	Gene	Description	Fold
1456890_at	NM_172689	Ddx58	DEAD (Asp-Glu-Ala-Asp) box polypeptide 58	-1.68
1429351_at	NM_029436	Klhl24	kelch-like 24 (Drosophila)	-1.68
1453070_at	NM_001013753	Pcdh17	protocadherin 17	-1.68
1424968_at	NM_197999	Ces2g	carboxylesterase 2G	-1.67
1423326_at	NM_009848	Entpd1	ectonucleoside triphosphate diphosphohydrolase 1	-1.67
1420362_a_at	NM_007546	Bik	BCL2-interacting killer	-1.67
1433787_at	NM_001037906	Nell1	NEL-like 1 (chicken)	-1.67
1424834_s_at	NM_010586	Itpr2	inositol 1,4,5-triphosphate receptor 2	-1.67
1452014_a_at	NM_001111274	Igf1	insulin-like growth factor 1	-1.67
1442026_at	AI467657			-1.67
1435902_at	NM_153136	Nudt18	nudix (nucleoside diphosphate linked moiety X)-type motif 18	-1.67
1434548_at	NM_012032	Serinc3	serine incorporator 3	-1.67
1448559_at	NM_008027	Flot1	flotillin 1	-1.67
1417277_at	NM_024442	Cyp4f16	cytochrome P450, family 4, subfamily f, polypeptide 16	-1.67
1436755_at	NM_172471	Itih5	inter-alpha (globulin) inhibitor H5	-1.67
1435880_at	NM_001167883	Ankrd50	ankyrin repeat domain 50	-1.67
1436520_at	XM_003085583	Ahnak2	AHNAK nucleoprotein 2	-1.67
1426236_a_at	NM_008131	Glul	glutamate-ammonia ligase (glutamine synthetase)	-1.66
1439537_at	BG071024			-1.66
1435911_s_at	NM_178934	Slc2a12	solute carrier family 2 (facilitated glucose transporter), member12	-1.66
1451475_at	NM_026376	Plxnd1	plexin D1	-1.66
1433632_at	NM_001164598	Irf2bp2	interferon regulatory factor 2 binding protein 2	-1.66
1423450_a_at	NM_010474	Hs3st1	heparan sulfate (glucosamine) 3-O-sulfotransferase 1	-1.66
1436171_at	NM_001005508	Arhgap30	Rho GTPase activating protein 30	-1.66
1422645_at	NM_010424	Hfe	hemochromatosis	-1.66
1428284_at	NM_001001986		RIKEN cDNA 8430427H17 gene	-1.66
1416195_at	NM_008916	Inpp5k	inositol polyphosphate 5-phosphatase K	-1.66
1435195_at	NM_177354	Vash1	vasohibin 1	-1.66
1439925_at	BQ031166			-1.66
1452657_at	NM_026887	Ap1s2	adaptor-related protein complex 1, sigma 2 subunit	-1.66
1416766_at	NM_133684	Mosc2	MOCO sulphurase C-terminal domain containing 2	-1.66
1451782_a_at	NM_022880	Slc29a1	solute carrier family 29 (nucleoside transporters), member 1	-1.66
1458659_at	NM_207229		predicted gene 10393 /// placenta specific 9	-1.66
1433745_at	NM_001081302	Trio	triple functional domain (PTPRF interacting)	-1.66
1443250_at	NM_009061	Rgs2	regulator of G-protein signaling 2	-1.66
1434931_at	NM_001042752	Neo1	neogenin	-1.66
1434581_at	NM_026629		RIKEN cDNA 2410066E13 gene	-1.66
1448118_a_at	NM_009983	Ctsd	cathepsin D	-1.66
1435771_at	NM_013829	Plcb4	phospholipase C, beta 4	-1.66
1452952_at	NM_001081289		RIKEN cDNA 9030418K01 gene	-1.65
1425646_at	NM_145497		cDNA sequence BC016495	-1.65
1438704_at	NM_001039048	Trim63	tripartite motif-containing 63	-1.65
1425594_at	NM_011836	Lamc3	laminin gamma 3	-1.65
1452296_at	NM_011412	Slit3	slit homolog 3 (Drosophila)	-1.65
1426818_at	NM_001042592	Arrdc4	arrestin domain containing 4	-1.65
1455164_at	NM_020260	Arhgap31	Rho GTPase activating protein 31	-1.65
1457038_at	NM_172862	Frem2	Fras1 related extracellular matrix protein 2	-1.65
1423717_at	NM_021299	Ak3	adenylate kinase 3	-1.65
1426971_at	NM_023738	Uba7	ubiquitin-like modifier activating enzyme 7	-1.65
1435221_at	NM_001197321	Foxp1	forkhead box P1	-1.65
1456603_at	NM_029658	Fam101b	family with sequence similarity 101, member B	-1.65
1460367_at	NM_153198	Hbp1	high mobility group box transcription factor 1	-1.65
1438886_at	NM_013905	Heyl	hairy/enhancer-of-split related with YRPW motif-like	-1.65

Probe Set ID	Accession_ID	Gene	Description	Fold
1416302_at	NM_007897	Ebf1	early B-cell factor 1	-1.65
1416238_at	NM_011587	Tie1	tyrosine kinase with immunoglobulin-like and EGF-like domains 1	-1.65
1434856_at	NM_001081433	Ankrd44	ankyrin repeat domain 44	-1.64
1448525_a_at	NM_009761	Bnip3l	BCL2/adenovirus E1B interacting protein 3-like	-1.64
1434067_at	NR_015519	AI662270	expressed sequence AI662270	-1.64
1420919_at	NM_001037759	Sgk3	serum/glucocorticoid regulated kinase 3	-1.64
1448754_at	NM_011254	Rbp1	retinol binding protein 1, cellular	-1.64
1425678_a_at	NM_001164572	Snrk	SNF related kinase	-1.64
1436913_at	NM_001080818	Cdc14a	CDC14 cell division cycle 14 homolog A (S. cerevisiae)	-1.64
1442698_at	BB252728			-1.64
1428717_at	NM_027268	Scrn1	secernin 1	-1.64
1457170_at	NM_001033371	Lrrc36	leucine rich repeat containing 36	-1.64
1438756_at	NM_001190371	Ankrd29	ankyrin repeat domain 29	-1.64
1449022_at	NM_016701	Nes	nestin	-1.64
1426238_at	NM_009755	Bmp1	bone morphogenetic protein 1	-1.64
1425475_at	NM_001163155	Col4a5	collagen, type IV, alpha 5	-1.64
1434500_at	NM_053273	Ttyh2	tweety homolog 2 (Drosophila)	-1.64
1448201_at	NM_009144	Sfrp2	secreted frizzled-related protein 2	-1.64
1457052_at	NM_001081134	Kcng1	potassium voltage-gated channel, subfamily G, member 1	-1.64
1450792_at	NM_011662	Tyrobp	TYRO protein tyrosine kinase binding protein	-1.64
1451174_at	NM_146069	Lrrc33	leucine rich repeat containing 33	-1.64
1423805_at	NM_001008702	Dab2	disabled homolog 2 (Drosophila)	-1.64
1423586_at	NM_001190974	Axl	AXL receptor tyrosine kinase	-1.64
1418400_at	NM_026235	Larp6	La ribonucleoprotein domain family, member 6	-1.64
1435668_at	NM_024203	Fam120b	family with sequence similarity 120, member B	-1.64
1430522_a_at	NM_001080742	Vamp5	vesicle-associated membrane protein 5	-1.63
1435387_at	NM_001033633	Slc2a13	solute carrier family 2 (facilitated glucose transporter), member13	-1.63
1416708_a_at	NM_027898	Gramd1a	GRAM domain containing 1A	-1.63
1428211_at	NM_028944	P4htm	prolyl 4-hydroxylase, transmembrane (endoplasmic reticulum)	-1.63
1448839_at	NM_030697	Kank3	KN motif and ankyrin repeat domains 3	-1.63
1437113_s_at	NM_001164056	Pld1	phospholipase D1	-1.63
1427301_at	NM_007649	Cd48	CD48 antigen	-1.63
1456212_x_at	NM_007707	Socs3	suppressor of cytokine signaling 3	-1.63
1433512_at	NM_008026	Fli1	Friend leukemia integration 1	-1.63
1417601_at	NM_015811	Rgs1	regulator of G-protein signaling 1	-1.63
1429181_at	NM_001081275		RIKEN cDNA 1700009P17 gene	-1.63
1421335_a_at	NM_001164564	Egfl7	EGF-like domain 7	-1.63
1416554_at	NM_016861	Pdlim1	PDZ and LIM domain 1 (elfin)	-1.63
1452418_at	BF719154		RIKEN cDNA 1200016E24 gene	-1.63
1437584_at	BE685667			-1.63
1449514_at	NM_018869	Grk5	G protein-coupled receptor kinase 5	-1.63
1448864_at	NM_001164572	Snrk	SNF related kinase	-1.63
1451139_at	NM_028064	Slc39a4	solute carrier family 39 (zinc transporter), member 4	-1.63
1434010_at	NM_001037725	Fam117b	family with sequence similarity 117, member B	-1.63
1420295_x_at	NM_016691	Clcn5	chloride channel 5	-1.63
1451758_at	NM_011836	Lamc3	laminin gamma 3	-1.63
1418322_at	NM_001110850	Crem	cAMP responsive element modulator	-1.63
1416895_at	NM_001162425	Efna1	ephrin A1	-1.62
1451121_a_at	NM_133831	Gltscr2	glioma tumor suppressor candidate region gene 2	-1.62
1437100_x_at	NM_145478	Pim3	proviral integration site 3	-1.62
1435343_at	NM_175291	Dock10	dedicator of cytokinesis 10	-1.62
1454048_a_at	NM_027627		RIKEN cDNA 4931408A02 gene	-1.62
1459348_at	BB454472			-1.62

Probe Set ID	Accession_ID	Gene	Description	Fold
1424549_at	NM_001171002	Degs2	degenerative spermatocyte homolog 2 (Drosophila), lipid desaturase	-1.62
1439787_at	NM_001038839	P2rx7	purinergic receptor P2X, ligand-gated ion channel, 7	-1.62
1420872_at	NM_001161796	Gucy1b3	guanylate cyclase 1, soluble, beta 3	-1.62
1422079_at	NM_008856	Prkch	protein kinase C, eta	-1.62
1428375_at	NR_015524		RIKEN cDNA 4932415G12 gene	-1.62
1423994_at	NM_008441	Kif1b	kinesin family member 1B	-1.62
1428769_at	NM_001163421	Tatdn3	TatD DNase domain containing 3	-1.62
1428954_at	NM_023055	Slc9a3r2	solute carrier family 9 (sodium/hydrogen exchanger), member 3 regulator 2	-1.62
1416125_at	NM_010220	Fkbp5	FK506 binding protein 5	-1.62
1455010_at	NM_001081005		RIKEN cDNA 1500012F01 gene	-1.62
1437364_at	NM_172687	Coq3	coenzyme Q3 homolog, methyltransferase (yeast)	-1.62
1435348_at	BQ177188		RIKEN cDNA D930009K15 gene	-1.62
1422479_at	NM_019811	Acss2	acyl-CoA synthetase short-chain family member 2	-1.62
1428083_at	NR_003513	Neat1	nuclear paraspeckle assembly transcript 1 (non-protein coding)	-1.62
1435172_at	NM_001164789	Eomes	eomesodermin homolog (Xenopus laevis)	-1.62
1452666_a_at	NM_178874	Tmcc2	transmembrane and coiled-coil domains 2	-1.62
1423893_x_at	NM_009685	Apbb1	amyloid beta (A4) precursor protein-binding, family B, member 1	-1.62
1448538_a_at	NM_023665	D4Wsu53e	DNA segment, Chr 4, Wayne State University 53, expressed	-1.62
1425140_at	NM_145381	Lactb2	lactamase, beta 2	-1.62
1426587_a_at	NM_011486	Stat3	signal transducer and activator of transcription 3	-1.61
1439364_a_at	NM_008610	Mmp2	matrix metallopeptidase 2	-1.61
1458969_at	BG069506		expressed sequence AU019559	-1.61
1420558_at	NM_011347	Selp	selectin, platelet	-1.61
1448181_at	NM_023184	Klf15	Kruppel-like factor 15	-1.61
1454748_at	NM_172607	Naprt1	nicotinate phosphoribosyltransferase domain containing 1	-1.61
1429030_at	NM_001135172	C1qtnf7	C1q and tumor necrosis factor related protein 7	-1.61
1435342_at	NM_001033525	Kcnk6	potassium inwardly-rectifying channel, subfamily K, member 6	-1.61
1425396_a_at	NM_001162432	Lck	lymphocyte protein tyrosine kinase	-1.61
1433801_at	NM_001004155		RIKEN cDNA 9930012K11 gene	-1.61
1423695_at	NM_145537	Edem2	ER degradation enhancer, mannosidase alpha-like 2	-1.61
1435144_at	BM243379			-1.61
1418471_at	NM_008827	Pgf	placental growth factor	-1.61
1436043_at	NM_009135	Scn7a	sodium channel, voltage-gated, type VII, alpha	-1.61
1425109_at	NM_145394	Slc44a3	solute carrier family 44, member 3	-1.61
1446812_at	XM_001474844	Gm2818	Predicted gene 2818	-1.61
1454711_at	NM_001081302	Trio	triple functional domain (PTPRF interacting)	-1.61
1434413_at	NM_001111274	Igf1	insulin-like growth factor 1	-1.61
1416432_at	NM_001177752	Pfkfb3	6-phosphofructo-2-kinase/fructose-2,6-biphosphatase 3	-1.61
1435748_at	NM_010266	Gda	guanine deaminase	-1.61
1435256_at	NM_001081114	Clip3	CAP-GLY domain containing linker protein 3	-1.61
1424186_at	NM_026439	Ccdc80	coiled-coil domain containing 80	-1.61
1452382_at	NR_002870	Dnm3os	dynamin 3, opposite strand	-1.61
1429144_at	NM_001042671	Gpcpd1	glycerophosphocholine phosphodiesterase GDE1 homolog (S. cerevisiae)	-1.61
1434216_a_at	NM_033080	Nudt19	nudix (nucleoside diphosphate linked moiety X)-type motif 19	-1.61
1434360_s_at	NM_008981	Ptprg	protein tyrosine phosphatase, receptor type, G	-1.61
1417273_at	NM_013743	Pdk4	pyruvate dehydrogenase kinase, isoenzyme 4	-1.61
1431335_a_at	NM_023395	Wfdc1	WAP four-disulfide core domain 1	-1.61
1452985_at	NM_028283	Uaca	uveal autoantigen with coiled-coil domains and ankyrin repeats	-1.61
1427053_at	NM_001014399	Abi3bp	ABI gene family, member 3 (NESH) binding protein	-1.61
1436562_at	NM_172689	Ddx58	DEAD (Asp-Glu-Ala-Asp) box polypeptide 58	-1.60
1433993_at	NM_172741		RIKEN cDNA 4931406P16 gene	-1.60

Probe Set ID	Accession_ID	Gene	Description	Fold
1435941_at	NM_139228	Rhbdl3	rhomboid, veinlet-like 3 (Drosophila)	-1.60
1422474_at	NM_001177980	Pde4b	phosphodiesterase 4B, cAMP specific	-1.60
1456640_at	NM_001146299	Sh3rf2	SH3 domain containing ring finger 2	-1.60
1416007_at	NM_001163630	Satb1	special AT-rich sequence binding protein 1	-1.60
1440339_at	NM_008813	Enpp1	ectonucleotide pyrophosphatase/phosphodiesterase 1	-1.60
1455333_at	NM_001083587	Tns3	tensin 3	-1.60
1452832_s_at	NM_138651	Cds2	CDP-diacylglycerol synthase (phosphatidate cytidylyltransferase) 2	-1.60
1421917_at	NM_001083316	Pdgfra	platelet derived growth factor receptor, alpha polypeptide	-1.60
1436650_at	NM_001081243	Filip1	filamin A interacting protein 1	-1.60
1419470_at	NM_013531	Gnb4	guanine nucleotide binding protein (G protein), beta 4	-1.60
1424988_at	NM_153789	Mylip	myosin regulatory light chain interacting protein	-1.60
1423689_a_at	NM_153410	Gpsm1	G-protein signalling modulator 1 (AGS3-like, C. elegans)	-1.60
1424123_at	NM_145447	Mfsd7c	major facilitator superfamily domain containing 7C	-1.60
1457139_at	NM_177047	Auts2	autism susceptibility candidate 2	-1.60
1417862_at	NM_021427	Fam181b	family with sequence similarity 181, member B	-1.60
1429893_at	NM_134437	Il17rd	interleukin 17 receptor D	-1.60
1451427_a_at	NM_001164564	Egfl7	EGF-like domain 7	-1.60
1452351_at	NM_001145978	Parp4	poly (ADP-ribose) polymerase family, member 4	-1.60
1454685_at	NM_001038703	Gpr146	G protein-coupled receptor 146	-1.60
1436098_at	NM_009738	Bche	butyrylcholinesterase	-1.60
1455741_a_at	NM_199307	Ece1	endothelin converting enzyme 1	-1.60
1424208_at	NM_001136079	Ptger4	prostaglandin E receptor 4 (subtype EP4)	-1.60
1448409_at	NM_008511	Lrmp	lymphoid-restricted membrane protein	-1.60
1422571_at	NM_011581	Thbs2	thrombospondin 2	-1.60
1415975_at	NM_025821	Carhsp1	calcium regulated heat stable protein 1	-1.60
1439632_at	NM_013531	Gnb4	guanine nucleotide binding protein (G protein), beta 4	-1.59
1435133_at	NM_011673	Ugcg	UDP-glucose ceramide glucosyltransferase	-1.59
1455145_at	NM_001105245	Pcdh19	protocadherin 19	-1.59
1444734_at	BB183877		RIKEN cDNA A330001L22 gene	-1.59
1454974_at	NM_008744	Ntn1	netrin 1	-1.59
1421141_a_at	NM_001197321	Foxp1	forkhead box P1	-1.59
1416008_at	NM_001163630	Satb1	special AT-rich sequence binding protein 1	-1.59
1421964_at	NM_008716	Notch3	Notch gene homolog 3 (Drosophila)	-1.59
1423551_at	NM_019707	Cdh13	cadherin 13	-1.59
1440849_at	NM_009027	Rasgrf2	RAS protein-specific guanine nucleotide-releasing factor 2	-1.59
1417676_a_at	NM_001164401	Ptpro	protein tyrosine phosphatase, receptor type, O	-1.59
1460596_at	NM_009642	Agtrap	angiotensin II, type I receptor-associated protein	-1.59
1452106_at	NM_001029836	Npnt	nephronectin	-1.59
1455274_at	BI076807	Gm14226	predicted gene 14226	-1.59
1419572_a_at	NM_008992	Abcd4	ATP-binding cassette, sub-family D (ALD), member 4	-1.59
1436178_at	NM_173379	Leprel1	leprecan-like 1	-1.59
1423753_at	NM_026505	Bambi	BMP and activin membrane-bound inhibitor, homolog (Xenopus laevis)	-1.59
1426100_a_at	NM_021028	Tk2	thymidine kinase 2, mitochondrial	-1.59
1422697_s_at	NM_021878	Jarid2	jumonji, AT rich interactive domain 2	-1.59
1419412_at	NM_008510	Xcl1	chemokine (C motif) ligand 1	-1.59
1421824_at	NM_001145947	Bace1	beta-site APP cleaving enzyme 1	-1.59
1439604_at	NM_172053	Adamts16	a disintegrin-like and metallopeptidase (reprolysin type) with thrombospondin type 1 motif, 16	-1.59
1453173_at	NM_172398	Akr1b10	aldo-keto reductase family 1, member B10 (aldose reductase)	-1.59
1437874_s_at	NM_010422	Hexb	hexosaminidase B	-1.59
1430352_at	NM_175314	Adamts9	a disintegrin-like and metallopeptidase (reprolysin type) with thrombospondin type 1 motif, 9	-1.59

Probe Set ID	Accession_ID	Gene	Description	Fold
1439847_s_at	NM_010636	Klf12	Kruppel-like factor 12	-1.59
1434334_at	NM_178900	Prkd2	protein kinase D2	-1.59
1442187_at	NM_009747	Bdkrb2	Bradykinin receptor, beta 2	-1.58
1454777_at	NM_175316	Slco2b1	solute carrier organic anion transporter family, member 2b1	-1.58
1417501_at	NM_001163704	Fbxo6	F-box protein 6	-1.58
1424359_at	NM_153122	Oplah	5-oxoprolinase (ATP-hydrolysing)	-1.58
1435436_at	NM_010137	Epas1	endothelial PAS domain protein 1	-1.58
1422821_s_at	NM_023377	Stard5	StAR-related lipid transfer (START) domain containing 5	-1.58
1452474_a_at	NM_181728	Art3	ADP-ribosyltransferase 3	-1.58
1452792_at	NM_025943	Dzip1	DAZ interacting protein 1	-1.58
1426348_at	NM_009931	Col4a1	collagen, type IV, alpha 1	-1.58
1429543_at	AI553459		RIKEN cDNA 6230424C14 gene	-1.58
1459840_s_at	NM_025455	Ccdc28b	coiled coil domain containing 28B	-1.58
1440116_at	NM_194061		RIKEN cDNA D630045J12 gene	-1.58
1417477_at	NM_025294	Gm16515	predicted gene, Gm16515	-1.58
1427943_at	NM_029344	Acyp2	acylphosphatase 2, muscle type	-1.58
1454766_at	NM_001113424	Amn1	antagonist of mitotic exit network 1 homolog (S. cerevisiae)	-1.58
1451939_a_at	NM_016911	Srpx	sushi-repeat-containing protein	-1.58
1421074_at	NM_007825	Cyp7b1	cytochrome P450, family 7, subfamily b, polypeptide 1	-1.58
1416511_a_at	NM_001163346	Cdc42ep4	CDC42 effector protein (Rho GTPase binding) 4	-1.58
1436425_at	NM_172872	Kank4	KN motif and ankyrin repeat domains 4	-1.58
1415758_at	NM_028194	Fryl	furry homolog-like (Drosophila)	-1.58
1423570_at	NM_009593	Abcg1	ATP-binding cassette, sub-family G (WHITE), member 1	-1.58
1448830_at	NM_013642	Dusp1	dual specificity phosphatase 1	-1.58
1438953_at	NM_010216	Figf	c-fos induced growth factor	-1.58
1425788_a_at	NM_026728	Echdc2	enoyl Coenzyme A hydratase domain containing 2	-1.58
1423613_at	NM_080558	Ssfa2	sperm specific antigen 2	-1.58
1447830_s_at	NM_009061	Rgs2	regulator of G-protein signaling 2	-1.57
1447849_s_at	NM_001025577	Maf	avian musculoaponeurotic fibrosarcoma (v-maf) AS42 oncogene homolog	-1.57
1437401_at	NM_001111274	Igf1	insulin-like growth factor 1	-1.57
1451036_at	NM_138584	Spg21	spastic paraplegia 21 homolog (human)	-1.57
1422903_at	NM_010745	Ly86	lymphocyte antigen 86	-1.57
1434735_at	NM_172563	Hlf	hepatic leukemia factor	-1.57
1438667_at	NM_001032727	Sybu	syntabulin (syntaxin-interacting)	-1.57
1448890_at	NM_008452	Klf2	Kruppel-like factor 2 (lung)	-1.57
1428490_at	NM_052993	C1galt1	core 1 synthase, glycoprotein-N-acetylgalactosamine 3-beta-galactosyltransferase, 1	-1.57
1418250_at	NM_025404	Arl4d	ADP-ribosylation factor-like 4D	-1.57
1416286_at	NM_009062	Rgs4	regulator of G-protein signaling 4	-1.57
1437392_at	NM_001085521	Tmem90b	transmembrane protein 90B	-1.57
1417464_at	NM_009394	Tnnc2	troponin C2, fast	-1.57
1419874_x_at	NM_001033324	Zbtb16	zinc finger and BTB domain containing 16	-1.57
1435464_at	NM_133697		RIKEN cDNA 1110003E01 gene	-1.57
1434372_at	NM_001177351		expressed sequence AW112010	-1.57
1434470_at	NM_030725	Syt13	synaptotagmin XIII	-1.57
1436294_at	NM_001190371	Ankrd29	ankyrin repeat domain 29	-1.57
1435865_at	NM_031172		histone cluster 3, H2a /// tripartite motif-containing 17	-1.57
1429399_at	NM_026301	Rnf125	ring finger protein 125	-1.57
1425267_a_at	NM_001032413	Pear1	platelet endothelial aggregation receptor 1	-1.57
1435867_at	NM_001033430	Jhdm1d	jumonji C domain-containing histone demethylase 1 homolog D (S. cerevisiae)	-1.57
1435714_x_at	NM_145837	Il17d	interleukin 17D	-1.57
1454731_at	NM_019472	Myo10	myosin X	-1.57

Probe Set ID	Accession_ID	Gene	Description	Fold
1423803_s_at	NM_133831	Gltscr2	glioma tumor suppressor candidate region gene 2	-1.57
1460197_a_at	NM_054098	Steap4	STEAP family member 4	-1.57
1441946_at	NM_172471	Itih5	inter-alpha (globulin) inhibitor H5	-1.57
1439496_at	NM_029858	Ston1	Stonin 1	-1.57
1426539_at	NM_145628	Usp11	ubiquitin specific peptidase 11	-1.57
1423989_at	NM_027410	Tecpr1	tectonin beta-propeller repeat containing 1	-1.57
1435467_at	NM_053072	Fgd6	FYVE, RhoGEF and PH domain containing 6	-1.57
1429400_at	NM_016691	Clcn5	chloride channel 5	-1.57
1436325_at	NM_013646	Rora	RAR-related orphan receptor alpha	-1.57
1416301_a_at	NM_007897	Ebf1	early B-cell factor 1	-1.56
1423414_at	NM_008969	Ptgs1	prostaglandin-endoperoxide synthase 1	-1.56
1455342_at	NM_181348	Prune2	prune homolog 2 (Drosophila)	-1.56
1426731_at	NM_010043	Des	desmin	-1.56
1453238_s_at	NR_002860		RIKEN cDNA 3930401B19 gene	-1.56
1416322_at	NM_054077	Prelp	proline arginine-rich end leucine-rich repeat	-1.56
1423415_at	NM_010287	Gpr83	G protein-coupled receptor 83	-1.56
1424051_at	NM_009932	Col4a2	collagen, type IV, alpha 2	-1.56
1424374_at	NM_174990	Gimap4	GTPase, IMAP family member 4	-1.56
1455840_at	NM_175930	Rapgef5	Rap guanine nucleotide exchange factor (GEF) 5	-1.56
1439500_at	NM_027268	Scrn1	secernin 1	-1.56
1455031_at	NM_001168304	Cdk19	cyclin-dependent kinase 19	-1.56
1434833_at	NM_009006	Map4k2	mitogen-activated protein kinase kinase kinase kinase 2	-1.56
1452667_at	NM_172601	Rab2b	RAB2B, member RAS oncogene family	-1.56
1454656_at	NM_001033272	Spata13	spermatogenesis associated 13	-1.56
1448942_at	NM_025331	Gng11	guanine nucleotide binding protein (G protein), gamma 11	-1.56
1422953_at	NM_008039	Fpr2	formyl peptide receptor 2	-1.56
1435363_at	NM_001033253	Plekhg1	pleckstrin homology domain containing, family G (with RhoGef domain) member 1	-1.56
1419300_at	NM_010228	Flt1	FMS-like tyrosine kinase 1	-1.56
1419093_at	NM_019911	Tdo2	tryptophan 2,3-dioxygenase	-1.56
1420913_at	NM_033314	Slco2a1	solute carrier organic anion transporter family, member 2a1	-1.56
1447800_x_at	NM_001130458	Tcn2	transcobalamin 2	-1.56
1434428_at	NM_001172216		RIKEN cDNA D330028D13 gene	-1.56
1455160_at	NR_015483		RIKEN cDNA 2610203C20 gene	-1.56
1449630_s_at	NM_145515	Mark1	MAP/microtubule affinity-regulating kinase 1	-1.56
1434329_s_at	NM_197985	Adipor2	adiponectin receptor 2	-1.56
1453141_at	XR_106377		RIKEN cDNA 0610009L18 gene	-1.56
1427923_at	NM_172700	Zmpste24	zinc metallopeptidase, STE24 homolog (S. cerevisiae)	-1.56
1441687_at	NM_009523		wingless-related MMTV integration site 4	-1.56
1428955_x_at	NM_023055	Slc9a3r2	solute carrier family 9 (sodium/hydrogen exchanger), member 3 regulator 2	-1.55
1415850_at	NM_009025	Rasa3	RAS p21 protein activator 3	-1.55
1442063_at	NM_029967	Adamtsl1	ADAMTS-like 1	-1.55
1420630_at	NM_028982		RIKEN cDNA 8430419L09 gene	-1.55
1439854_at	NM_007545	Hrk	harakiri, BCL2 interacting protein (contains only BH3 domain)	-1.55
1422437_at	NM_007737	Col5a2	collagen, type V, alpha 2	-1.55
1432198_at	AK018172			-1.55
1426245_s_at	NM_001162941	Mapre2	microtubule-associated protein, RP/EB family, member 2	-1.55
1426083_a_at	NM_007569	Btg1	B-cell translocation gene 1, anti-proliferative	-1.55
1428861_at	NM_001040397	Filip1l	filamin A interacting protein 1-like	-1.55
1425407_s_at	NM_001170332		C-type lectin domain family 4, member a2	-1.55
1450380_at	NM_134065	Epdr1	ependymin related protein 1 (zebrafish)	-1.55
1452398_at	NM_019588	Plce1	phospholipase C, epsilon 1	-1.55
1426313_at	NM_144541	Bre	brain and reproductive organ-expressed protein	-1.55

Probe Set ID	Accession_ID	Gene	Description	Fold
1419488_at	NM_139064	Tnip2	TNFAIP3 interacting protein 2	-1.55
1434571_at	NM_177151	Vps13b	vacuolar protein sorting 13B (yeast)	-1.55
1416410_at	NM_008776	Pafah1b3	platelet-activating factor acetylhydrolase, isoform 1b, subunit 3	-1.55
1435275_at	NM_183405	Cox6b2	cytochrome c oxidase subunit VIb polypeptide 2	-1.55
1418547_at	NM_009364	Tfpi2	tissue factor pathway inhibitor 2	-1.55
1416658_at	NM_011356	Frzb	frizzled-related protein	-1.55
1427313_at	NM_008967	Ptgir	prostaglandin I receptor (IP)	-1.55
1448613_at	NM_007899	Ecm1	extracellular matrix protein 1	-1.55
1455080_at	NM_001159662	Ppp1r16b	protein phosphatase 1, regulatory (inhibitor) subunit 16B	-1.55
1415929_at	NM_026160	Map1lc3b	microtubule-associated protein 1 light chain 3 beta	-1.55
1454803_a_at	NM_144919	Hdac11	histone deacetylase 11	-1.55
1448902_at	NM_001168475	Ttc23	tetratricopeptide repeat domain 23	-1.55
1456835_at	BB437157			-1.55
1418453_a_at	NM_009721	Atp1b1	ATPase, Na+/K+ transporting, beta 1 polypeptide	-1.55
1437886_at	NM_183390	Klhl6	kelch-like 6 (Drosophila)	-1.55
1425746_at	NM_030021		RIKEN cDNA D730039F16 gene	-1.55
1426454_at	NM_007486	Arhgdib	Rho, GDP dissociation inhibitor (GDI) beta	-1.55
1417649_at	NM_001161624	Cdkn1c	cyclin-dependent kinase inhibitor 1C (P57)	-1.55
1439087_a_at	NM_178149	Pik3ip1	phosphoinositide-3-kinase interacting protein 1	-1.55
1428288_at	NM_010638	Klf9	Kruppel-like factor 9	-1.55
1439036_a_at	NM_009721	Atp1b1	ATPase, Na+/K+ transporting, beta 1 polypeptide	-1.54
1418050_at	NM_008156	Gpld1	glycosylphosphatidylinositol specific phospholipase D1	-1.54
1455044_at	NM_172614	Tmem44	transmembrane protein 44	-1.54
1427885_at	NM_027196	Pold4	polymerase (DNA-directed), delta 4	-1.54
1460235_at	NM_007644	Scarb2	scavenger receptor class B, member 2	-1.54
1434314_s_at	NM_001003955	Rab11fip5	RAB11 family interacting protein 5 (class I)	-1.54
1436033_at	NM_001113283		cDNA sequence BC031353	-1.54
1417963_at	NM_011125	Pltp	phospholipid transfer protein	-1.54
1451738_at	NM_139144	Ogt	O-linked N-acetylglucosamine (GlcNAc) transferase (UDP-N-acetylglucosamine:polypeptide-N-acetylglucosaminyl transferase)	-1.54
1426246_at	NM_011173	Pros1	protein S (alpha)	-1.54
1438685_at	NM_001099319		predicted gene 12942 /// zinc finger, MYM-type 6	-1.54
1418366_at	NM_013549		histone cluster 2, H2aa1	-1.54
1416592_at	NM_053108	Glrx	glutaredoxin	-1.54
1437111_at	NM_001162921	Zc3h12c	zinc finger CCCH type containing 12C	-1.54
1428259_at	NM_181395	Pxdn	peroxidasin homolog (Drosophila)	-1.54
1428026_at	NM_080455	Tshz2	teashirt zinc finger family member 2	-1.54
1454704_at	NM_007644	Scarb2	scavenger receptor class B, member 2	-1.54
1448649_at	NM_007934	Enpep	glutamyl aminopeptidase	-1.54
1452072_at	NM_026793	Myct1	myc target 1	-1.54
1448146_at	NM_025830	Wwp2	WW domain containing E3 ubiquitin protein ligase 2	-1.54
1425826_a_at	NM_001034962	Sorbs1	sorbin and SH3 domain containing 1	-1.54
1449991_at	NM_018729	Cd244	CD244 natural killer cell receptor 2B4	-1.54
1450495_a_at	NM_001083322	Klrk1	killer cell lectin-like receptor subfamily K, member 1	-1.54
1416474_at	NM_020043	Igdcc4	immunoglobulin superfamily, DCC subclass, member 4	-1.54
1454757_s_at	NM_026790	Ifi27l1	interferon, alpha-inducible protein 27 like 1	-1.54
1418367_x_at	NM_013549		histone cluster 2, H2aa1 /// histone cluster 2, H2aa2 /// histone cluster 2, H2ac /// histone cluster 2, H3c1	-1.54
1418862_at	NM_024208	Echdc3	enoyl Coenzyme A hydratase domain containing 3	-1.54
1431645_a_at	NM_008112	Gdi2	guanosine diphosphate (GDP) dissociation inhibitor 2	-1.54
1435933_at	NM_001099298	Scn2a1	sodium channel, voltage-gated, type II, alpha 1	-1.54
1421207_at	NM_001039537	Lif	leukemia inhibitory factor	-1.54
1423652_at	NM_026921	Isca1	iron-sulfur cluster assembly 1 homolog (S. cerevisiae)	-1.54

Probe Set ID	Accession_ID	Gene	Description	Fold
1429726_at	NM_025807	Slc16a9	solute carrier family 16 (monocarboxylic acid transporters), member 9	-1.54
1448655_at	NM_008512	Lrp1	low density lipoprotein receptor-related protein 1	-1.54
1448775_at	NM_001045481	Ifi203	interferon activated gene 203	-1.54
1419186_a_at	NM_001159745	St8sia4	ST8 alpha-N-acetyl-neuraminide alpha-2,8-sialyltransferase 4	-1.54
1424354_at	NM_197986	Tmem140	transmembrane protein 140	-1.54
1419469_at	NM_013531	Gnb4	guanine nucleotide binding protein (G protein), beta 4	-1.54
1448276_at	NM_053082	Tspan4	tetraspanin 4	-1.54
1429310_at	NM_001172160	Flrt3	fibronectin leucine rich transmembrane protein 3	-1.53
1429909_at	NM_001083810	Prr5l	proline rich 5 like	-1.53
1448272_at	NM_007570	Btg2	B-cell translocation gene 2, anti-proliferative	-1.53
1428115_a_at	NM_172601	Rab2b	RAB2B, member RAS oncogene family	-1.53
1421142_s_at	NM_001197321	Foxp1	forkhead box P1	-1.53
1456609_at	NM_025451	Camk2n1	calcium/calmodulin-dependent protein kinase II inhibitor 1	-1.53
1417304_at	NM_009893	Chrd	chordin	-1.53
1449147_at	NM_023850	Chst1	carbohydrate (keratan sulfate Gal-6) sulfotransferase 1	-1.53
1416418_at	NM_020590	Gabarapl1	gamma-aminobutyric acid (GABA) A receptor-associated protein-like 1	-1.53
1415877_at	NM_001136086	Dpysl3	dihydropyrimidinase-like 3	-1.53
1415958_at	NM_009204	Slc2a4	solute carrier family 2 (facilitated glucose transporter), member 4	-1.53
1449082_at	NM_015776	Mfap5	microfibrillar associated protein 5	-1.53
1423141_at	NM_001111100	Lipa	lysosomal acid lipase A	-1.53
1456150_at	NM_001033430	Jhdm1d	jumonji C domain-containing histone demethylase 1 homolog D (S. cerevisiae)	-1.53
1423986_a_at	NM_025858	Shisa5	shisa homolog 5 (Xenopus laevis)	-1.53
1418746_at	NM_001039509	Pnkd	paroxysmal nonkinesiogenic dyskinesia	-1.53
1423690_s_at	NM_153410	Gpsm1	G-protein signalling modulator 1 (AGS3-like, C. elegans)	-1.53
1435740_at	NM_183208		predicted gene 10397 /// zinc finger, MIZ-type containing 1	-1.53
1460178_at	NM_001168591	Lonp2	lon peptidase 2, peroxisomal	-1.53
1418090_at	NM_032398	Plvap	plasmalemma vesicle associated protein	-1.53
1417283_at	NM_011838	Lynx1	Ly6/neurotoxin 1	-1.53
1416695_at	NM_009775	Tspo	translocator protein	-1.53
1427894_at	NM_139307	Vasn	vasorin	-1.53
1451099_at	NM_011843	Esyt1	extended synaptotagmin-like protein 1	-1.53
1460003_at	AV234963		expressed sequence AI956758	-1.53
1418484_at	NM_011902	Tekt2	tektin 2	-1.53
1455558_at	NM_001033298	Plk1s1	polo-like kinase 1 substrate 1	-1.53
1422788_at	NM_021398	Slc43a3	solute carrier family 43, member 3	-1.53
1430012_at	AK004221		RIKEN cDNA 1110050K14 gene	-1.53
1433795_at	NM_011578	Tgfbr3	transforming growth factor, beta receptor III	-1.53
1448228_at	NM_010728	Lox	lysyl oxidase	-1.53
1417440_at	NM_001080819	Arid1a	AT rich interactive domain 1A (SWI-like)	-1.53
1446075_at	AV376107			-1.53
1434930_at	NM_145853	Tpcn1	two pore channel 1	-1.52
1417522_at	NM_026346	Fbxo32	F-box protein 32	-1.52
1449401_at	NM_007574	C1qc	complement component 1, q subcomponent, C chain	-1.52
1439994_at	NM_001145433		RIKEN cDNA 1810013D10 gene	-1.52
1448499_a_at	NM_007940	Ephx2	epoxide hydrolase 2, cytoplasmic	-1.52
1450080_at	NM_028375	Cxx1c	CAAX box 1 homolog C (human)	-1.52
1419994_s_at	NM_025514	Anapc16	anaphase promoting complex subunit 16	-1.52
1422514_at	NM_009636	Aebp1	AE binding protein 1	-1.52
1424268_at	NM_001177833	Smox	spermine oxidase	-1.52
1435222_at	NM_001197321	Foxp1	forkhead box P1	-1.52

Probe Set ID	Accession_ID	Gene	Description	Fold
1446625_at	NM_026433	Tmem100	transmembrane protein 100	-1.52
1422155_at	NM_054045		histone cluster 2, H3c2, pseudogene	-1.52
1443458_at	XM_001001707		RIKEN cDNA D630033O11 gene	-1.52
1453011_at	NM_001172055	Bdh2	3-hydroxybutyrate dehydrogenase, type 2	-1.52
1434068_s_at	NR_015519	AI662270	expressed sequence AI662270	-1.52
1421073_a_at	NM_001136079	Ptger4	prostaglandin E receptor 4 (subtype EP4)	-1.52
1417179_at	NM_019571	Tspan5	tetraspanin 5	-1.52
1418641_at	NM_010696	Lcp2	lymphocyte cytosolic protein 2	-1.52
1424653_at	NM_197996	Tspan15	tetraspanin 15	-1.52
1424421_at	NM_177041	Flad1	RFad1, flavin adenine dinucleotide synthetase, homolog (yeast)	-1.52
1449319_at	NM_138683	Rspo1	R-spondin homolog (Xenopus laevis)	-1.52
1445546_at	AI844685	AI844685	expressed sequence AI844685	-1.52
1417089_a_at	NM_009897	Ckmt1	creatine kinase, mitochondrial 1, ubiquitous	-1.52
1416419_s_at	NM_020590	Gabarapl1	gamma-aminobutyric acid (GABA) A receptor-associated protein-like 1	-1.52
1425779_a_at	NM_011532	Tbx1	T-box 1	-1.52
1423439_at	NM_011044	Pck1	phosphoenolpyruvate carboxykinase 1, cytosolic	-1.52
1448254_at	NM_008973	Ptn	pleiotrophin	-1.52
1419062_at	NM_013813	Epb4.1l3	erythrocyte protein band 4.1-like 3	-1.52
1434315_at	NM_028995	Nipal3	NIPA-like domain containing 3	-1.51
1420277_at	AV245881			-1.51
1452714_at	NM_198294	Tanc1	tetratricopeptide repeat, ankyrin repeat and coiled-coil containing 1	-1.51
1435092_at	NM_001039515	Arl4a	ADP-ribosylation factor-like 4A	-1.51
1458299_s_at	NM_008690	Nfkbie	nuclear factor of kappa light polypeptide gene enhancer in B-cells inhibitor, epsilon	-1.51
1435763_at	NM_172443	Tbc1d16	TBC1 domain family, member 16	-1.51
1444384_at	BB364291	---		-1.51
1428267_at	NM_026191	Dhx40	DEAH (Asp-Glu-Ala-His) box polypeptide 40	-1.51
1416968_a_at	NM_001040684	Hsd3b7	hydroxy-delta-5-steroid dehydrogenase, 3 beta- and steroid delta-isomerase 7	-1.51
1421140_a_at	NM_001197321	Foxp1	forkhead box P1	-1.51
1455515_at	NM_001163145		RIKEN cDNA 1810041L15 gene	-1.51
1426752_at	NM_001130184	Phf17	PHD finger protein 17	-1.51
1429001_at	NM_027153	Pir	pirin	-1.51
1449127_at	NM_009151	Selplg	selectin, platelet (p-selectin) ligand	-1.51
1418939_at	NM_008250	Hlx	H2.0-like homeobox	-1.51
1423413_at	NM_008681	Ndrg1	N-myc downstream regulated gene 1	-1.51
1457701_at	NM_001034863	Tmem136	transmembrane protein 136	-1.51
1417271_a_at	NM_001146348	Eng	endoglin	-1.51
1456480_at	NM_172887	Fry	furry homolog (Drosophila)	-1.51
1421594_a_at	NM_001040085	Sytl2	synaptotagmin-like 2	-1.51
1460295_s_at	NM_010560	Il6st	interleukin 6 signal transducer	-1.51
1421313_s_at	NM_007803	Cttn	cortactin	-1.51
1458426_at	BM941075			-1.51
1438577_at	BB376947			-1.51
1435141_at	NM_145512	Sft2d2	SFT2 domain containing 2	-1.51
1451318_a_at	NM_001111096	Lyn	Yamaguchi sarcoma viral (v-yes-1) oncogene homolog	-1.51
1429273_at	NM_028472	Bmper	BMP-binding endothelial regulator	-1.51
1442640_at	BB530448			-1.51
1417251_at	NM_023245	Palmd	palmdelphin	-1.51
1447929_at	NM_198113	Ssh3	slingshot homolog 3 (Drosophila)	-1.51
1458308_at	NM_183426	Sbno2	strawberry notch homolog 2 (Drosophila)	-1.51
1424762_at	NM_001040631		C1q and tumor necrosis factor related protein 5 /// membrane-type frizzled-related protein	-1.50

Probe Set ID	Accession_ID	Gene	Description	Fold
1419247_at	NM_009061	Rgs2	regulator of G-protein signaling 2	-1.50
1449851_at	NM_001159367	Per1	period homolog 1 (Drosophila)	-1.50
1427200_at	NM_207302	Zranb1	zinc finger, RAN-binding domain containing 1	-1.50
1460553_at	NM_183254		RIKEN cDNA 1700025K23 gene	-1.50
1449049_at	NM_030682	Tlr1	toll-like receptor 1	-1.50
1460437_at	NM_028195	Cyth4	cytohesin 4	-1.50
1443579_s_at	NM_001037937		DEP domain containing 6 /// hypothetical LOC100505173	-1.50
1427232_at	NM_001081300	Tshz1	teashirt zinc finger family member 1	-1.50
1440825_s_at	NM_144820	Ccdc28a	coiled-coil domain containing 28A	-1.50
1438402_at	NM_001081161	Fam171a1	family with sequence similarity 171, member A1	-1.50
1418704_at	NM_009113	S100a13	S100 calcium binding protein A13	-1.50
1419467_at	NM_025809	Clec14a	C-type lectin domain family 14, member a	-1.50
1419075_s_at	NM_009117	Saa1	serum amyloid A 1	-1.50
1417391_a_at	NM_010551	Il16	interleukin 16	-1.50
1416613_at	NM_009994	Cyp1b1	cytochrome P450, family 1, subfamily b, polypeptide 1	-1.50
1427167_at	XM_003086849		armadillo repeat containing, X-linked 4	-1.50
1457266_at	NM_001037717	Slc38a6	solute carrier family 38, member 6	-1.50
1419202_at	NM_009977	Cst7	cystatin F (leukocystatin)	-1.50
1416998_at	NM_021511	Rrs1	RRS1 ribosome biogenesis regulator homolog (S. cerevisiae)	1.50
1456516_x_at	NM_133806		UDP-N-acetylhexosamine pyrophosphorylase-like /// UDP-N-acetylglucosamine pyrophosphorylase 1	1.50
1427408_a_at	NM_146153	Thrap3	thyroid hormone receptor associated protein 3	1.50
1424921_at	NM_198095	Bst2	bone marrow stromal cell antigen 2	1.50
1450914_at	NM_008889	Ppp1r14b	protein phosphatase 1, regulatory (inhibitor) subunit 14B	1.50
1416030_a_at	NM_008568	Mcm7	minichromosome maintenance deficient 7 (S. cerevisiae)	1.50
1449265_at	NM_009807	Casp1	caspase 1	1.50
1433713_at	NM_172719	Gcn1l1	GCN1 general control of amino-acid synthesis 1-like 1 (yeast)	1.50
1434424_at	NM_001081259	Mfsd7b	major facilitator superfamily domain containing 7B	1.50
1428100_at	NM_001078167	Srsf1	serine/arginine-rich splicing factor 1	1.50
1452788_at	NM_012024	Ppp2r5e	protein phosphatase 2, regulatory subunit B (B56), epsilon isoform	1.50
1438305_at	NM_001012623	Rims1	regulating synaptic membrane exocytosis 1	1.50
1437457_a_at	NM_008098	Mtpn	myotrophin	1.50
1418727_at	NM_133227	Nup155	nucleoporin 155	1.50
1429029_at	NM_028943	Sgms2	sphingomyelin synthase 2	1.50
1449186_at	NM_026121	Bag4	BCL2-associated athanogene 4	1.50
1426609_at	NM_028315	Dis3	DIS3 mitotic control homolog (S. cerevisiae)	1.50
1422677_at	NM_026384	Dgat2	diacylglycerol O-acyltransferase 2	1.50
1426621_a_at	NM_027531	Ppp2r2b	protein phosphatase 2 (formerly 2A), regulatory subunit B (PR 52), beta isoform	1.50
1448373_at	NM_026310	Mrpl18	mitochondrial ribosomal protein L18	1.50
1459756_at	NM_153585	Cnot10	CCR4-NOT transcription complex, subunit 10	1.50
1451166_a_at	NM_029339	Ccdc101	coiled-coil domain containing 101	1.50
1439464_s_at	NM_172304	Tex10	testis expressed gene 10	1.50
1452830_s_at	NM_023525	Cad	carbamoyl-phosphate synthetase 2, aspartate transcarbamylase, and dihydroorotase	1.51
1457915_at	NM_177101		RIKEN cDNA 4833442J19 gene	1.51
1418791_at	NM_019535	Sh3gl2	SH3-domain GRB2-like 2	1.51
1439553_s_at	NM_026532		nuclear transport factor 2	1.51
1440870_at	NM_001177995	Prdm16	PR domain containing 16	1.51
1452787_a_at	NM_019830	Prmt1	protein arginine N-methyltransferase 1	1.51
1451363_a_at	NM_001093754	Dennd2d	DENN/MADD domain containing 2D	1.51
1430536_a_at	NM_007951	Erh	enhancer of rudimentary homolog (Drosophila)	1.51
1435054_at	NM_177752	Eme1	essential meiotic endonuclease 1 homolog 1 (S. pombe)	1.51

Probe Set ID	Accession_ID	Gene	Description	Fold
1423696_a_at	NM_025550	Psmd6	proteasome (prosome, macropain) 26S subunit, non-ATPase, 6	1.51
1448113_at	NM_019641	Stmn1	stathmin 1	1.51
1426853_at	NM_023871	Set	SET nuclear oncogene	1.51
1438736_at	NM_001033422	Thoc2	THO complex 2	1.51
1420892_at	NM_001163633	Wnt7b	wingless-related MMTV integration site 7B	1.51
1454831_at	NM_180974	Foxn2	forkhead box N2	1.51
1429537_at	NM_025669	Sfrs18	serine/arginine-rich splicing factor 18	1.51
1450012_x_at	NM_018871	Ywhag	tyrosine 3-monooxygenase/tryptophan 5-monooxygenase activation protein, gamma polypeptide	1.51
1425521_at	NM_001079849	Paip1	polyadenylate binding protein-interacting protein 1	1.51
1450993_at	NM_080850	Pask	PAS domain containing serine/threonine kinase	1.51
1424083_at	NM_144904	Rod1	ROD1 regulator of differentiation 1 (S. pombe)	1.51
1451092_a_at	NM_001146174	Rangap1	RAN GTPase activating protein 1	1.51
1452168_x_at	NM_001130008	Gspt1	G1 to S phase transition 1	1.51
1416746_at	NM_010436	H2afx	H2A histone family, member X	1.51
1460713_at	NM_207161	BC048355	cDNA sequence BC048355	1.51
1436775_a_at	NM_030886	Ankrd17	ankyrin repeat domain 17	1.51
1450847_at	NM_026554	Ncbp2	nuclear cap binding protein subunit 2	1.51
1443161_at	BM240648			1.51
1422463_a_at	NM_053159	Mrpl3	mitochondrial ribosomal protein L3	1.51
1424323_at	NM_021303	Noc2l	nucleolar complex associated 2 homolog (S. cerevisiae)	1.51
1419288_at	NM_023844	Jam2	junction adhesion molecule 2	1.51
1417724_at	NM_011568	Thoc4	THO complex 4	1.51
1416120_at	NM_009104	Rrm2	ribonucleotide reductase M2	1.51
1416773_at	NM_009516	Wee1	WEE 1 homolog 1 (S. pombe)	1.51
1417511_at	NM_025281	Lyar	Ly1 antibody reactive clone	1.51
1455674_at	NM_030234	Wdr76	WD repeat domain 76	1.51
1429326_at	NM_001159930	Cenpl	centromere protein L	1.51
1423852_at	NM_145463	Shisa2	shisa homolog 2 (Xenopus laevis)	1.51
1431829_a_at	NM_023622	Rgl3	ral guanine nucleotide dissociation stimulator-like 3	1.51
1433718_a_at	NM_007622	Cbx1	chromobox homolog 1 (Drosophila HP1 beta)	1.52
1460325_at	NM_001159603	Pum1	pumilio 1 (Drosophila)	1.52
1448638_at	NM_001168250	Mtbp	Mdm2, transformed 3T3 cell double minute p53 binding protein	1.52
1453849_s_at	NM_001048061	Hnrnpab	heterogeneous nuclear ribonucleoprotein A/B	1.52
1422834_at	NM_019697	Kcnd2	potassium voltage-gated channel, Shal-related family, member 2	1.52
1450188_s_at	NM_010720	Lipg	lipase, endothelial	1.52
1439535_at	BB204161			1.52
1439394_x_at	NM_023223	Cdc20	cell division cycle 20 homolog (S. cerevisiae)	1.52
1455841_s_at	NM_153419	Grwd1	glutamate-rich WD repeat containing 1	1.52
1425104_at	NM_001142731	Kctd1	potassium channel tetramerisation domain containing 1	1.52
1417213_a_at	NM_011251	Rbm6	RNA binding motif protein 6	1.52
1450569_a_at	NM_019869	Rbm14	RNA binding motif protein 14	1.52
1448536_at	NM_026309	Lsm3	LSM3 homolog, U6 small nuclear RNA associated (S. cerevisiae)	1.52
1435753_a_at	NM_001145804	Nucks1	nuclear casein kinase and cyclin-dependent kinase substrate 1	1.52
1416423_x_at	NM_001110145	Ssb	Sjogren syndrome antigen B	1.52
1420416_at	NM_009152	Sema3a	sema domain, immunoglobulin domain (Ig), short basic domain, secreted, (semaphorin) 3A	1.52
1435693_at	NM_145532	Mall	mal, T-cell differentiation protein-like	1.52
1448604_at	NM_030724	Uck2	uridine-cytidine kinase 2	1.52
1419034_at	NM_007788	Csnk2a1	casein kinase 2, alpha 1 polypeptide	1.52
1434530_at	NM_011858	Odz4	odd Oz/ten-m homolog 4 (Drosophila)	1.52
1439387_x_at	NM_153775	Ctu2	cytosolic thiouridylase subunit 2 homolog (S. pombe)	1.52
1423700_at	NM_027009	Rfc3	replication factor C (activator 1) 3	1.52
1425895_a_at	NM_010495	Id1	inhibitor of DNA binding 1	1.52

Probe Set ID	Accession_ID	Gene	Description	Fold
1437992_x_at	NM_010288	Gja1	gap junction protein, alpha 1	1.52
1437279_x_at	NM_011519	Sdc1	syndecan 1	1.52
1431692_a_at	NM_001161844	Cblc	Casitas B-lineage lymphoma c	1.52
1435184_at	NM_001039181	Npr3	natriuretic peptide receptor 3	1.52
1420720_at	NM_016789	Nptx2	neuronal pentraxin 2	1.52
1437278_a_at	NM_016682	Uba2	ubiquitin-like modifier activating enzyme 2	1.52
1452972_at	NM_029321	Ttc32	tetratricopeptide repeat domain 32	1.52
1433599_at	NM_013815	Baz1a	bromodomain adjacent to zinc finger domain 1A	1.52
1429558_a_at	NM_138593	Larp7	La ribonucleoprotein domain family, member 7	1.52
1415888_at	NM_008231	Hdgf	hepatoma-derived growth factor	1.52
1460687_at	NM_030241	Setd8	SET domain containing (lysine methyltransferase) 8	1.52
1455831_at	NM_139149	Fus	fusion, derived from t(12;16) malignant liposarcoma (human)	1.52
1416569_at	NM_019673	Actl6a	actin-like 6A	1.52
1434367_s_at	NM_026532		nuclear transport factor 2 /// nuclear transport factor 2, pseudogene 1	1.52
1452364_at	NM_001163018	Suz12	suppressor of zeste 12 homolog (Drosophila)	1.52
1437489_x_at	NM_025848	Sdhd	succinate dehydrogenase complex, subunit D, integral membrane protein	1.52
1447363_s_at	NM_009773	Bub1b	budding uninhibited by benzimidazoles 1 homolog, beta (S. cerevisiae)	1.52
1435544_at	NM_028274		alanyl-tRNA synthetase /// exosome component 6	1.52
1429100_at	NM_001162903	2010109K11Rik	RIKEN cDNA 2010109K11 gene	1.52
1454158_at	NM_001081287	Mpp7	membrane protein, palmitoylated 7 (MAGUK p55 subfamily member 7)	1.52
1418573_a_at	NM_001139511	Raly	hnRNP-associated with lethal yellow	1.52
1422941_at	NM_053116	Wnt16	wingless-related MMTV integration site 16	1.52
1455016_at	NM_025845	Prpf38b	PRP38 pre-mRNA processing factor 38 (yeast) domain containing B	1.52
1436887_x_at	NM_153419	Grwd1	glutamate-rich WD repeat containing 1	1.52
1428870_at	NM_001039351	Nolc1	nucleolar and coiled-body phosphoprotein 1	1.53
1418230_a_at	NM_001193303	Lims1	LIM and senescent cell antigen-like domains 1	1.53
1429362_a_at	NM_030109	Sf3b2	splicing factor 3b, subunit 2	1.53
1423521_at	NM_010721	Lmnb1	lamin B1	1.53
1455887_at	NM_199035	Alg8	asparagine-linked glycosylation 8 homolog (yeast, alpha-1,3-glucosyltransferase)	1.53
1428069_at	NM_025866	Cdca7	cell division cycle associated 7	1.53
1419465_at	NM_028186	Nkd2	naked cuticle 2 homolog (Drosophila)	1.53
1424883_s_at	NM_001195485	Srsf7	serine/arginine-rich splicing factor 7	1.53
1435731_x_at	AV139803	---		1.53
1423302_a_at	NM_018878	Paxip1	PAX interacting (with transcription-activation domain) protein 1	1.53
1448354_at	NM_008062	G6pdx	glucose-6-phosphate dehydrogenase X-linked	1.53
1430309_at	NM_027707	Nipbl	Nipped-B homolog (Drosophila)	1.53
1430991_at	NR_015572		RIKEN cDNA 1810014B01 gene	1.53
1428570_at	NM_001122982	Ccnc	cyclin C	1.53
1454681_at	NM_194055	Esrp1	epithelial splicing regulatory protein 1	1.53
1438546_x_at	NM_007451	Slc25a5	solute carrier family 25 (mitochondrial carrier, adenine nucleotide translocator), member 5	1.53
1416365_at	NM_008302	Hsp90ab1	heat shock protein 90 alpha (cytosolic), class B member 1	1.53
1417200_at	NM_001042485	Tmem183a	transmembrane protein 183A	1.53
1419660_at	NM_025904		RIKEN cDNA 1600012F09 gene	1.53
1429183_at	NM_026163	Pkp2	plakophilin 2	1.53
1452681_at	NM_001105667	Dtymk	deoxythymidylate kinase	1.53
1426326_at	NM_053009	Zfp91	zinc finger protein 91	1.53
1433482_a_at	NM_057172	Fubp1	far upstream element (FUSE) binding protein 1	1.53

Probe Set ID	Accession_ID	Gene	Description	Fold
1437673_at	NM_009524	Wnt5a	wingless-related MMTV integration site 5A	1.53
1438172_x_at	NM_027698	Eri2	exoribonuclease 2	1.53
1455173_at	NM_001130008	Gspt1	G1 to S phase transition 1	1.53
1423089_at	NM_016963	Tmod3	tropomodulin 3	1.53
1455855_x_at	NM_001048061	Hnrnpab	heterogeneous nuclear ribonucleoprotein A/B	1.53
1450011_at	NM_019657	Hsd17b12	hydroxysteroid (17-beta) dehydrogenase 12	1.54
1450915_at	NM_009680	Ap3b1	adaptor-related protein complex 3, beta 1 subunit	1.54
1425179_at	NM_009171	Shmt1	serine hydroxymethyltransferase 1 (soluble)	1.54
1455387_at	NM_001024205	Nufip2	nuclear fragile X mental retardation protein interacting protein 2	1.54
1427266_at	NM_001081251	Pbrm1	polybromo 1	1.54
1424244_at	NM_203507	Rwdd4a	RWD domain containing 4A	1.54
1435826_at	NM_001167730	Rad18	RAD18 homolog (S. cerevisiae)	1.54
1427938_at	NM_019660	Mycbp	c-myc binding protein	1.54
1440409_at	NM_028087	Gcnt3	glucosaminyl (N-acetyl) transferase 3, mucin type	1.54
1434240_at	NM_001080995		RIKEN cDNA 4632434I11 gene	1.54
1424349_a_at	NM_001134829	Lpgat1	lysophosphatidylglycerol acyltransferase 1	1.54
1459734_at	BB737833			1.54
1415994_at	NM_021282	Cyp2e1	cytochrome P450, family 2, subfamily e, polypeptide 1	1.54
1431304_a_at	NM_001042485	Tmem183a	transmembrane protein 183A	1.54
1460593_at	NM_144796	Susd4	sushi domain containing 4	1.54
1418371_at	NM_001168471	Dynll2	dynein light chain LC8-type 2	1.54
1448896_at	NM_008838	Pigf	phosphatidylinositol glycan anchor biosynthesis, class F	1.54
1430053_a_at	NM_025942	Ola1	Obg-like ATPase 1	1.54
1419503_at	NM_011491	Stc2	stanniocalcin 2	1.54
1434432_at	NM_001007465	Rffl	ring finger and FYVE like domain containing protein	1.54
1434007_at	NM_001166633	Gyltl1b	glycosyltransferase-like 1B	1.54
1423421_at	NM_019683	Ankrd49	ankyrin repeat domain 49	1.54
1451080_at	NM_146144	Usp1	ubiquitin specific peptidase 1	1.54
1427037_at	NM_001005331	Eif4g1	eukaryotic translation initiation factor 4, gamma 1	1.54
1423137_at	NM_019491	Rala	v-ral simian leukemia viral oncogene homolog A (ras related)	1.54
1417577_at	NM_019510	Trpc3	transient receptor potential cation channel, subfamily C, member 3	1.54
1419046_at	NM_021555	Brp16	brain protein 16	1.54
1434882_at	NM_026002	Mtdh	metadherin	1.54
1437267_x_at	NM_021510	Hnrnph1	Heterogeneous nuclear ribonucleoprotein H1	1.54
1453836_a_at	NM_001166249	Mgll	monoglyceride lipase	1.54
1450416_at	NM_001076789	Cbx5	chromobox homolog 5 (Drosophila HP1a)	1.54
1443978_at	NM_172756	Ankle1	ankyrin repeat and LEM domain containing 1	1.54
1429633_at	NM_172154	Lcor	ligand dependent nuclear receptor corepressor	1.54
1437716_x_at	NM_145588	Kif22	kinesin family member 22	1.54
1438955_x_at	NM_134084	Ppif	peptidylprolyl isomerase F (cyclophilin F)	1.54
1441272_at	NM_010771	Matr3	Matrin 3	1.54
1424140_at	NM_178389	Gale	galactose-4-epimerase, UDP	1.54
1439047_s_at	NM_023042	Recql	RecQ protein-like	1.54
1417181_a_at	NM_010629	Kifap3	kinesin-associated protein 3	1.54
1450854_at	NM_011119	Pa2g4	proliferation-associated 2G4	1.55
1416335_at	NM_010798	Mif	macrophage migration inhibitory factor	1.55
1416184_s_at	NM_001025427		high mobility group AT-hook 1	1.55
1437422_at	NM_009154	Sema5a	sema domain, seven thrombospondin repeats (type 1 and type 1-like), transmembrane domain (TM) and short cytoplasmic domain, (semaphorin) 5A	1.55
1415800_at	NM_010288	Gja1	gap junction protein, alpha 1	1.55
1416485_at	NM_016897	Timm23	translocase of inner mitochondrial membrane 23 homolog (yeast)	1.55

Probe Set ID	Accession_ID	Gene	Description	Fold
1426831_at	NM_145542	Ahcyl1	S-adenosylhomocysteine hydrolase-like 1	1.55
1436723_at	NM_145924	Cenpi	centromere protein I	1.55
1456244_x_at	NM_023140	Glrx3	glutaredoxin 3	1.55
1436505_at	NM_001081086	Ppig	peptidyl-prolyl isomerase G (cyclophilin G)	1.55
1431506_s_at	NM_001110129	Ppih	peptidyl prolyl isomerase H	1.55
1438093_x_at	NM_001037999	Dbi	diazepam binding inhibitor	1.55
1454992_at	NM_007513	Slc7a1	solute carrier family 7 (cationic amino acid transporter, y+ system), member 1	1.55
1453240_a_at	NM_027045	Gcap14	granule cell antiserum positive 14	1.55
1418566_s_at	NM_026023	Nudcd2	NudC domain containing 2	1.55
1432187_at	NM_145706	Nup43	nucleoporin 43	1.55
1423461_a_at	NM_011908	Ubl3	ubiquitin-like 3	1.55
1456730_x_at	NM_019673	Actl6a	actin-like 6A	1.55
1420935_a_at	NM_001130477	Srrm1	serine/arginine repetitive matrix 1	1.55
1422508_at	NM_007508	Atp6v1a	ATPase, H+ transporting, lysosomal V1 subunit A	1.55
1417587_at	NM_001136082	Timeless	timeless homolog (Drosophila)	1.55
1437494_at	NM_178907	Mapkapk3	mitogen-activated protein kinase-activated protein kinase 3	1.55
1439740_s_at	NM_030724	Uck2	uridine-cytidine kinase 2	1.55
1441911_x_at	NM_010256	Gart	phosphoribosylglycinamide formyltransferase	1.55
1428970_at	NM_028108	Naa50	N(alpha)-acetyltransferase 50, NatE catalytic subunit	1.55
1419755_at	NM_013900	Mfi2	antigen p97 (melanoma associated) identified by monoclonal antibodies 133.2 and 96.5	1.55
1458439_a_at	NM_001110017	Dzip3	DAZ interacting protein 3, zinc finger	1.55
1426323_x_at	NM_001161737	Siva1	SIVA1, apoptosis-inducing factor	1.55
1436509_at	NM_175403	Mlec	malectin	1.55
1417878_at	NM_007891	E2f1	E2F transcription factor 1	1.55
1426958_at	NM_029767	Rps9	ribosomal protein S9	1.55
1429095_at	NM_025495	Cenpp	centromere protein P	1.55
1449194_at	NM_025578	Mrps25	mitochondrial ribosomal protein S25	1.55
1443867_at	NM_001025572	Ankrd12	ankyrin repeat domain 12	1.55
1438250_s_at	NM_001015889		TAF9 RNA polymerase II, TATA box binding protein (TBP)-associated factor	1.56
1424778_at	NM_178606	Reep3	receptor accessory protein 3	1.56
1434583_at	NM_177794	Tmem26	transmembrane protein 26	1.56
1456240_x_at	NM_028023	Cdca4	cell division cycle associated 4	1.56
1440383_at	NM_001025312	Dclre1b	DNA cross-link repair 1B, PSO2 homolog (S. cerevisiae)	1.56
1428527_at	NM_001190156	Snx7	sorting nexin 7	1.56
1438096_a_at	NM_001105667	Dtymk	deoxythymidylate kinase	1.56
1440199_at	NM_177809	Slc25a48	solute carrier family 25, member 48	1.56
1455162_at	NM_001145948	Ttc39a	tetratricopeptide repeat domain 39A	1.56
1426741_a_at	NM_172422	Fastkd2	FAST kinase domains 2	1.56
1416873_a_at	NM_016756	Cdk2	cyclin-dependent kinase 2	1.56
1422946_a_at	NM_010066	Dnmt1	DNA methyltransferase (cytosine-5) 1	1.56
1417911_at	NM_009828	Ccna2	cyclin A2	1.56
1420946_at	NM_009530	Atrx	alpha thalassemia/mental retardation syndrome X-linked homolog (human)	1.56
1453067_at	NM_027263	Apitd1	apoptosis-inducing, TAF9-like domain 1	1.56
1423149_at	NM_011543	Skp1a	S-phase kinase-associated protein 1A	1.56
1418820_s_at	NM_026479	Zcchc10	zinc finger, CCHC domain containing 10	1.56
1438631_x_at	NM_145607	Ttc13	tetratricopeptide repeat domain 13	1.56
1456871_a_at	NM_001081409	Phf20l1	PHD finger protein 20-like 1	1.56
1424600_at	NM_001161621	Abp1	amiloride binding protein 1 (amine oxidase, copper-containing)	1.56
1416627_at	NM_016907	Spint1	serine protease inhibitor, Kunitz type 1	1.56
1460036_at	NM_026887	Ap1s2	adaptor-related protein complex 1, sigma 2 subunit	1.56

Probe Set ID	Accession_ID	Gene	Description	Fold
1439510_at	NM_028232	Sgol1	shugoshin-like 1 (S. pombe)	1.56
1424573_at	NM_028876	Tmed5	transmembrane emp24 protein transport domain containing 5	1.56
1444004_at	NM_001033422	Thoc2	THO complex 2	1.56
1416354_at	NM_001166623	Rbmx	RNA binding motif protein, X chromosome	1.56
1439887_at	NM_001160368	Rnf152	ring finger protein 152	1.56
1438992_x_at	NM_009716	Atf4	activating transcription factor 4	1.56
1460448_s_at	NM_025978	Ttc14	tetratricopeptide repeat domain 14	1.56
1435534_a_at	NM_024214	Tomm20	translocase of outer mitochondrial membrane 20 homolog (yeast)	1.56
1420021_s_at	NM_001163018	Suz12	suppressor of zeste 12 homolog (Drosophila)	1.56
1449182_at	NM_022984	Retn	resistin	1.56
1419294_at	NM_025956		RIKEN cDNA 1700011H14 gene	1.56
1430875_a_at	NM_026550	Pak1ip1	PAK1 interacting protein 1	1.56
1447640_s_at	NM_016768	Pbx3	pre B-cell leukemia transcription factor 3	1.56
1450093_s_at	NM_010731	Zbtb7a	zinc finger and BTB domain containing 7a	1.56
1418831_at	NM_001162924	Pkp3	plakophilin 3	1.56
1418713_at	NM_025273	Pcbd1	pterin 4 alpha carbinolamine dehydratase/dimerization cofactor of hepatocyte nuclear factor 1 alpha (TCF1) 1	1.56
1417222_a_at	NM_133739	Tmem123	transmembrane protein 123	1.56
1434512_x_at	NM_013663	Srsf3	serine/arginine-rich splicing factor 3	1.56
1450692_at	NM_008446	Kif4	kinesin family member 4	1.57
1448651_at	NM_016918	Nudt5	nudix (nucleoside diphosphate linked moiety X)-type motif 5	1.57
1420249_s_at	NM_009139	Ccl6	chemokine (C-C motif) ligand 6	1.57
1417026_at	NM_026027	Pfdn1	prefoldin 1	1.57
1436413_at	NM_001159544	Frk	fyn-related kinase	1.57
1455834_x_at	NM_001040435	Tacc3	transforming, acidic coiled-coil containing protein 3	1.57
1460698_a_at	NM_025468	Sec11c	SEC11 homolog C (S. cerevisiae)	1.57
1460354_a_at	NM_026759	Mrpl13	mitochondrial ribosomal protein L13	1.57
1455011_at	NM_133774	Stard4	StAR-related lipid transfer (START) domain containing 4	1.57
1417319_at	NM_021495	Pvrl3	poliovirus receptor-related 3	1.57
1418565_at	NM_001113564	Serbp1	serpine1 mRNA binding protein 1	1.57
1431893_a_at	NM_019501	Pdss1	prenyl (solanesyl) diphosphate synthase, subunit 1	1.57
1434942_at	NM_001081090	Esf1	ESF1, nucleolar pre-rRNA processing protein, homolog (S. cerevisiae)	1.57
1439444_x_at	NM_026775	Tmed10	transmembrane emp24-like trafficking protein 10 (yeast)	1.57
1456566_x_at	NM_019869	Rbm14	RNA binding motif protein 14	1.57
1422993_s_at	NM_011568		RNA and export factor binding protein 2 /// THO complex 4	1.57
1454842_a_at	NM_178640	B3galnt2	UDP-GalNAc:betaGlcNAc beta 1,3-galactosaminyltransferase, polypeptide 2	1.57
1429146_at	NM_001160345	Svip	small VCP/p97-interacting protein	1.57
1431054_at	NM_001191004	Lsm6	LSM6 homolog, U6 small nuclear RNA associated (S. cerevisiae)	1.57
1448586_at	NM_015765	Hspa14	heat shock protein 14	1.57
1433991_x_at	NM_001037999	Dbi	diazepam binding inhibitor	1.57
1416150_a_at	NM_013663	Srsf3	serine/arginine-rich splicing factor 3	1.57
1419256_at	NM_009260	Spnb2	spectrin beta 2	1.57
1437480_at	NM_025377	Fam33a	family with sequence similarity 33, member A	1.57
1422910_s_at	NM_025695	Smc6	structural maintenance of chromosomes 6	1.57
1418456_a_at	NM_019568	Cxcl14	chemokine (C-X-C motif) ligand 14	1.57
1434850_at	NM_001033484	Iqgap3	IQ motif containing GTPase activating protein 3	1.57
1422979_at	NM_022724	Suv39h2	suppressor of variegation 3-9 homolog 2 (Drosophila)	1.57
1433962_at	NM_001099792	Trmt61a	tRNA methyltransferase 61 homolog A (S. cerevisiae)	1.57
1422982_at	NM_013476	Ar	androgen receptor	1.57
1452983_at	NM_026665	Cep57	centrosomal protein 57	1.58
1454161_s_at	NM_021446		RIKEN cDNA 0610007P14 gene	1.58

Probe Set ID	Accession_ID	Gene	Description	Fold
1434554_at	NM_197987	Trim37	tripartite motif-containing 37	1.58
1438976_x_at	NM_145569	Mat2a	methionine adenosyltransferase II, alpha	1.58
1435333_at	NM_026742	Ndufaf4	NADH dehydrogenase (ubiquinone) 1 alpha subcomplex, assembly factor 4	1.58
1432188_s_at	NM_145706	Nup43	nucleoporin 43	1.58
1421945_a_at	NM_001042556	Rpf2	ribosome production factor 2 homolog (S. cerevisiae)	1.58
1428902_at	NM_021439	Chst11	carbohydrate sulfotransferase 11	1.58
1449044_at	NM_025380	Eef1e1	eukaryotic translation elongation factor 1 epsilon 1	1.58
1456541_x_at	NM_179203	Atad3a	ATPase family, AAA domain containing 3A	1.58
1447899_x_at	NM_008532	Epcam	epithelial cell adhesion molecule	1.58
1426808_at	NM_001145953	Lgals3	lectin, galactose binding, soluble 3	1.58
1423117_at	NM_001159603	Pum1	pumilio 1 (Drosophila)	1.58
1451640_a_at	NM_001005523	Rsrc2	arginine/serine-rich coiled-coil 2	1.58
1439200_x_at	BE686792			1.58
1416152_a_at	NM_013663	Srsf3	serine/arginine-rich splicing factor 3	1.58
1455904_at	NR_002840		growth arrest specific 5 /// small nucleolar RNA, C/D box 47	1.58
1418023_at	NM_053089	Naa15	N(alpha)-acetyltransferase 15, NatA auxiliary subunit	1.58
1429769_at	NM_172627	Pggt1b	protein geranylgeranyltransferase type I, beta subunit	1.58
1456055_x_at	NM_011131	Pold1	polymerase (DNA directed), delta 1, catalytic subunit	1.58
1416439_at	NM_023203	Dctpp1	dCTP pyrophosphatase 1	1.58
1426755_at	NM_175451	Ckap4	cytoskeleton-associated protein 4	1.58
1422005_at	NM_011163	Eif2ak2	eukaryotic translation initiation factor 2-alpha kinase 2	1.58
1452210_at	NM_177372	Dna2	DNA replication helicase 2 homolog (yeast)	1.58
1434300_at	NM_001114977		RIKEN cDNA 2610101N10 gene	1.58
1417575_at	NM_001177841	Otub2	OTU domain, ubiquitin aldehyde binding 2	1.58
1435122_x_at	NM_010066	Dnmt1	DNA methyltransferase (cytosine-5) 1	1.58
1450744_at	NM_138953	Ell2	elongation factor RNA polymerase II 2	1.58
1429415_at	NM_027230	Zmynd8	zinc finger, MYND-type containing 8	1.58
1455726_at	NM_001033236	Gm71	predicted gene 71	1.58
1450850_at	NM_009510	Ezr	ezrin	1.58
1419452_at	NM_001159866	Uchl5	ubiquitin carboxyl-terminal esterase L5	1.58
1456492_at	NM_001167939		RIKEN cDNA 9130404D08 gene	1.59
1448531_at	NM_010722	Lmnb2	lamin B2	1.59
1456617_a_at	NM_026030	Eif2s2	eukaryotic translation initiation factor 2, subunit 2 (beta)	1.59
1440916_at	NM_001101431		RIKEN cDNA 2510049J12 gene	1.59
1427147_at	NM_199467		RIKEN cDNA F730047E07 gene	1.59
1448953_at	NM_001042527	Blm	Bloom syndrome, RecQ helicase-like	1.59
1415988_at	NM_133808	Hdlbp	high density lipoprotein (HDL) binding protein	1.59
1452232_at	NM_001167981	Galnt7	UDP-N-acetyl-alpha-D-galactosamine: polypeptide N-acetylgalactosaminyltransferase 7	1.59
1429882_at	NR_028428		cadherin 11 pseudogene	1.59
1438016_at	NM_001030307	Dkc1	dyskeratosis congenita 1, dyskerin homolog (human)	1.59
1440373_at	BB041057			1.59
1420019_at	NM_001168679	Tspan8	Tetraspanin 8	1.59
1447877_x_at	NM_010066	Dnmt1	DNA methyltransferase (cytosine-5) 1	1.59
1450982_at	NM_012030	Slc9a3r1	solute carrier family 9 (sodium/hydrogen exchanger), member 3 regulator 1	1.59
1441733_s_at	NM_175749	Nup153	nucleoporin 153	1.59
1449040_a_at	NM_009266	Sephs2	selenophosphate synthetase 2	1.59
1457755_at	NM_010320	Gng8	guanine nucleotide binding protein (G protein), gamma 8	1.59
1438115_a_at	NM_012030	Slc9a3r1	solute carrier family 9 (sodium/hydrogen exchanger), member 3 regulator 1	1.59
1415916_a_at	NM_138745	Mthfd1	methylenetetrahydrofolate dehydrogenase (NADP+ dependent), methenyltetrahydrofolate cyclohydrolase, formyltetrahydrofolate synthase	1.59

Probe Set ID	Accession_ID	Gene	Description	Fold
1428797_at	NM_001035123	Setd6	SET domain containing 6	1.59
1434056_a_at	NM_001033305	Ndufb6	NADH dehydrogenase (ubiquinone) 1 beta subcomplex, 6	1.59
1447919_x_at	NM_028177	Ndufab1	NADH dehydrogenase (ubiquinone) 1, alpha/beta subcomplex, 1	1.59
1443827_x_at	NM_030565	Fam20c	family with sequence similarity 20, member C	1.59
1454169_a_at	NM_029495	Epsti1	epithelial stromal interaction 1 (breast)	1.59
1418443_at	NM_001035226	Xpo1	exportin 1, CRM1 homolog (yeast)	1.59
1459838_s_at	NM_001017525	Btbd11	BTB (POZ) domain containing 11	1.59
1420626_at	NM_026113	Gtf3c6	general transcription factor IIIC, polypeptide 6, alpha	1.59
1423201_at	NM_011308	Ncor1	nuclear receptor co-repressor 1	1.59
1422513_at	NM_007634	Ccnf	cyclin F	1.59
1452036_a_at	NM_001080129	Tmpo	thymopoietin	1.60
1451456_at	NM_198652	Hjurp	Holliday junction recognition protein	1.60
1417233_at	NM_133928	Chchd4	coiled-coil-helix-coiled-coil-helix domain containing 4	1.60
1430134_a_at	NM_198246	Yars2	tyrosyl-tRNA synthetase 2 (mitochondrial)	1.60
1424784_at	NM_001083918	Gm13139	predicted gene 13139	1.60
1426607_at	NM_001039244	Gm7120	predicted gene 7120	1.60
1426739_at	NM_021720	Donson	downstream neighbor of SON	1.60
1417037_at	NM_001163791	Orc6	origin recognition complex, subunit 6	1.60
1428389_s_at	NM_175639	Wdr43	WD repeat domain 43	1.60
1437526_x_at	NM_028871	Hnrnpr	heterogeneous nuclear ribonucleoprotein R	1.60
1455983_at	NM_001110162	Cdca2	cell division cycle associated 2	1.60
1451128_s_at	NM_145588	Kif22	kinesin family member 22	1.60
1434275_at	NM_028186	Nkd2	naked cuticle 2 homolog (Drosophila)	1.60
1435005_at	NM_173762	Cenpe	centromere protein E	1.60
1417921_at	NM_026312		RIKEN cDNA 2610029G23 gene	1.60
1434851_s_at	NM_177638	Crb3	crumbs homolog 3 (Drosophila)	1.60
1418369_at	NM_008921	Prim1	DNA primase, p49 subunit	1.60
1457032_at	NM_001081277	Ak5	adenylate kinase 5	1.60
1449116_a_at	NM_001105667	Dtymk	deoxythymidylate kinase	1.60
1438064_at	NM_011732	Ybx1	Y box protein 1	1.60
1440816_x_at	NM_134040	Ddx1	DEAD (Asp-Glu-Ala-Asp) box polypeptide 1	1.60
1425533_a_at	NM_001111272	Stau2	staufen (RNA binding protein) homolog 2 (Drosophila)	1.60
1437633_at	NM_001081379	Ankrd11	ankyrin repeat domain 11	1.60
1424019_at	NM_138747	Nop2	NOP2 nucleolar protein homolog (yeast)	1.60
1420947_at	NM_009530	Atrx	alpha thalassemia/mental retardation syndrome X-linked homolog (human)	1.60
1433540_x_at	NM_172707	Ppp1cb	protein phosphatase 1, catalytic subunit, beta isoform	1.60
1434201_at	NM_001114385	Chrdl1	chordin-like 1	1.60
1434158_at	NM_146041	Gmds	GDP-mannose 4, 6-dehydratase	1.60
1421817_at	NM_010344	Gsr	glutathione reductase	1.60
1436746_at	NM_001185020	Wnk1	WNK lysine deficient protein kinase 1	1.61
1429720_at	NM_030153	Naa35	N(alpha)-acetyltransferase 35, NatC auxiliary subunit	1.61
1435372_a_at	NM_011119	Pa2g4	proliferation-associated 2G4	1.61
1440177_at	NM_001081097	Grik3	glutamate receptor, ionotropic, kainate 3	1.61
1454725_at	NM_198102	Tra2a	transformer 2 alpha homolog (Drosophila)	1.61
1424641_a_at	NM_153552	Thoc1	THO complex 1	1.61
1436946_s_at	NM_010318		guanine nucleotide binding protein (G protein), gamma 5	1.61
1433569_x_at	NM_009391	Ran	RAN, member RAS oncogene family	1.61
1424136_a_at	NM_001110129		peptidyl-prolyl cis-trans isomerase H-like	1.61
1456590_x_at	NM_009658	Akr1b3	aldo-keto reductase family 1, member B3 (aldose reductase)	1.61
1439436_x_at	NM_016692	Incenp	inner centromere protein	1.61
1451649_a_at	NM_028599	Wdr75	WD repeat domain 75	1.61
1448690_at	NM_008430	Kcnk1	potassium channel, subfamily K, member 1	1.61

Probe Set ID	Accession_ID	Gene	Description	Fold
1417373_a_at	NM_009447	Tuba4a	tubulin, alpha 4A	1.61
1422546_at	NM_001042707	Ilf3	interleukin enhancer binding factor 3	1.61
1417767_at	NM_025558	Cyb5b	cytochrome b5 type B	1.61
1420477_at	NM_001146707	Nap1l1	nucleosome assembly protein 1-like 1	1.61
1423982_at	NM_001080387	Srsf10	serine/arginine-rich splicing factor 10	1.61
1452242_at	NM_001164362	Cep55	centrosomal protein 55	1.61
1453794_at	NM_001136556	Fer1l4	fer-1-like 4 (C. elegans)	1.61
1421230_a_at	NM_054043	Msi2	Musashi homolog 2 (Drosophila)	1.61
1438097_at	NM_011227	Rab20	RAB20, member RAS oncogene family	1.61
1449015_at	NM_020509	Retnla	resistin like alpha	1.61
1418428_at	NM_008448	Kif5b	kinesin family member 5B	1.62
1435484_at	NM_017391	Slc5a3	solute carrier family 5 (inositol transporters), member 3	1.62
1451457_at	NM_172769	Sc5d	sterol-C5-desaturase (fungal ERG3, delta-5-desaturase) homolog (S. cerevisae)	1.62
1418206_at	NM_022324	Sdf2l1	stromal cell-derived factor 2-like 1	1.62
1423766_at	NM_026550	Pak1ip1	PAK1 interacting protein 1	1.62
1429261_at	NM_029384		RIKEN cDNA 2210411K11 gene	1.62
1416962_at	NM_001197082	Rcc1	regulator of chromosome condensation 1	1.62
1449846_at	NM_007895	Ear2	eosinophil-associated, ribonuclease A family, member 2	1.62
1456475_s_at	NM_011158	Prkar2b	protein kinase, cAMP dependent regulatory, type II beta	1.62
1433640_at	NM_057172	Fubp1	far upstream element (FUSE) binding protein 1	1.62
1456262_at	NM_148930	Rbm5	RNA binding motif protein 5	1.62
1451345_at	NM_024433	Mtap	methylthioadenosine phosphorylase	1.62
1417445_at	NM_023294	Ndc80	NDC80 homolog, kinetochore complex component (S. cerevisiae)	1.62
1449434_at	NM_007606	Car3	carbonic anhydrase 3	1.62
1442109_at	BE989344			1.62
1417835_at	NM_008645	Mug1	murinoglobulin 1	1.62
1419522_at	NM_026021	Zmynd19	zinc finger, MYND domain containing 19	1.62
1422944_a_at	NM_019670	Diap3	diaphanous homolog 3 (Drosophila)	1.62
1424180_a_at	NM_011869	Med24	mediator complex subunit 24	1.62
1430530_s_at	NM_026393	Nmral1	NmrA-like family domain containing 1	1.62
1428232_at	NM_001013391	Cpsf6	cleavage and polyadenylation specific factor 6	1.62
1460256_at	NM_007606	Car3	carbonic anhydrase 3	1.62
1435659_a_at	NM_009415	Tpi1	triosephosphate isomerase 1	1.62
1438761_a_at	NM_013614	Odc1	ornithine decarboxylase, structural 1	1.62
1438445_at	NM_028175	Lrrc8e	leucine rich repeat containing 8 family, member E	1.62
1436298_x_at	NM_025939	Paics	phosphoribosylaminoimidazole carboxylase, phosphoribosylaminoribosylaminoimidazole, succinocarboxamide synthetase	1.62
1439377_x_at	NM_023223	Cdc20	cell division cycle 20 homolog (S. cerevisiae)	1.62
1423120_at	NM_031156	Ide	insulin degrading enzyme	1.62
1417569_at	NM_001170866	Ncald	neurocalcin delta	1.62
1417214_at	NM_001082553	Rab27b	RAB27b, member RAS oncogene family	1.62
1418475_at	NM_011325	Scnn1b	sodium channel, nonvoltage-gated 1 beta	1.62
1416915_at	NM_010830	Msh6	mutS homolog 6 (E. coli)	1.62
1437872_at	NM_178728	Napepld	N-acyl phosphatidylethanolamine phospholipase D	1.62
1429678_at	NM_027482		RIKEN cDNA 5730508B09 gene	1.62
1438677_at	NM_026361	Pkp4	plakophilin 4	1.63
1451485_at	NM_026313	Luc7l3	LUC7-like 3 (S. cerevisiae)	1.63
1455990_at	NM_024245	Kif23	kinesin family member 23	1.63
1436533_at	NM_013835	Trove2	TROVE domain family, member 2	1.63
1438545_at	NM_007451	Slc25a5	solute carrier family 25 (mitochondrial carrier, adenine nucleotide translocator), member 5	1.63
1455488_at	NM_173400	Haus6	HAUS augmin-like complex, subunit 6	1.63

Probe Set ID	Accession_ID	Gene	Description	Fold
1416422_a_at	NM_001110145	Ssb	Sjogren syndrome antigen B	1.63
1435597_at	NM_001029856	Atad5	ATPase family, AAA domain containing 5	1.63
1429588_at	NM_026054		RIKEN cDNA 2810474O19 gene	1.63
1426777_a_at	NM_001167745	Wasl	Wiskott-Aldrich syndrome-like (human)	1.63
1422734_a_at	NM_010848	Myb	myeloblastosis oncogene	1.63
1427063_at	NM_001033304		RIKEN cDNA 5330417C22 gene	1.63
1424991_s_at	NM_021288		thymidylate synthase /// thymidylate synthase, pseudogene	1.63
1450842_a_at	NM_007681	Cenpa	centromere protein A	1.63
1426946_at	NM_023579	Ipo5	importin 5	1.63
1453596_at	NM_010496	Id2	inhibitor of DNA binding 2	1.63
1434210_s_at	NM_008377	Lrig1	leucine-rich repeats and immunoglobulin-like domains 1	1.63
1426349_s_at	NM_001080129	Tmpo	thymopoietin	1.63
1438091_a_at	NM_016750		H2A histone family, member Z /// hypothetical LOC100504949	1.63
1453146_at	NM_025709	Gapvd1	GTPase activating protein and VPS9 domains 1	1.63
1434678_at	NM_134163	Mbnl3	muscleblind-like 3 (Drosophila)	1.63
1450401_at	NM_054089	Tgs1	trimethylguanosine synthase homolog (S. cerevisiae)	1.63
1444740_at	NM_001164201	Lass3	LAG1 homolog, ceramide synthase 3	1.63
1452457_a_at	NM_031869	Prkab1	protein kinase, AMP-activated, beta 1 non-catalytic subunit	1.63
1416144_a_at	NM_001042620	Dhx15	DEAH (Asp-Glu-Ala-His) box polypeptide 15	1.63
1456874_at	NM_201518	Flrt2	fibronectin leucine rich transmembrane protein 2	1.63
1438527_at	NM_013762		predicted gene 5879 /// ribosomal protein L3	1.63
1452438_s_at	NM_001081092	Taf4a	TAF4A RNA polymerase II, TATA box binding protein (TBP)-associated factor	1.63
1452741_s_at	NM_001145820	Gpd2	glycerol phosphate dehydrogenase 2, mitochondrial	1.63
1439462_x_at	NM_026775	Tmed10	transmembrane emp24-like trafficking protein 10 (yeast)	1.63
1422547_at	NM_011239	Ranbp1	RAN binding protein 1	1.63
1427504_s_at	NM_011358	Srsf2	serine/arginine-rich splicing factor 2	1.63
1454993_a_at	NM_013663	Srsf3	serine/arginine-rich splicing factor 3	1.63
1454943_a_at	NM_018878	Paxip1	PAX interacting (with transcription-activation domain) protein 1	1.63
1435325_at	NM_177561	Usp46	ubiquitin specific peptidase 46	1.64
1451884_a_at	NM_001110101	Lsm2	LSM2 homolog, U6 small nuclear RNA associated (S. cerevisiae)	1.64
1416290_a_at	NM_011874	Psmc4	proteasome (prosome, macropain) 26S subunit, ATPase, 4	1.64
1417923_at	NM_001195046	Pak3	p21 protein (Cdc42/Rac)-activated kinase 3	1.64
1449176_a_at	NM_007832	Dck	deoxycytidine kinase	1.64
1454694_a_at	NM_011623	Top2a	topoisomerase (DNA) II alpha	1.64
1417075_at	NM_025591	Fam136a	family with sequence similarity 136, member A	1.64
1433893_s_at	NM_017407	Spag5	sperm associated antigen 5	1.64
1449446_at	NM_025602	Ccdc59	coiled-coil domain containing 59	1.64
1423682_a_at	NM_028023	Cdca4	cell division cycle associated 4	1.64
1451610_at	NM_153576	Cxcl17	chemokine (C-X-C motif) ligand 17	1.64
1423066_at	NM_007872	Dnmt3a	DNA methyltransferase 3A	1.64
1450677_at	NM_007691	Chek1	checkpoint kinase 1 homolog (S. pombe)	1.64
1422666_at	NM_001161844	Cblc	Casitas B-lineage lymphoma c	1.64
1449321_x_at	NM_009243		serine (or cysteine) peptidase inhibitor, clade A, member 1A /	1.64
1435336_at	NM_001004177	Celsr2	cadherin, EGF LAG seven-pass G-type receptor 2 (flamingo homolog, Drosophila)	1.64
1422556_at	NM_010303	Gna13	guanine nucleotide binding protein, alpha 13	1.64
1416433_at	NM_011284	Rpa2	replication protein A2	1.64
1451208_at	NM_144866	Etf1	eukaryotic translation termination factor 1	1.64
1418035_a_at	NM_008922	Prim2	DNA primase, p58 subunit	1.64
1460041_at	NM_201411	Flrt1	fibronectin leucine rich transmembrane protein 1	1.64
1420023_at	NM_144866	Etf1	eukaryotic translation termination factor 1	1.64
1439269_x_at	NM_008568	Mcm7	minichromosome maintenance deficient 7 (S. cerevisiae)	1.64
1416480_a_at	NM_001112668		predicted gene 9790 /// HIG1 domain family, member 1A	1.64

Probe Set ID	Accession_ID	Gene	Description	Fold
1420967_at	NM_181325	Slc25a15	solute carrier family 25 (mitochondrial carrier ornithine transporter), member 15	1.64
1437850_a_at	NM_001109745	Cnbp	cellular nucleic acid binding protein	1.64
1453299_a_at	NM_001123371		purine-nucleoside phosphorylase	1.64
1416633_a_at	NM_026635	Fam96a	family with sequence similarity 96, member A	1.65
1439463_x_at	NM_001111282		predicted gene 2710 /// high-mobility group (nonhistone chromosomal) protein 1-like 1 /// high mobility group box 1	1.65
1449211_at	NM_011794		bisphosphate 3'-nucleotidase 1 /// hypothetical LOC100504952	1.65
1449089_at	NM_173440	Nrip1	nuclear receptor interacting protein 1	1.65
1416345_at	NM_013898	Timm8a1	translocase of inner mitochondrial membrane 8 homolog a1 (yeast)	1.65
1423938_at	NM_145438	Llgl2	lethal giant larvae homolog 2 (Drosophila)	1.65
1425495_at	NM_001024846	Zfp62	zinc finger protein 62	1.65
1439266_a_at	NM_025901	Polr3k	polymerase (RNA) III (DNA directed) polypeptide K	1.65
1451236_at	NM_001164212	Rerg	RAS-like, estrogen-regulated, growth-inhibitor	1.65
1417374_at	NM_009447	Tuba4a	tubulin, alpha 4A	1.65
1452458_s_at	NM_001081406	Ppil5	peptidylprolyl isomerase (cyclophilin) like 5	1.65
1428869_at	NM_001039351	Nolc1	nucleolar and coiled-body phosphoprotein 1	1.65
1417884_at	NM_001029842	Slc16a6	solute carrier family 16 (monocarboxylic acid transporters), member 6	1.65
1433807_at	NM_175265		RIKEN cDNA 6720463M24 gene	1.65
1460392_a_at	NM_175009	Eny2	enhancer of yellow 2 homolog (Drosophila)	1.65
1423065_at	NM_007872	Dnmt3a	DNA methyltransferase 3A	1.65
1424342_at	NM_001159349	Fyttd1	forty-two-three domain containing 1	1.65
1433870_at	NM_146026	Prr15l	proline rich 15-like	1.65
1422580_at	NM_010858	Myl4	myosin, light polypeptide 4	1.65
1437318_at	NM_001195046	Pak3	p21 protein (Cdc42/Rac)-activated kinase 3	1.65
1429624_at	NM_025690	Sltm	SAFB-like, transcription modulator	1.65
1438287_x_at	NM_197982	Ddx39	DEAD (Asp-Glu-Ala-Asp) box polypeptide 39	1.65
1422483_a_at	NM_007808	Cycs	cytochrome c, somatic	1.65
1434031_at	NM_001040686	Zfp692-ps	zinc finger protein 692, pseudogene	1.65
1460474_at	C76835		RIKEN cDNA 2610028L16 gene	1.65
1425159_at	NM_026680	Golt1a	golgi transport 1 homolog A (S. cerevisiae)	1.66
1422573_at	NM_009667	Ampd3	adenosine monophosphate deaminase 3	1.66
1421784_a_at	NM_007910	Efna4	ephrin A4	1.66
1439966_x_at	NM_053196	Sfxn2	sideroflexin 2	1.66
1455496_at	NM_001159519	Pfas	phosphoribosylformylglycinamidine synthase (FGAR amidotransferase)	1.66
1431028_a_at	NM_001114339	Pank1	pantothenate kinase 1	1.66
1433827_at	NM_001038999	Atp8a1	ATPase, aminophospholipid transporter (APLT), class I, type 8A, member 1	1.66
1417395_at	NM_010637	Klf4	Kruppel-like factor 4 (gut)	1.66
1456653_a_at	NM_001170785	Mthfd1l	methylenetetrahydrofolate dehydrogenase (NADP+ dependent) 1-like	1.66
1415964_at	NM_009127	Scd1	stearoyl-Coenzyme A desaturase 1	1.66
1438984_x_at	NM_008945	Psmb4	proteasome (prosome, macropain) subunit, beta type 4	1.66
1434815_a_at	NM_178907	Mapkapk3	mitogen-activated protein kinase-activated protein kinase 3	1.66
1437187_at	NM_178609	E2f7	E2F transcription factor 7	1.66
1416748_a_at	NM_018736	Mre11a	meiotic recombination 11 homolog A (S. cerevisiae)	1.66
1429169_at	NM_001166409	Rbm3	RNA binding motif protein 3	1.66
1418036_at	NM_008922	Prim2	DNA primase, p58 subunit	1.66
1415909_at	NM_016737	Stip1	stress-induced phosphoprotein 1	1.66
1415903_at	NM_001166456	Slc38a1	solute carrier family 38, member 1	1.66
1416595_at	NM_025485	Mrps22	mitochondrial ribosomal protein S22	1.66
1428409_at	NM_028108	Naa50	N(alpha)-acetyltransferase 50, NatE catalytic subunit	1.66

Probe Set ID	Accession_ID	Gene	Description	Fold
1415772_at	NM_010880	Ncl	nucleolin	1.66
1426908_at	NM_001167981	Galnt7	UDP-N-acetyl-alpha-D-galactosamine: polypeptide N-acetylgalactosaminyltransferase 7	1.67
1429093_at	NM_001017966	Ddi2	DNA-damage inducible protein 2	1.67
1436472_at	NM_172796	Slfn9	schlafen 9	1.67
1454659_at	NM_001161515	Dctd	dCMP deaminase	1.67
1459984_at	NM_177389	Mia3	melanoma inhibitory activity 3	1.67
1456865_x_at	NM_021511	Rrs1	RRS1 ribosome biogenesis regulator homolog (S. cerevisiae)	1.67
1423184_at	NM_011365	Itsn2	intersectin 2	1.67
1437611_x_at	NM_134471	Kif2c	kinesin family member 2C	1.67
1417797_a_at	NM_001083916		RIKEN cDNA 1810019J16 gene	1.67
1419038_a_at	NM_007788	Csnk2a1	casein kinase 2, alpha 1 polypeptide	1.67
1439695_a_at	NM_183046	Kif20b	kinesin family member 20B	1.67
1423249_at	NM_010918	Nktr	natural killer tumor recognition sequence	1.67
1423295_at	NM_080556	Tm9sf2	transmembrane 9 superfamily member 2	1.67
1452812_at	NM_181039	Lphn1	latrophilin 1	1.67
1427253_s_at	NM_001163018	Suz12	suppressor of zeste 12 homolog (Drosophila)	1.67
1433530_at	NM_018860	Rpl41	ribosomal protein L41	1.67
1416605_at	NM_026631	Nhp2	NHP2 ribonucleoprotein homolog (yeast)	1.67
1430981_s_at	NM_001122963	Gpbp1	GC-rich promoter binding protein 1	1.67
1428105_at	NM_001141975	Tpx2	TPX2, microtubule-associated protein homolog (Xenopus laevis)	1.67
1448326_a_at	NM_013496	Crabp1	cellular retinoic acid binding protein I	1.67
1425199_a_at	NM_001113416	Epb4.1l5	erythrocyte protein band 4.1-like 5	1.67
1433623_at	NM_175494	Zfp367	zinc finger protein 367	1.67
1424156_at	NM_001139516	Rbl1	retinoblastoma-like 1 (p107)	1.67
1419943_s_at	NM_172301	Ccnb1	cyclin B1	1.67
1455951_at	NM_001003913	Mars	methionine-tRNA synthetase	1.67
1416687_at	NM_001142916	Plod2	procollagen lysine, 2-oxoglutarate 5-dioxygenase 2	1.68
1416014_at	NM_015751	Abce1	ATP-binding cassette, sub-family E (OABP), member 1	1.68
1428483_a_at	NM_025642		RIKEN cDNA 2610039C10 gene	1.68
1452158_at	NM_029735	Eprs	glutamyl-prolyl-tRNA synthetase	1.68
1419173_at	NM_025371	Acy1	aminoacylase 1	1.68
1426366_at	NM_153178	Eif2c2	eukaryotic translation initiation factor 2C, 2	1.68
1448612_at	NM_018754	Sfn	stratifin	1.68
1424436_at	NM_010256	Gart	phosphoribosylglycinamide formyltransferase	1.68
1426580_at	NM_011495	Plk4	polo-like kinase 4 (Drosophila)	1.68
1422016_a_at	NM_021886	Cenph	centromere protein H	1.68
1420518_a_at	NM_001145800	Igsf9	immunoglobulin superfamily, member 9	1.68
1429294_at	NM_027182	Trip13	thyroid hormone receptor interactor 13	1.68
1417910_at	NM_009828	Ccna2	cyclin A2	1.68
1426790_at	NM_001136081	Ssrp1	structure specific recognition protein 1	1.68
1456197_x_at	NM_001099299	Ajap1	Adherens junction associated protein 1	1.68
1427126_at	NM_010478	Hspa1b	heat shock protein 1B	1.68
1455988_a_at	NM_009838	Cct6a	chaperonin containing Tcp1, subunit 6a (zeta)	1.68
1418656_at	NM_025520	Lsm5	LSM5 homolog, U6 small nuclear RNA associated (S. cerevisiae)	1.68
1419427_at	NM_009971	Csf3	colony stimulating factor 3 (granulocyte)	1.68
1448127_at	NM_009103	Rrm1	ribonucleotide reductase M1	1.68
1435504_at	NM_030179	Clip4	CAP-GLY domain containing linker protein family, member 4	1.68
1454011_a_at	NM_011284	Rpa2	replication protein A2	1.69
1429057_at	NM_025832	Naa16	N(alpha)-acetyltransferase 16, NatA auxiliary subunit	1.69
1427997_at	NM_026742	Ndufaf4	NADH dehydrogenase (ubiquinone) 1 alpha subcomplex, assembly factor 4	1.69
1433680_x_at	NM_001161737	Siva1	SIVA1, apoptosis-inducing factor	1.69

Probe Set ID	Accession_ID	Gene	Description	Fold
1456003_a_at	NM_018861	Slc1a4	solute carrier family 1 (glutamate/neutral amino acid transporter), member 4	1.69
1417132_at	NM_007658	Cdc25a	cell division cycle 25 homolog A (S. pombe)	1.69
1435989_x_at	NM_031170	Krt8	keratin 8	1.69
1448780_at	NM_009194	Slc12a2	solute carrier family 12, member 2	1.69
1455299_at	NM_028572	Vgll3	vestigial like 3 (Drosophila)	1.69
1436574_at	NM_027285		RIKEN cDNA 1700029I01 gene	1.69
1442454_at	NM_011623	Top2a	topoisomerase (DNA) II alpha	1.69
1435936_at	NM_001004148	Slc13a5	solute carrier family 13 (sodium-dependent citrate transporter), member 5	1.69
1460353_at	NM_028355	Tmem48	transmembrane protein 48	1.69
1424858_at	NM_145443	L2hgdh	L-2-hydroxyglutarate dehydrogenase	1.69
1418825_at	NM_008326	Irgm1	immunity-related GTPase family M member 1	1.70
1450886_at	NM_010353	Gsg2	germ cell-specific gene 2	1.70
1460391_at	NM_025942	Ola1	Obg-like ATPase 1	1.70
1438116_x_at	NM_012030	Slc9a3r1	solute carrier family 9 (sodium/hydrogen exchanger), member 3 regulator 1	1.70
1454664_a_at	NM_173363	Eif5	eukaryotic translation initiation factor 5	1.70
1427837_at	U25103	Igk-V32	Immunoglobulin kappa chain variable 32 (V32)	1.70
1426854_a_at	NM_023871	Set	SET nuclear oncogene	1.70
1434700_at	NM_001015099	G2e3	G2/M-phase specific E3 ubiquitin ligase	1.70
1460684_at	NM_028454	Tm7sf2	transmembrane 7 superfamily member 2	1.70
1426147_s_at	NM_001160096	Cldn10	claudin 10	1.70
1424794_at	NM_025786	Rnf186	ring finger protein 186	1.70
1443964_at	NM_146260	Tmie	transmembrane inner ear	1.70
1418030_at	NM_001038643	Slco3a1	solute carrier organic anion transporter family, member 3a1	1.70
1434940_x_at	NM_026446	Rgs19	regulator of G-protein signaling 19	1.70
1450448_at	NM_009285	Stc1	stanniocalcin 1	1.70
1428837_at	NM_001081403	Klhl14	kelch-like 14 (Drosophila)	1.70
1437466_at	NM_009655	Alcam	activated leukocyte cell adhesion molecule	1.71
1437164_x_at	NM_138597	Atp5o	ATP synthase, H+ transporting, mitochondrial F1 complex, O subunit	1.71
1438945_x_at	NM_010288	Gja1	gap junction protein, alpha 1	1.71
1417957_a_at	NM_133681	Tspan1	tetraspanin 1	1.71
1417994_a_at	NM_001034013	Accn1	amiloride-sensitive cation channel 1, neuronal (degenerin)	1.71
1434643_at	NM_020601	Tbl1x	transducin (beta)-like 1 X-linked	1.71
1441429_at	NM_010572	Irs4	insulin receptor substrate 4	1.71
1435308_at	NM_010243	Fut9	fucosyltransferase 9	1.71
1419662_at	NM_008760	Ogn	osteoglycin	1.71
1419397_at	NM_008892	Pola1	polymerase (DNA directed), alpha 1	1.71
1426300_at	NM_009655	Alcam	activated leukocyte cell adhesion molecule	1.71
1437638_at	NM_175229	Srrm2	serine/arginine repetitive matrix 2	1.71
1460092_at	BB082567	---		1.71
1436707_x_at	NM_144818	Ncaph	non-SMC condensin I complex, subunit H	1.71
1426081_a_at	NM_010050	Dio2	deiodinase, iodothyronine, type II	1.71
1426485_at	NM_026390	Ubxn4	UBX domain protein 4	1.71
1447100_s_at	NM_027482		RIKEN cDNA 5730508B09 gene	1.71
1415811_at	NM_001111078	Uhrf1	ubiquitin-like, containing PHD and RING finger domains, 1	1.71
1454688_x_at	NM_026775	Tmed10	transmembrane emp24-like trafficking protein 10 (yeast)	1.71
1435452_at	NM_175507	Tmem20	transmembrane protein 20	1.71
1416346_at	NM_013898	Timm8a1	translocase of inner mitochondrial membrane 8 homolog a1 (yeast)	1.71
1417947_at	NM_011045	Pcna	proliferating cell nuclear antigen	1.72
1424300_at	NM_026053	Gemin6	gem (nuclear organelle) associated protein 6	1.72
1450496_a_at	NM_001164355	Ska1	spindle and kinetochore associated complex subunit 1	1.72

Probe Set ID	Accession_ID	Gene	Description	Fold
1418818_at	NM_009701	Aqp5	aquaporin 5	1.72
1423241_a_at	NM_009361	Tfdp1	transcription factor Dp 1	1.72
1448507_at	NM_028889	Efhd1	EF hand domain containing 1	1.72
1439065_x_at	NM_001039209	Gm13152	predicted gene 13152	1.72
1418488_s_at	NM_023663	Ripk4	receptor-interacting serine-threonine kinase 4	1.72
1449009_at	NM_001145164		T-cell specific GTPase 1	1.72
1460049_s_at	NM_024283		RIKEN cDNA 1500015O10 gene	1.72
1419639_at	NM_010111	Efnb2	ephrin B2	1.72
1438018_at	NM_030014	Hook1	hook homolog 1 (Drosophila)	1.72
1418018_at	NM_007754	Cpd	carboxypeptidase D	1.72
1425272_at	NM_007929	Emp2	epithelial membrane protein 2	1.72
1429436_at	NM_018785	Prpf40a	PRP40 pre-mRNA processing factor 40 homolog A (yeast)	1.72
1448191_at	NM_011121	Plk1	polo-like kinase 1 (Drosophila)	1.72
1441910_x_at	NM_007633	Ccne1	cyclin E1	1.72
1417394_at	NM_010637	Klf4	Kruppel-like factor 4 (gut)	1.72
1416568_a_at	NM_001085472	Acin1	apoptotic chromatin condensation inducer 1	1.72
1448277_at	NM_008894	Pold2	polymerase (DNA directed), delta 2, regulatory subunit	1.73
1456054_a_at	NM_001159603	Pum1	pumilio 1 (Drosophila)	1.73
1420008_s_at	NM_170779	Wwc1	WW, C2 and coiled-coil domain containing 1	1.73
1459894_at	NM_027711	Iqgap2	IQ motif containing GTPase activating protein 2	1.73
1452902_at	NM_183286	Dhrs13	dehydrogenase/reductase (SDR family) member 13	1.73
1433683_at	NM_176838	Esrp2	epithelial splicing regulatory protein 2	1.73
1452397_at	NM_026054		RIKEN cDNA 2810474O19 gene	1.73
1435155_at	NM_001037711	Cgn	cingulin	1.73
1456698_s_at	NM_016690	Hnrpdl	heterogeneous nuclear ribonucleoprotein D-like	1.73
1417867_at	NM_013459	Cfd	complement factor D (adipsin)	1.73
1429520_a_at	NM_025408	Acer3	alkaline ceramidase 3	1.73
1451967_x_at	NM_008379	Kpnb1	karyopherin (importin) beta 1	1.73
1423381_at	NM_027582		RIKEN cDNA 4921521F21 gene	1.74
1419254_at	NM_008638	Mthfd2	methylenetetrahydrofolate dehydrogenase (NAD+ dependent), methenyltetrahydrofolate cyclohydrolase	1.74
1423775_s_at	NM_145150	Prc1	protein regulator of cytokinesis 1	1.74
1457281_at	NM_030046	Dnajc21	DnaJ (Hsp40) homolog, subfamily C, member 21	1.74
1420937_at	NM_016856	Cpsf2	cleavage and polyadenylation specific factor 2	1.74
1426118_a_at	NM_001109748	Tomm40	translocase of outer mitochondrial membrane 40 homolog (yeast)	1.74
1440635_at	NM_001081390	Palld	palladin, cytoskeletal associated protein	1.74
1438902_a_at	NM_010480	Hsp90aa1	heat shock protein 90, alpha (cytosolic), class A member 1	1.74
1452598_at	NM_001163476	Gins1	GINS complex subunit 1 (Psf1 homolog)	1.74
1418203_at	NM_021451	Pmaip1	phorbol-12-myristate-13-acetate-induced protein 1	1.75
1434239_at	NM_199447	Rrp12	ribosomal RNA processing 12 homolog (S. cerevisiae)	1.75
1430982_at	NM_001078167	Srsf1	serine/arginine-rich splicing factor 1	1.75
1448595_a_at	NM_009052	Bex1	brain expressed gene 1	1.75
1425993_a_at	NM_013559	Hsph1	heat shock 105kDa/110kDa protein 1	1.75
1431609_a_at	NM_001102404	Acp5	acid phosphatase 5, tartrate resistant	1.75
1455771_at	NM_172449	Bzrap1	benzodiazapine receptor associated protein 1	1.75
1428480_at	NM_026560	Cdca8	cell division cycle associated 8	1.75
1418760_at	NM_021557	Rdh11	retinol dehydrogenase 11	1.75
1452318_a_at	NM_010478	Hspa1b	heat shock protein 1B	1.75
1417144_at	NM_134024	Tubg1	tubulin, gamma 1	1.75
1436600_at	NM_172913	Tox3	TOX high mobility group box family member 3	1.75
1439526_at	AV375160			1.75
1454671_at	NM_153526	Insig1	insulin induced gene 1	1.76
1418430_at	NM_008448	Kif5b	kinesin family member 5B	1.76

Probe Set ID	Accession_ID	Gene	Description	Fold
1455832_a_at	NM_009471	Umps	uridine monophosphate synthetase	1.76
1452115_a_at	NM_011495	Plk4	polo-like kinase 4 (Drosophila)	1.76
1443733_x_at	NM_133692	Pold3	polymerase (DNA-directed), delta 3, accessory subunit	1.76
1455818_at	NM_134041		RIKEN cDNA 4930427A07 gene	1.76
1423809_at	NM_001163763	Tcf19	transcription factor 19	1.76
1434291_a_at	NM_011353	Serf1	small EDRK-rich factor 1	1.76
1420647_a_at	NM_031170	Krt8	keratin 8	1.76
1423691_x_at	NM_031170	Krt8	keratin 8	1.77
1416632_at	NM_008615	Me1	malic enzyme 1, NADP(+)-dependent, cytosolic	1.77
1449799_s_at	NM_026163	Pkp2	plakophilin 2	1.77
1434605_at	NM_198303	Eif5b	eukaryotic translation initiation factor 5B	1.77
1428333_at	NM_029823		RIKEN cDNA 2900062L11 gene	1.77
1416336_s_at	NM_009226	Snrpd1	small nuclear ribonucleoprotein D1	1.77
1449061_a_at	NM_008921	Prim1	DNA primase, p49 subunit	1.77
1460193_at	NM_133726	St13	suppression of tumorigenicity 13	1.77
1448820_a_at	NM_026030	Eif2s2	eukaryotic translation initiation factor 2, subunit 2 (beta)	1.77
1421546_a_at	NM_012025	Racgap1	Rac GTPase-activating protein 1	1.77
1445186_at	NM_011491	Stc2	stanniocalcin 2	1.77
1420917_at	NM_018785	Prpf40a	PRP40 pre-mRNA processing factor 40 homolog A (yeast)	1.77
1439270_x_at	NM_009391	Ran	RAN, member RAS oncogene family	1.77
1421912_at	NM_011397	Slc23a1	solute carrier family 23 (nucleobase transporters), member 1	1.77
1439415_x_at	NM_025587	Rps21	ribosomal protein S21	1.77
1437552_at	NM_026120		RIKEN cDNA 2410127L17 gene	1.77
1418105_at	NM_019675	Stmn4	stathmin-like 4	1.77
1452583_s_at	NM_176963	Galm	galactose mutarotase	1.77
1415917_at	NM_138745	Mthfd1	methylenetetrahydrofolate dehydrogenase (NADP+ dependent), methenyltetrahydrofolate cyclohydrolase, formyltetrahydrofolate synthase	1.77
1416166_a_at	NM_016764	Prdx4	peroxiredoxin 4	1.77
1437186_at	NM_201364	BC055324	cDNA sequence BC055324	1.77
1417398_at	NM_025846	Rras2	related RAS viral (r-ras) oncogene homolog 2	1.77
1424143_a_at	NM_026014	Cdt1	chromatin licensing and DNA replication factor 1	1.78
1449042_at	NM_181322	Ctcf	CCCTC-binding factor	1.78
1416235_at	NM_133807	Lrrc59	leucine rich repeat containing 59	1.78
1418025_at	NM_011498	Bhlhe40	basic helix-loop-helix family, member e40	1.78
1452298_a_at	NM_201600	Myo5b	myosin VB	1.78
1420843_at	NM_011213	Ptprf	protein tyrosine phosphatase, receptor type, F	1.78
1416222_at	NM_010941	Nsdhl	NAD(P) dependent steroid dehydrogenase-like	1.78
1417926_at	NM_133762	Ncapg2	non-SMC condensin II complex, subunit G2	1.78
1447166_at	AI891479			1.78
1433862_at	NM_001014976	Espl1	extra spindle poles-like 1 (S. cerevisiae)	1.78
1426645_at	NM_010480	Hsp90aa1	heat shock protein 90, alpha (cytosolic), class A member 1	1.78
1451346_at	NM_024433	Mtap	methylthioadenosine phosphorylase	1.79
1445499_at	NM_026083	Zc3h13	zinc finger CCCH type containing 13	1.79
1418469_at	NM_173440	Nrip1	nuclear receptor interacting protein 1	1.79
1460081_at	NM_018801	Syt7	synaptotagmin VII	1.79
1429270_a_at	NM_001168244	Syce2	synaptonemal complex central element protein 2	1.79
1417131_at	NM_007658	Cdc25a	cell division cycle 25 homolog A (S. pombe)	1.79
1455724_at	NM_001164275	Prrg1	proline rich Gla (G-carboxyglutamic acid) 1	1.79
1446957_s_at	NM_030563	N4bp1	NEDD4 binding protein 1	1.79
1426569_a_at	NM_001159544	Frk	fyn-related kinase	1.79
1423525_at	NM_025979	Mastl	microtubule associated serine/threonine kinase-like	1.79
1448673_at	NM_021495	Pvrl3	poliovirus receptor-related 3	1.79

Probe Set ID	Accession_ID	Gene	Description	Fold
1437780_at	NM_001146081	Fancb	Fanconi anemia, complementation group B	1.79
1449207_a_at	NM_001166406	Kif20a	kinesin family member 20A	1.79
1435368_a_at	NM_007415	Parp1	poly (ADP-ribose) polymerase family, member 1	1.80
1438487_s_at	NM_001080755	Zzz3	zinc finger, ZZ domain containing 3	1.80
1452713_a_at	NM_025645	Snrnp40	small nuclear ribonucleoprotein 40 (U5)	1.80
1449863_a_at	NM_010056	Dlx5	distal-less homeobox 5	1.80
1432016_a_at	NM_029573	Idh3a	isocitrate dehydrogenase 3 (NAD+) alpha	1.80
1423569_at	NM_025961	Gatm	glycine amidinotransferase (L-arginine:glycine amidinotransferase)	1.80
1448182_a_at	NM_009846	Cd24a	CD24a antigen	1.80
1424511_at	NM_011497	Aurka	aurora kinase A	1.80
1433813_at	NM_028355	Tmem48	transmembrane protein 48	1.80
1452378_at	NR_002847	Malat1	metastasis associated lung adenocarcinoma transcript 1 (non-coding RNA)	1.80
1444112_at	XR_104848	Gm15417	predicted gene 15417	1.80
1448640_at	NM_001171010	Slc14a1	solute carrier family 14 (urea transporter), member 1	1.80
1424652_at	NM_145570	Fam176a	family with sequence similarity 176, member A	1.80
1420985_at	NM_138679	Ash1l	ash1 (absent, small, or homeotic)-like (Drosophila)	1.81
1421262_at	NM_010720	Lipg	lipase, endothelial	1.81
1452118_at	NM_001163734	Rrp1b	ribosomal RNA processing 1 homolog B (S. cerevisiae)	1.81
1423060_at	NM_011119	Pa2g4	proliferation-associated 2G4	1.81
1418216_at	NM_011820	Ggt5	gamma-glutamyltransferase 5	1.81
1443736_at	BG868839	Gm13648	predicted gene 13648	1.81
1456120_at	NM_177608	Secisbp2l	SECIS binding protein 2-like	1.81
1424110_a_at	NM_008704	Nme1	non-metastatic cells 1, protein (NM23A) expressed in	1.81
1423774_a_at	NM_145150	Prc1	protein regulator of cytokinesis 1	1.81
1417403_at	NM_130450	Elovl6	ELOVL family member 6, elongation of long chain fatty acids (yeast)	1.81
1427090_at	NM_181412	Zbed4	zinc finger, BED domain containing 4	1.82
1429171_a_at	NM_019438	Ncapg	non-SMC condensin I complex, subunit G	1.82
1438697_at	NM_175432	Tmem132c	transmembrane protein 132C	1.82
1427257_at	NM_001081249	Vcan	versican	1.82
1449476_at	NM_011973	Stk30	serine/threonine kinase 30	1.82
1416468_at	NM_013467	Aldh1a1	aldehyde dehydrogenase family 1, subfamily A1	1.82
1429239_a_at	NM_133774	Stard4	StAR-related lipid transfer (START) domain containing 4	1.82
1418189_s_at	NR_002847	Malat1	metastasis associated lung adenocarcinoma transcript 1 (non-coding RNA)	1.82
1418188_a_at	NR_002847	Malat1	metastasis associated lung adenocarcinoma transcript 1 (non-coding RNA)	1.82
1448205_at	NM_172301	Ccnb1	cyclin B1	1.82
1452907_at	NM_008079	Galc	galactosylceramidase	1.82
1452241_at	NM_176979	Topbp1	topoisomerase (DNA) II binding protein 1	1.82
1431293_a_at	NM_171826	Cldnd1	claudin domain containing 1	1.82
1438556_a_at	NM_016963	Tmod3	tropomodulin 3	1.82
1419555_at	NM_001145813	Elf5	E74-like factor 5	1.82
1422462_at	NM_026024	Ube2t	ubiquitin-conjugating enzyme E2T (putative)	1.82
1438403_s_at	NR_002847	Malat1	metastasis associated lung adenocarcinoma transcript 1 (non-coding RNA)	1.83
1454658_at	NM_173751	Ilvbl	ilvB (bacterial acetolactate synthase)-like	1.83
1452314_at	NM_010615	Kif11	kinesin family member 11	1.83
1428061_at	NM_026115	Hat1	histone aminotransferase 1	1.83
1426327_s_at	NM_053009		ciliary neurotrophic factor	1.83
1449877_s_at	NM_001195298	Kifc1	kinesin family member C1	1.83
1454904_at	NM_001164190	Mtm1	X-linked myotubular myopathy gene 1	1.83
1424966_at	NM_001168256	Tmem40	transmembrane protein 40	1.83

Probe Set ID	Accession_ID	Gene	Description	Fold
1416858_a_at	NM_013902	Fkbp3	FK506 binding protein 3	1.83
1451424_at	NM_146017	Gabrp	gamma-aminobutyric acid (GABA) A receptor, pi	1.83
1453128_at	NM_053247	Lyve1	lymphatic vessel endothelial hyaluronan receptor 1	1.83
1439016_x_at	NM_001164787		small proline-rich protein 2A1	1.83
1456542_s_at	NM_001081054	Qrsl1	glutaminyl-tRNA synthase (glutamine-hydrolyzing)-like 1	1.83
1437467_at	NM_009655	Alcam	activated leukocyte cell adhesion molecule	1.83
1455528_at	AI315686			1.83
1421955_a_at	NM_010890	Nedd4	neural precursor cell expressed, developmentally down-regulated 4	1.83
1426047_a_at	NM_001161838	Ptprr	protein tyrosine phosphatase, receptor type, R	1.83
1433866_x_at	NM_011034	Prdx1	peroxiredoxin 1	1.84
1447927_at	NM_001039646		guanylate-binding protein 10 /// macrophage activation 2 like	1.84
1453796_a_at	NM_026168	Ergic2	ERGIC and golgi 2	1.84
1422484_at	NM_007808	Cycs	cytochrome c, somatic	1.84
1417057_a_at	NM_026352		lysosomal-associated membrane protein 3	1.84
1417689_a_at	NM_001164557	Pdzk1ip1	PDZK1 interacting protein 1	1.84
1452414_s_at	NM_023731	Ccdc86	coiled-coil domain containing 86	1.84
1424971_at	NM_027411	Ccdc99	coiled-coil domain containing 99	1.84
1418715_at	NM_001114339	Pank1	pantothenate kinase 1	1.84
1423601_s_at	NM_011552	Tcof1	Treacher Collins Franceschetti syndrome 1, homolog	1.85
1451481_s_at	NM_145932	Osta	organic solute transporter alpha	1.85
1455959_s_at	NM_010295	Gclc	glutamate-cysteine ligase, catalytic subunit	1.85
1451667_at	NM_145413	Fam20b	family with sequence similarity 20, member B	1.85
1435082_at	NM_013635	Sypl	synaptophysin-like protein	1.85
1439040_at	NM_173762	Cenpe	centromere protein E	1.85
1427893_a_at	NM_026784	Pmvk	phosphomevalonate kinase	1.85
1429379_at	NM_053247	Lyve1	lymphatic vessel endothelial hyaluronan receptor 1	1.85
1429240_at	NM_133774	Stard4	StAR-related lipid transfer (START) domain containing 4	1.85
1426744_at	NM_033218	Srebf2	sterol regulatory element binding factor 2	1.85
1457867_at	NM_001004173	Sgpp2	sphingosine-1-phosphate phosphotase 2	1.85
1420622_a_at	NM_031165	Hspa8	heat shock protein 8	1.85
1435181_at	NM_001115010	Lin54	lin-54 homolog (C. elegans)	1.85
1457913_at	NM_001082485		RIKEN cDNA 5730601F06 gene	1.85
1452047_at	NM_009786	Cacybp	calcyclin binding protein	1.85
1436390_a_at	NM_001177770	Clcc1	chloride channel CLIC-like 1	1.86
1448441_at	NM_016904	Cks1b	CDC28 protein kinase 1b	1.86
1418527_a_at	NM_001080387	Srsf10	serine/arginine-rich splicing factor 10	1.86
1448885_at	NM_028712	Rap2b	RAP2B, member of RAS oncogene family	1.86
1417028_a_at	NM_030706	Trim2	tripartite motif-containing 2	1.86
1448844_at	NM_025558	Cyb5b	cytochrome b5 type B	1.86
1454952_s_at	NM_178113	Ncapd3	non-SMC condensin II complex, subunit D3	1.86
1423092_at	NM_016692	Incenp	inner centromere protein	1.86
1440785_at	NM_212452	Rxfp1	relaxin/insulin-like family peptide receptor 1	1.86
1451033_a_at	NM_016984	Trpc4	transient receptor potential cation channel, subfamily C, member 4	1.86
1437327_x_at	NM_001163035	Enoph1	enolase-phosphatase 1	1.86
1429024_at	NM_001170847	Rbm20	RNA binding motif protein 20	1.86
1421694_a_at	NM_001081249	Vcan	versican	1.86
1448551_a_at	NM_030706	Trim2	tripartite motif-containing 2	1.86
1427275_at	NM_133786	Smc4	structural maintenance of chromosomes 4	1.86
1416059_at	NM_019787	Sec23b	SEC23B (S. cerevisiae)	1.86
1456071_a_at	NM_007808	Cycs	cytochrome c, somatic	1.86
1417351_a_at	NM_021336	Snrpa1	small nuclear ribonucleoprotein polypeptide A'	1.87
1449699_s_at	NM_172616		RIKEN cDNA C330027C09 gene	1.87

Probe Set ID	Accession_ID	Gene	Description	Fold
1449167_at	NM_013512	Epb4.1l4a	erythrocyte protein band 4.1-like 4a	1.87
1439753_x_at	NM_011382	Six4	sine oculis-related homeobox 4 homolog (Drosophila)	1.87
1416041_at	NM_001161845	Sgk1	serum/glucocorticoid regulated kinase 1	1.87
1430811_a_at	NM_023284	Nuf2	NUF2, NDC80 kinetochore complex component, homolog (S. cerevisiae)	1.87
1453684_s_at	NM_026934	Zc3h15	zinc finger CCCH-type containing 15	1.87
1452954_at	NM_026785	Ube2c	ubiquitin-conjugating enzyme E2C	1.87
1438567_at	NM_172840	Vwa2	von Willebrand factor A domain containing 2	1.87
1416544_at	NM_001146689	Ezh2	enhancer of zeste homolog 2 (Drosophila)	1.87
1416042_s_at	NM_001081475	Nasp	nuclear autoantigenic sperm protein (histone-binding)	1.87
1416835_s_at	NM_009665	Amd1	S-adenosylmethionine decarboxylase 1	1.87
1436791_at	NM_009524	Wnt5a	wingless-related MMTV integration site 5A	1.88
1428951_at	NM_001081350	Nol8	nucleolar protein 8	1.88
1448635_at	NM_008017	Smc2	structural maintenance of chromosomes 2	1.88
1437033_a_at	NM_013787	Skp2	S-phase kinase-associated protein 2 (p45)	1.88
1419838_s_at	NM_011495	Plk4	polo-like kinase 4 (Drosophila)	1.88
1416501_at	NM_001080773	Pdpk1	3-phosphoinositide dependent protein kinase 1	1.89
1427127_x_at	NM_010478	Hspa1b	heat shock protein 1B	1.89
1436981_a_at	NM_011740		tyrosine 3-monooxygenase/tryptophan 5-monooxygenase activation protein, zeta polypeptide	1.89
1416939_at	NM_026438	Ppa1	pyrophosphatase (inorganic) 1	1.89
1433531_at	NM_001033600	Acsl4	acyl-CoA synthetase long-chain family member 4	1.90
1434020_at	NM_001033313	Pdap1	PDGFA associated protein 1	1.90
1448466_at	NM_026410	Cdca5	cell division cycle associated 5	1.90
1454903_at	NM_033217	Ngfr	nerve growth factor receptor (TNFR superfamily, member 16)	1.90
1424507_at	NM_145495	Rin1	Ras and Rab interactor 1	1.90
1416214_at	NM_008565	Mcm4	minichromosome maintenance deficient 4 homolog (S. cerevisiae)	1.90
1447930_at	NM_013815		bromodomain adjacent to zinc finger domain 1A	1.90
1429658_a_at	NM_008017	Smc2	structural maintenance of chromosomes 2	1.90
1416046_a_at	NM_025799	Fuca2	fucosidase, alpha-L- 2, plasma	1.91
1449357_at	NM_025865		RIKEN cDNA 2310030G06 gene	1.91
1429499_at	NM_025995	Fbxo5	F-box protein 5	1.91
1424768_at	NM_145575	Cald1	caldesmon 1	1.91
1422798_at	NM_001004357	Cntnap2	contactin associated protein-like 2	1.91
1422651_at	NM_009605	Adipoq	adiponectin, C1Q and collagen domain containing	1.91
1427231_at	NM_019413	Robo1	roundabout homolog 1 (Drosophila)	1.91
1449855_s_at	NM_016723		ubiquitin carboxyl-terminal esterase L3 (ubiquitin thiolesterase)	1.91
1448261_at	NM_009864	Cdh1	cadherin 1	1.91
1429411_a_at	NM_175009	Eny2	enhancer of yellow 2 homolog (Drosophila)	1.91
1424766_at	NM_146235	Ercc6l	excision repair cross-complementing rodent repair deficiency complementation group 6 - like	1.91
1452885_at	NM_028148	Srsf2ip	serine/arginine-rich splicing factor 2, interacting protein	1.91
1453321_at	NM_001081416	Fndc1	fibronectin type III domain containing 1	1.91
1459299_at	NM_177376	Myo3b	myosin IIIB	1.92
1422767_at	NM_016859	Bysl	bystin-like	1.92
1458374_at	NM_172578	C79407	expressed sequence C79407	1.92
1460247_a_at	NM_013787	Skp2	S-phase kinase-associated protein 2 (p45)	1.92
1448918_at	NM_001038643	Slco3a1	solute carrier organic anion transporter family, member 3a1	1.92
1418969_at	NM_013787	Skp2	S-phase kinase-associated protein 2 (p45)	1.92
1449615_s_at	NM_133808	Hdlbp	high density lipoprotein (HDL) binding protein	1.92
1453462_at	NM_027928	Chst13	carbohydrate (chondroitin 4) sulfotransferase 13	1.92
1450862_at	NM_001122958	Rad54l	RAD54 like (S. cerevisiae)	1.92
1449140_at	NM_026023	Nudcd2	NudC domain containing 2	1.92

Probe Set ID	Accession_ID	Gene	Description	Fold
1433965_at	NM_001038999	Atp8a1	ATPase, aminophospholipid transporter (APLT), class I, type 8A, member 1	1.92
1450131_a_at	NM_138653	Bspry	B-box and SPRY domain containing	1.92
1456487_at	NM_009622	Adcy1	adenylate cyclase 1	1.92
1424128_x_at	NM_011496	Aurkb	aurora kinase B	1.92
1452360_a_at	NM_145997	Kdm5a	lysine (K)-specific demethylase 5A	1.92
1418026_at	NM_012012	Exo1	exonuclease 1	1.93
1428892_at	NM_026845	Ppil1	peptidylprolyl isomerase (cyclophilin)-like 1	1.93
1438702_at	NM_201518	Flrt2	fibronectin leucine rich transmembrane protein 2	1.93
1423544_at	NM_001163565	Ptpn5	protein tyrosine phosphatase, non-receptor type 5	1.93
1416698_a_at	NM_016904	Cks1b	CDC28 protein kinase 1b	1.93
1424495_a_at	NM_001037841	Cklf	chemokine-like factor	1.93
1452040_a_at	NM_013538	Cdca3	cell division cycle associated 3	1.93
1430139_at	NM_008234	Hells	helicase, lymphoid specific	1.94
1420683_at	NM_001168356	Bnipl	BCL2/adenovirus E1B 19kD interacting protein like	1.94
1448314_at	NM_007659	Cdk1	cyclin-dependent kinase 1	1.94
1426371_at	NM_026143	Far1	fatty acyl CoA reductase 1	1.94
1422293_a_at	NM_001142731	Kctd1	potassium channel tetramerisation domain containing 1	1.94
1419195_at	NM_001045554	Wfdc15b	WAP four-disulfide core domain 15B	1.94
1454799_at	NM_172715	Agpat9	1-acylglycerol-3-phosphate O-acyltransferase 9	1.94
1425544_at	NM_144920	Plekha5	pleckstrin homology domain containing, family A member 5	1.94
1433446_at	NM_145942	Hmgcs1	3-hydroxy-3-methylglutaryl-Coenzyme A synthase 1	1.94
1452358_at	NM_001103367	Rai2	retinoic acid induced 2	1.94
1415860_at	NM_010655	Kpna2	karyopherin (importin) alpha 2	1.94
1455938_x_at	NM_009009	Rad21	RAD21 homolog (S. pombe)	1.94
1455905_at	NM_001002004		RIKEN cDNA 2610507B11 gene	1.95
1423194_at	NM_009706	Arhgap5	Rho GTPase activating protein 5	1.95
1455426_at	NM_010140	Epha3	Eph receptor A3	1.95
1416311_s_at	NM_009446		tubulin, alpha 3A /// tubulin, alpha 3B	1.95
1456102_a_at	NM_001161618	Cul5	cullin 5	1.95
1456280_at	NM_175554	Clspn	claspin homolog (Xenopus laevis)	1.95
1439012_a_at	NM_007832	Dck	deoxycytidine kinase	1.95
1418856_a_at	NM_016925	Fanca	Fanconi anemia, complementation group A	1.95
1418334_at	NM_001190717	Dbf4	DBF4 homolog (S. cerevisiae)	1.95
1419270_a_at	NM_001159646	Dut	deoxyuridine triphosphatase	1.95
1455477_s_at	NM_001164557	Pdzk1ip1	PDZK1 interacting protein 1	1.95
1436000_a_at	NM_013787	Skp2	S-phase kinase-associated protein 2 (p45)	1.95
1452291_at	NM_178407	Arap2	ArfGAP with RhoGAP domain, ankyrin repeat and PH domain 2	1.95
1416021_a_at	NM_010634		fatty acid binding protein 5, epidermal /// predicted gene 3601	1.95
1418320_at	NM_133351	Prss8	protease, serine, 8 (prostasin)	1.95
1416575_at	NM_001161623	Cdc45	cell division cycle 45 homolog (S. cerevisiae)	1.95
1423566_a_at	NM_013559	Hsph1	heat shock 105kDa/110kDa protein 1	1.95
1428593_at	NM_029368		RIKEN cDNA 1700029F09 gene	1.95
1448270_at	NM_019553	Ddx21	DEAD (Asp-Glu-Ala-Asp) box polypeptide 21	1.95
1439341_at	NM_001083628	Greb1l	growth regulation by estrogen in breast cancer-like	1.95
1455035_s_at	NM_024193	Nop56	NOP56 ribonucleoprotein homolog (yeast)	1.96
1456032_x_at	NM_016750		H2A histone family, member Z	1.96
1433696_at	NM_198937	Hn1l	hematological and neurological expressed 1-like	1.96
1438922_x_at	NM_007451	Slc25a5	solute carrier family 25 (mitochondrial carrier, adenine nucleotide translocator), member 5	1.96
1436218_at	NM_001033409	Lgr6	leucine-rich repeat-containing G protein-coupled receptor 6	1.96
1424598_at	NM_001110826	Ddx6	DEAD (Asp-Glu-Ala-Asp) box polypeptide 6	1.96
1437711_x_at	NM_013614	Odc1	ornithine decarboxylase, structural 1	1.96

Probe Set ID	Accession_ID	Gene	Description	Fold
1419820_at	NM_153179	Pkhd1	polycystic kidney and hepatic disease 1	1.96
1429664_at	NM_183294	Cdkl1	cyclin-dependent kinase-like 1 (CDC2-related kinase)	1.96
1416251_at	NM_008567	Mcm6	minichromosome maintenance deficient 6 (MIS5 homolog, S. pombe) (S. cerevisiae)	1.96
1457040_at	NM_144945	Lgi2	leucine-rich repeat LGI family, member 2	1.97
1423635_at	NM_007553	Bmp2	bone morphogenetic protein 2	1.97
1455851_at	NM_007555	Bmp5	bone morphogenetic protein 5	1.97
1417156_at	NM_008471	Krt19	keratin 19	1.97
1452532_x_at	NM_001039185	Ceacam1	carcinoembryonic antigen-related cell adhesion molecule 1	1.97
1438069_a_at	NM_148930	Rbm5	RNA binding motif protein 5	1.97
1416122_at	NM_009829	Ccnd2	cyclin D2	1.97
1423417_at	NM_009211	Smarcc1	SWI/SNF related, matrix associated, actin dependent regulator of chromatin, subfamily c, member 1	1.97
1438015_at	NM_001030307	Dkc1	dyskeratosis congenita 1, dyskerin homolog (human)	1.98
1455099_at	NM_177448	Mogat2	monoacylglycerol O-acyltransferase 2	1.98
1456088_at	NM_009688	Xiap	X-linked inhibitor of apoptosis	1.98
1452917_at	NM_028128	Rfc5	replication factor C (activator 1) 5	1.98
1432538_a_at	NM_027009	Rfc3	replication factor C (activator 1) 3	1.98
1452534_a_at	NM_008252	Hmgb2	high mobility group box 2	1.98
1424207_at	NM_053124	Smarca5	SWI/SNF related, matrix associated, actin dependent regulator of chromatin, subfamily a, member 5	1.98
1447669_s_at	NM_010317	Gng4	guanine nucleotide binding protein (G protein), gamma 4	1.98
1433443_a_at	NM_145942	Hmgcs1	3-hydroxy-3-methylglutaryl-Coenzyme A synthase 1	1.98
1420028_s_at	NM_008563	Mcm3	minichromosome maintenance deficient 3 (S. cerevisiae)	1.98
1437262_x_at	NM_026602	Bcas2	breast carcinoma amplified sequence 2	1.99
1436750_a_at	NM_024188	Oxct1	3-oxoacid CoA transferase 1	1.99
1428224_at	NM_016690	Hnrpdl	heterogeneous nuclear ribonucleoprotein D-like	1.99
1441860_x_at	NM_031156	Ide	insulin degrading enzyme	1.99
1427940_s_at	NM_019660	Mycbp	c-myc binding protein	1.99
1417069_a_at	NM_022023	Gmfb	glia maturation factor, beta	1.99
1456862_at	NM_011382	Six4	sine oculis-related homeobox 4 homolog (Drosophila)	1.99
1423643_at	NM_197982	Ddx39	DEAD (Asp-Glu-Ala-Asp) box polypeptide 39	1.99
1430353_at	NM_175459	Glis3	GLIS family zinc finger 3	1.99
1434539_at	NM_010733	Lrrn3	leucine rich repeat protein 3, neuronal	1.99
1435323_a_at	NM_153546	Mboat1	membrane bound O-acyltransferase domain containing 1	1.99
1447520_at	NM_008489	Lbp	lipopolysaccharide binding protein	2.00
1433543_at	NM_028390	Anln	anillin, actin binding protein	2.00
1417450_a_at	NM_001040435	Tacc3	transforming, acidic coiled-coil containing protein 3	2.00
1456112_at	NM_133780	Tpr	translocated promoter region	2.00
1419074_at	NM_026527	Chac2	ChaC, cation transport regulator homolog 2 (E. coli)	2.00
1428114_at	NM_001171010	Slc14a1	solute carrier family 14 (urea transporter), member 1	2.00
1439208_at	NM_007691	Chek1	checkpoint kinase 1 homolog (S. pombe)	2.00
1428130_at	NM_001172062	Lman1	lectin, mannose-binding, 1	2.00
1437520_a_at	NM_001002929	Nup85	nucleoporin 85	2.01
1459860_x_at	NM_030706	Trim2	tripartite motif-containing 2	2.01
1448793_a_at	NM_011521	Sdc4	syndecan 4	2.01
1427094_at	NM_011133	Pole2	polymerase (DNA directed), epsilon 2 (p59 subunit)	2.01
1417736_at	NM_025695	Smc6	structural maintenance of chromosomes 6	2.01
1420017_at	NM_001168679	Tspan8	tetraspanin 8	2.01
1427707_a_at	NM_009185	Stil	Scl/Tal1 interrupting locus	2.01
1434578_x_at	NM_009391	Ran	RAN, member RAS oncogene family	2.02
1438239_at	NM_010797	Mid1	midline 1	2.02
1422716_a_at	NM_001110239	Acp1	acid phosphatase 1, soluble	2.02
1423714_at	NM_024184	Asf1b	ASF1 anti-silencing function 1 homolog B (S. cerevisiae)	2.02

Probe Set ID	Accession_ID	Gene	Description	Fold
1433445_x_at	NM_145942	Hmgcs1	3-hydroxy-3-methylglutaryl-Coenzyme A synthase 1	2.02
1416641_at	NM_001083188	Lig1	ligase I, DNA, ATP-dependent	2.02
1449893_a_at	NM_008377	Lrig1	leucine-rich repeats and immunoglobulin-like domains 1	2.02
1416076_at	NM_172301	Ccnb1	cyclin B1	2.03
1426612_at	NM_025372	Tipin	timeless interacting protein	2.03
1418472_at	NM_023113	Aspa	aspartoacylase	2.03
1438092_x_at	NM_016750	H2afz	H2A histone family, member Z	2.03
1451319_at	NM_144851	Senp1	SUMO1/sentrin specific peptidase 1	2.03
1453009_at	NM_027468	Cpm	carboxypeptidase M	2.03
1428481_s_at	NM_026560	Cdca8	cell division cycle associated 8	2.03
1451263_a_at	NM_024406	Fabp4	fatty acid binding protein 4, adipocyte	2.03
1448393_at	NM_001193619	Cldn7	claudin 7	2.04
1416258_at	NM_009387	Tk1	thymidine kinase 1	2.04
1418919_at	NM_028232	Sgol1	shugoshin-like 1 (S. pombe)	2.04
1423331_a_at	NM_021495	Pvrl3	poliovirus receptor-related 3	2.04
1429156_at	NM_001109747	Cenpw	centromere protein W	2.04
1426406_at	NM_030241	Setd8	SET domain containing (lysine methyltransferase) 8	2.04
1417568_at	NM_001170866	Ncald	neurocalcin delta	2.04
1434449_at	NM_009700	Aqp4	aquaporin 4	2.05
1418671_at	NM_007602	Capn5	calpain 5	2.05
1451888_a_at	NM_011858	Odz4	odd Oz/ten-m homolog 4 (Drosophila)	2.05
1435521_at	NM_054043	Msi2	Musashi homolog 2 (Drosophila)	2.05
1418405_at	NM_019447	Hgfac	hepatocyte growth factor activator	2.05
1453107_s_at	NM_008021		forkhead box M1 /// phosphatidylethanolamine binding protein 1	2.05
1429274_at	NM_027990	Lypd6b	LY6/PLAUR domain containing 6B	2.05
1436453_at	BB144871	BB144871	expressed sequence BB144871	2.05
1428304_at	NM_028039	Esco2	establishment of cohesion 1 homolog 2 (S. cerevisiae)	2.06
1418057_at	NM_001145886	Tiam1	T-cell lymphoma invasion and metastasis 1	2.07
1436450_at	NM_001024931	D11Bwg0517e	DNA segment, Chr 11, Brigham & Women's Genetics 0517 expressed	2.07
1426865_a_at	NM_001081445	Ncam1	neural cell adhesion molecule 1	2.07
1434606_at	NM_010153	Erbb3	v-erb-b2 erythroblastic leukemia viral oncogene homolog 3 (avian)	2.07
1427398_at	NM_080457	Muc4	mucin 4	2.07
1415878_at	NM_009103	Rrm1	ribonucleotide reductase M1	2.07
1430596_s_at	NM_028572	Vgll3	vestigial like 3 (Drosophila)	2.07
1434079_s_at	NM_008564	Mcm2	minichromosome maintenance deficient 2 mitotin (S. cerevisiae)	2.08
1453314_x_at	NM_025642		RIKEN cDNA 2610039C10 gene	2.08
1426473_at	NM_134081	Dnajc9	DnaJ (Hsp40) homolog, subfamily C, member 9	2.08
1438434_at	NM_181416	Arhgap11a	Rho GTPase activating protein 11A	2.08
1424144_at	NM_026014	Cdt1	chromatin licensing and DNA replication factor 1	2.08
1438168_x_at	NM_197982	Ddx39	DEAD (Asp-Glu-Ala-Asp) box polypeptide 39	2.08
1427276_at	NM_133786	Smc4	structural maintenance of chromosomes 4	2.08
1426805_at	NM_001174078	Smarca4	SWI/SNF related, matrix associated, actin dependent regulator of chromatin, subfamily a, member 4	2.08
1435033_at	NM_183019	Arhgef4	Rho guanine nucleotide exchange factor (GEF) 4	2.09
1460681_at	NM_001039185	Ceacam1	carcinoembryonic antigen-related cell adhesion molecule 1	2.09
1419231_s_at	NM_010661	Krt12	keratin 12	2.09
1435114_at	NM_172598	Wdhd1	WD repeat and HMG-box DNA binding protein 1	2.09
1450634_at	NM_007508	Atp6v1a	ATPase, H+ transporting, lysosomal V1 subunit A	2.09
1444952_a_at	NM_001145804	Nucks1	nuclear casein kinase and cyclin-dependent kinase substrate 1	2.09
1451920_a_at	NM_011258	Rfc1	replication factor C (activator 1) 1	2.09
1452703_at	NM_001171000	Ahcyl2	S-adenosylhomocysteine hydrolase-like 2	2.09
1419571_at	NM_022317	Slc28a3	solute carrier family 28 (sodium-coupled nucleoside transporter), member 3	2.09

Probe Set ID	Accession_ID	Gene	Description	Fold
1439764_s_at	NM_183029	Igf2bp2	insulin-like growth factor 2 mRNA binding protein 2	2.10
1424351_at	NM_026323	Wfdc2	WAP four-disulfide core domain 2	2.10
1430111_a_at	NM_001024468	Bcat1	branched chain aminotransferase 1, cytosolic	2.10
1424450_at	NM_001110337	Gprc5c	G protein-coupled receptor, family C, group 5, member C	2.10
1427939_s_at	NM_019660	Mycbp	c-myc binding protein	2.11
1437313_x_at	NM_008252	Hmgb2	high mobility group box 2	2.11
1416376_at	NM_133706	Tmem97	transmembrane protein 97	2.11
1450618_a_at	NM_001164787		small proline-rich protein 2A1 /// small proline-rich protein 2A2	2.11
1431087_at	NM_026282	Spc24	SPC24, NDC80 kinetochore complex component, homolog (S. cerevisiae)	2.11
1417419_at	NM_007631	Ccnd1	cyclin D1	2.11
1454963_at	NM_178668	Pde12	phosphodiesterase 12	2.11
1417938_at	NM_009013	Rad51ap1	RAD51 associated protein 1	2.11
1419172_at	NM_010049	Dhfr	dihydrofolate reductase	2.12
1417932_at	NM_008360	Il18	interleukin 18	2.12
1428713_s_at	NM_178856	Gins2	GINS complex subunit 2 (Psf2 homolog)	2.12
1421731_a_at	NM_007999	Fen1	flap structure specific endonuclease 1	2.12
1417458_s_at	NM_025415	Cks2	CDC28 protein kinase regulatory subunit 2	2.12
1439122_at	NM_001110826	Ddx6	DEAD (Asp-Glu-Ala-Asp) box polypeptide 6	2.13
1433444_at	NM_145942	Hmgcs1	3-hydroxy-3-methylglutaryl-Coenzyme A synthase 1	2.13
1455956_x_at	NM_009829	Ccnd2	cyclin D2	2.13
1438977_x_at	NM_009391	Ran	RAN, member RAS oncogene family	2.13
1439442_x_at	NM_198246	Yars2	tyrosyl-tRNA synthetase 2 (mitochondrial)	2.14
1425298_a_at	NM_008670	Naip1	NLR family, apoptosis inhibitory protein 1	2.14
1417023_a_at	NM_024406	Fabp4	fatty acid binding protein 4, adipocyte	2.14
1417623_at	NM_009194	Slc12a2	solute carrier family 12, member 2	2.14
1417506_at	NM_020567	Gmnn	geminin	2.14
1452608_at	NM_019660	Mycbp	c-myc binding protein	2.14
1424321_at	NM_145480	Rfc4	replication factor C (activator 1) 4	2.14
1436847_s_at	NM_026560	Cdca8	cell division cycle associated 8	2.14
1435945_a_at	NM_001163510	Kcnn4	potassium intermediate/small conductance calcium-activated channel, subfamily N, member 4	2.15
1417457_at	NM_025415	Cks2	CDC28 protein kinase regulatory subunit 2	2.15
1454254_s_at	NM_029639		RIKEN cDNA 1600029D21 gene	2.15
1450079_at	NM_013724	Nrk	Nik related kinase	2.15
1438322_x_at	NM_010191	Fdft1	farnesyl diphosphate farnesyl transferase 1	2.16
1434745_at	NM_009829	Ccnd2	cyclin D2	2.16
1438021_at	NM_001127382	Rbm47	RNA binding motif protein 47	2.16
1424278_a_at	NM_001012273	Birc5	baculoviral IAP repeat-containing 5	2.16
1438040_a_at	NM_011631	Hsp90b1	heat shock protein 90, beta (Grp94), member 1	2.16
1451786_at	NM_001145874	Muc20	mucin 20	2.16
1428936_at	NM_026482	Atp2b1	ATPase, Ca++ transporting, plasma membrane 1	2.17
1419513_a_at	NM_001177625	Ect2	ect2 oncogene	2.17
1426864_a_at	NM_001081445	Ncam1	neural cell adhesion molecule 1	2.17
1448227_at	NM_010346	Grb7	growth factor receptor bound protein 7	2.17
1423142_a_at	NM_027000	Gtpbp4	GTP binding protein 4	2.17
1434767_at	NM_172578	C79407	expressed sequence C79407	2.17
1452497_a_at	NM_010901	Nfatc3	nuclear factor of activated T-cells, cytoplasmic, calcineurin-dependent 3	2.17
1423877_at	NM_028083	Chaf1b	chromatin assembly factor 1, subunit B (p60)	2.17
1418672_at	NM_013778	Akr1c13	aldo-keto reductase family 1, member C13	2.17
1417353_x_at	NM_021336	Snrpa1	small nuclear ribonucleoprotein polypeptide A'	2.17
1436237_at	NM_001033149	Ttc9	tetratricopeptide repeat domain 9	2.18
1423367_at	NM_009527	Wnt7a	wingless-related MMTV integration site 7A	2.18

Probe Set ID	Accession_ID	Gene	Description	Fold
1423428_at	NM_013846	Ror2	receptor tyrosine kinase-like orphan receptor 2	2.18
1442143_at	NM_178773	Ano4	anoctamin 4	2.18
1423954_at	NM_009778	C3	complement component 3	2.18
1416421_a_at	NM_001110145	Ssb	Sjogren syndrome antigen B	2.19
1416022_at	NM_010634	Fabp5	fatty acid binding protein 5, epidermal	2.19
1416715_at	NM_001160012	Gjb3	gap junction protein, beta 3	2.19
1450986_at	NM_018868	Nop58	NOP58 ribonucleoprotein homolog (yeast)	2.19
1427929_a_at	NM_172134	Pdxk	pyridoxal (pyridoxine, vitamin B6) kinase	2.19
1438271_at	NM_001145952	Lpp	LIM domain containing preferred translocation partner in lipoma	2.20
1440147_at	NM_144945	Lgi2	leucine-rich repeat LGI family, member 2	2.20
1434628_a_at	NM_027897	Rhpn2	rhophilin, Rho GTPase binding protein 2	2.20
1418431_at	NM_008448	Kif5b	kinesin family member 5B	2.20
1455836_at	NM_011112	Papola	poly (A) polymerase alpha	2.20
1449740_s_at	NM_007883	Dsg2	desmoglein 2	2.20
1448663_s_at	NM_138656	Mvd	mevalonate (diphospho) decarboxylase	2.21
1435630_s_at	NM_009338	Acat2	acetyl-Coenzyme A acetyltransferase 2	2.21
1436174_at	NM_027435	Atad2	ATPase family, AAA domain containing 2	2.21
1437251_at	NM_001110162	Cdca2	cell division cycle associated 2	2.22
1455814_x_at	NM_197982	Ddx39	DEAD (Asp-Glu-Ala-Asp) box polypeptide 39	2.22
1453593_at	NM_028572	Vgll3	vestigial like 3 (Drosophila)	2.22
1438480_a_at	NM_144543	Thyn1	thymocyte nuclear protein 1	2.23
1438306_at	NM_027934	Rnf180	ring finger protein 180	2.23
1453231_at	NM_183294	Cdkl1	cyclin-dependent kinase-like 1 (CDC2-related kinase)	2.23
1418924_at	NM_025886	Rassf7	Ras association (RalGDS/AF-6) domain family (N-terminal) member 7	2.23
1452087_at	NM_029495	Epsti1	epithelial stromal interaction 1 (breast)	2.24
1429072_at	NM_026763	Col6a4	collagen, type VI, alpha 4	2.24
1424254_at	NM_001112715	Ifitm1	interferon induced transmembrane protein 1	2.24
1419153_at	NM_026515		RIKEN cDNA 2810417H13 gene	2.25
1427261_at	NM_170779	Wwc1	WW, C2 and coiled-coil domain containing 1	2.25
1451210_at	NM_015817	Ppap2c	phosphatidic acid phosphatase type 2C	2.25
1434437_x_at	NM_009104	Rrm2	ribonucleotide reductase M2	2.26
1433408_a_at	NM_027290	Mcm10	minichromosome maintenance deficient 10 (S. cerevisiae)	2.26
1438161_s_at	NM_145480	Rfc4	replication factor C (activator 1) 4	2.26
1451246_s_at	NM_011496	Aurkb	aurora kinase B	2.26
1452881_at	NM_178856	Gins2	GINS complex subunit 2 (Psf2 homolog)	2.26
1436839_at	NM_001162903		RIKEN cDNA 2010109K11 gene	2.27
1435306_a_at	NM_010615	Kif11	kinesin family member 11	2.27
1424046_at	NM_001113179	Bub1	budding uninhibited by benzimidazoles 1 homolog (S. cerevisiae)	2.28
1422680_at	NM_009431	Ctr9	Ctr9, Paf1/RNA polymerase II complex component, homolog (S. cerevisiae)	2.28
1429660_s_at	NM_008017	Smc2	structural maintenance of chromosomes 2	2.28
1456379_x_at	NM_152915	Dner	delta/notch-like EGF-related receptor	2.28
1453264_at	NM_028584	Marveld3	MARVEL (membrane-associating) domain containing 3	2.29
1426652_at	NM_008563	Mcm3	minichromosome maintenance deficient 3 (S. cerevisiae)	2.29
1436519_a_at	NM_001167767		RIKEN cDNA 1110057K04 gene	2.29
1443536_at	NM_011990	Slc7a11	solute carrier family 7 (cationic amino acid transporter, y+ system), member 11	2.30
1431805_a_at	NM_027897	Rhpn2	rhophilin, Rho GTPase binding protein 2	2.31
1452226_at	NM_173867	Rcc2	regulator of chromosome condensation 2	2.31
1448650_a_at	NM_011132	Pole	polymerase (DNA directed), epsilon	2.31
1441788_s_at	NM_001030307	Dkc1	dyskeratosis congenita 1, dyskerin homolog (human)	2.32
1457157_at	NM_001177732	Plch1	phospholipase C, eta 1	2.32

Probe Set ID	Accession_ID	Gene	Description	Fold
1416492_at	NM_007633	Ccne1	cyclin E1	2.32
1438360_x_at	NM_007451	Slc25a5	solute carrier family 25 (mitochondrial carrier, adenine nucleotide translocator), member 5	2.33
1417828_at	NM_001109045	Aqp8	aquaporin 8	2.33
1424296_at	NM_010295	Gclc	glutamate-cysteine ligase, catalytic subunit	2.33
1423933_a_at	NM_029639		RIKEN cDNA 1600029D21 gene	2.33
1430127_a_at	NM_009829	Ccnd2	cyclin D2	2.34
1448818_at	NM_009524	Wnt5a	wingless-related MMTV integration site 5A	2.34
1452626_a_at	NM_026928		RIKEN cDNA 1810014F10 gene	2.35
1426533_at	NM_024193	Nop56	NOP56 ribonucleoprotein homolog (yeast)	2.35
1441917_s_at	NM_001168256	Tmem40	transmembrane protein 40	2.36
1424118_a_at	NM_025565	Spc25	SPC25, NDC80 kinetochore complex component, homolog (S. cerevisiae)	2.37
1449705_x_at	NM_008563	Mcm3	minichromosome maintenance deficient 3 (S. cerevisiae)	2.37
1423674_at	NM_146144	Usp1	ubiquitin specific peptidase 1	2.38
1450051_at	NM_009530	Atrx	alpha thalassemia/mental retardation syndrome X-linked homolog (human)	2.38
1433804_at	NM_146145	Jak1	Janus kinase 1	2.38
1416299_at	NM_011369	Shcbp1	Shc SH2-domain binding protein 1	2.38
1448906_at	NM_007663	Cdh16	cadherin 16	2.38
1416368_at	NM_010357	Gsta4	glutathione S-transferase, alpha 4	2.39
1418129_at	NM_053272	Dhcr24	24-dehydrocholesterol reductase	2.39
1441926_x_at	NM_146260	Tmie	transmembrane inner ear	2.40
1454728_s_at	NM_001038999	Atp8a1	ATPase, aminophospholipid transporter (APLT), class I, type 8A, member 1	2.40
1418724_at	NM_007686	Cfi	complement component factor i	2.41
1452227_at	NM_172710	Sel1l3	sel-1 suppressor of lin-12-like 3 (C. elegans)	2.41
1419148_at	NM_009635	Avil	advillin	2.41
1448794_s_at	NM_009584	Dnajc2	DnaJ (Hsp40) homolog, subfamily C, member 2	2.41
1416034_at	NM_009846	Cd24a	CD24a antigen	2.42
1422430_at	NM_001163359	Fignl1	fidgetin-like 1	2.42
1452679_at	NM_023716	Tubb2b	tubulin, beta 2B	2.43
1422842_at	NM_011917	Xrn2	5'-3' exoribonuclease 2	2.43
1415802_at	NM_009196	Slc16a1	solute carrier family 16 (monocarboxylic acid transporters), member 1	2.43
1448263_a_at	NM_023149	Cndp2	CNDP dipeptidase 2 (metallopeptidase M20 family)	2.44
1434913_at	NM_173731	Hmgcll1	3-hydroxymethyl-3-methylglutaryl-Coenzyme A lyase-like 1	2.45
1449171_at	NM_001110265	Ttk	Ttk protein kinase	2.45
1435641_at	NM_173870	Mgat4a	mannoside acetylglucosaminyltransferase 4, isoenzyme A	2.46
1448627_s_at	NM_023209	Pbk	PDZ binding kinase	2.47
1460406_at	NM_001033210	Pls1	plastin 1 (I-isoform)	2.48
1460177_at	NM_023149	Cndp2	CNDP dipeptidase 2 (metallopeptidase M20 family)	2.49
1450455_s_at	NM_013777		aldo-keto reductase family 1, member C12 /// aldo-keto reductase family 1, member C13	2.49
1429844_at	NM_027158		RIKEN cDNA 2310043J07 gene	2.49
1418281_at	NM_011234	Rad51	RAD51 homolog (S. cerevisiae)	2.50
1418930_at	NM_021274	Cxcl10	chemokine (C-X-C motif) ligand 10	2.50
1429295_s_at	NM_027182	Trip13	thyroid hormone receptor interactor 13	2.50
1427931_s_at	NM_172134	Pdxk	pyridoxal (pyridoxine, vitamin B6) kinase	2.50
1425163_at	NM_145489	AI661453	expressed sequence AI661453	2.50
1417823_at	NM_001161712	Gcat	glycine C-acetyltransferase (2-amino-3-ketobutyrate-coenzyme A ligase)	2.51
1439357_at	NM_001034029	Il17re	interleukin 17 receptor E	2.51
1451065_a_at	NM_197982	Ddx39	DEAD (Asp-Glu-Ala-Asp) box polypeptide 39	2.51
1427229_at	NM_008255	Hmgcr	3-hydroxy-3-methylglutaryl-Coenzyme A reductase	2.51

Probe Set ID	Accession_ID	Gene	Description	Fold
1417896_at	NM_013769	Tjp3	tight junction protein 3	2.51
1436808_x_at	NM_008566	Mcm5	minichromosome maintenance deficient 5, cell division cycle 46 (S. cerevisiae)	2.52
1436021_at	NM_001114662	Mfsd4	major facilitator superfamily domain containing 4	2.52
1419717_at	NM_011348	Sema3e	sema domain, immunoglobulin domain (Ig), short basic domain, secreted, (semaphorin) 3E	2.53
1422460_at	NM_019499	Mad2l1	MAD2 mitotic arrest deficient-like 1 (yeast)	2.54
1421821_at	NM_010700	Ldlr	low density lipoprotein receptor	2.54
1416661_at	NM_010123	Eif3a	eukaryotic translation initiation factor 3, subunit A	2.54
1424292_at	NM_001172092	Depdc1a	DEP domain containing 1a	2.54
1418024_at	NM_053089	Naa15	N(alpha)-acetyltransferase 15, NatA auxiliary subunit	2.55
1422123_s_at	NM_001039185		carcinoembryonic antigen-related cell adhesion molecule 1 /// carcinoembryonic antigen-related cell adhesion molecule 2	2.55
1416596_at	NM_023557	Slc44a4	solute carrier family 44, member 4	2.56
1436704_x_at	NM_138745	Mthfd1	methylenetetrahydrofolate dehydrogenase (NADP+ dependent), methenyltetrahydrofolate cyclohydrolase, formyltetrahydrofolate synthase	2.57
1452912_at	NM_183089	Dscc1	defective in sister chromatid cohesion 1 homolog (S. cerevisiae)	2.57
1416802_a_at	NM_026410	Cdca5	cell division cycle associated 5	2.57
1450988_at	NM_010195	Lgr5	leucine rich repeat containing G protein coupled receptor 5	2.57
1448777_at	NM_008564	Mcm2	minichromosome maintenance deficient 2 mitotin (S. cerevisiae)	2.57
1436335_at	NM_001113360	Plch2	phospholipase C, eta 2	2.58
1418264_at	NM_021790	Cenpk	centromere protein K	2.60
1415945_at	NM_008566	Mcm5	minichromosome maintenance deficient 5, cell division cycle 46 (S. cerevisiae)	2.60
1418076_at	NM_011176	St14	suppression of tumorigenicity 14 (colon carcinoma)	2.60
1420352_at	NM_133731	Prss22	protease, serine, 22	2.61
1449708_s_at	NM_007691	Chek1	checkpoint kinase 1 homolog (S. pombe)	2.62
1450780_s_at	NM_010441	Hmga2	high mobility group AT-hook 2	2.63
1460682_s_at	NM_001039185		carcinoembryonic antigen-related cell adhesion molecule 1 /// carcinoembryonic antigen-related cell adhesion molecule 2	2.63
1422533_at	NM_020010	Cyp51	cytochrome P450, family 51	2.63
1448235_s_at	NM_001111282		high-mobility group (nonhistone chromosomal) protein 1-like 1	2.64
1434695_at	NM_029766	Dtl	denticleless homolog (Drosophila)	2.64
1439793_at	NM_016975	Gja3	gap junction protein, alpha 3	2.64
1428859_at	NM_153783	Paox	polyamine oxidase (exo-N4-amino)	2.64
1417588_at	NM_015736	Galnt3	UDP-N-acetyl-alpha-D-galactosamine:polypeptide N-acetylgalactosaminyltransferase 3	2.65
1448743_at	NM_138744	Ssx2ip	synovial sarcoma, X breakpoint 2 interacting protein	2.68
1418457_at	NM_019568	Cxcl14	chemokine (C-X-C motif) ligand 14	2.68
1448130_at	NM_010191	Fdft1	farnesyl diphosphate farnesyl transferase 1	2.68
1439476_at	NM_007883	Dsg2	desmoglein 2	2.68
1450494_x_at	NM_001039185	Ceacam1	carcinoembryonic antigen-related cell adhesion molecule 1	2.68
1438009_at	NM_001177544		predicted gene 11276 /// histone cluster 1, H2ao	2.69
1426301_at	NM_009655	Alcam	activated leukocyte cell adhesion molecule	2.71
1425452_s_at	NM_029007	Fam84a	family with sequence similarity 84, member A	2.71
1450035_a_at	NM_018785	Prpf40a	PRP40 pre-mRNA processing factor 40 homolog A (yeast)	2.71
1422760_at	NM_011061	Padi4	peptidyl arginine deiminase, type IV	2.72
1416558_at	NM_010790	Melk	maternal embryonic leucine zipper kinase	2.73
1417812_a_at	NM_008484	Lamb3	laminin, beta 3	2.74
1439568_at	NM_015764	Greb1	gene regulated by estrogen in breast cancer protein	2.74
1448899_s_at	NM_009013	Rad51ap1	RAD51 associated protein 1	2.74
1418601_at	NM_011921	Aldh1a7	aldehyde dehydrogenase family 1, subfamily A7	2.75
1417738_at	NM_016899	Rab25	RAB25, member RAS oncogene family	2.76
1435494_s_at	NM_023842	Dsp	desmoplakin	2.76

Probe Set ID	Accession_ID	Gene	Description	Fold
1442058_s_at	NM_008949	Psmc3ip	proteasome (prosome, macropain) 26S subunit, ATPase 3, interacting protein	2.76
1420579_s_at	NM_021050	Cftr	cystic fibrosis transmembrane conductance regulator homolog	2.77
1448229_s_at	NM_009829	Ccnd2	cyclin D2	2.77
1449484_at	NM_011491	Stc2	stanniocalcin 2	2.77
1419593_at	NM_015764	Greb1	gene regulated by estrogen in breast cancer protein	2.77
1441963_at	NM_197945	Prosapip1	ProSAPiP1 protein	2.77
1419230_at	NM_010661	Krt12	keratin 12	2.77
1425029_a_at	NM_001083341	Mboat2	membrane bound O-acyltransferase domain containing 2	2.78
1440888_at	NM_001081147	Oxtr	oxytocin receptor	2.79
1432339_at	AK017023		RIKEN cDNA 4933432I03 gene	2.80
1436708_x_at	NM_008565	Mcm4	minichromosome maintenance deficient 4 homolog (S. cerevisiae)	2.81
1426573_at	NM_145494		NAD-dependent malic enzyme, mitochondrial-like	2.82
1435493_at	NM_023842	Dsp	desmoplakin	2.83
1451064_a_at	NM_177420	Psat1	phosphoserine aminotransferase 1	2.85
1454788_at	NM_177305	Arl4c	ADP-ribosylation factor-like 4C	2.87
1422851_at	NM_010441	Hmga2	high mobility group AT-hook 2	2.88
1452257_at	NM_001122683	Bdh1	3-hydroxybutyrate dehydrogenase, type 1	2.88
1438852_x_at	NM_008567	Mcm6	minichromosome maintenance deficient 6 (MIS5 homolog, S. pombe) (S. cerevisiae)	2.88
1437502_x_at	NM_009846	Cd24a	CD24a antigen	2.90
1437497_a_at	NM_010480	Hsp90aa1	heat shock protein 90, alpha (cytosolic), class A member 1	2.90
1423418_at	NM_134469	Fdps	farnesyl diphosphate synthetase	2.91
1429364_at	NM_029482		RIKEN cDNA 4930579G24 gene	2.92
1426572_at	NM_145494		NAD-dependent malic enzyme, mitochondrial-like /// malic enzyme 2, NAD(+)-dependent, mitochondrial	2.93
1429413_at	NM_027468	Cpm	carboxypeptidase M	2.93
1448226_at	NM_009104	Rrm2	ribonucleotide reductase M2	2.94
1424629_at	NM_009764	Brca1	breast cancer 1	2.95
1418937_at	NM_010050	Dio2	deiodinase, iodothyronine, type II	2.97
1419152_at	NM_026515		RIKEN cDNA 2810417H13 gene	2.97
1436186_at	NM_001013368	E2f8	E2F transcription factor 8	2.97
1423952_a_at	NM_033073	Krt7	keratin 7	2.98
1455516_at	NM_153409	Csrnp3	cysteine-serine-rich nuclear protein 3	3.00
1426911_at	NM_013505	Dsc2	desmocollin 2	3.00
1441971_at	AW543723			3.00
1430143_at	AK015205		RIKEN cDNA 4930426D05 gene	3.00
1422535_at	NM_001037134	Ccne2	cyclin E2	3.03
1418252_at	NM_008812	Padi2	peptidyl arginine deiminase, type II	3.04
1438799_at	NR_015388	Dlx6os1	Dlx6 opposite strand transcript 1	3.05
1454607_s_at	NM_177420	Psat1	phosphoserine aminotransferase 1	3.07
1437110_at	NM_026054		RIKEN cDNA 2810474O19 gene	3.08
1457044_at	NM_001163136	Macc1	metastasis associated in colon cancer 1	3.09
1417019_a_at	NM_001025779	Cdc6	cell division cycle 6 homolog (S. cerevisiae)	3.10
1451527_at	NM_029620	Pcolce2	procollagen C-endopeptidase enhancer 2	3.12
1450871_a_at	NM_001024468	Bcat1	branched chain aminotransferase 1, cytosolic	3.14
1415993_at	NM_009270	Sqle	squalene epoxidase	3.15
1436115_at	NM_001033248	Gm266	predicted gene 266	3.18
1436512_at	NM_177305	Arl4c	ADP-ribosylation factor-like 4C	3.29
1419072_at	NM_026672	Gstm7	glutathione S-transferase, mu 7	3.32
1416123_at	NM_009829	Ccnd2	cyclin D2	3.34
1417789_at	NM_011330	Ccl11	chemokine (C-C motif) ligand 11	3.34
1434278_at	NM_001164190	Mtm1	X-linked myotubular myopathy gene 1	3.38

Probe Set ID	Accession_ID	Gene	Description	Fold
1448752_at	NM_009801	Car2	carbonic anhydrase 2	3.39
1427334_s_at	NM_026054		RIKEN cDNA 2810474O19 gene	3.39
1418509_at	NM_007621	Cbr2	carbonyl reductase 2	3.40
1449199_at	NM_013605	Muc1	mucin 1, transmembrane	3.44
1452661_at	NM_011638	Tfrc	transferrin receptor	3.46
1424649_a_at	NM_001168679	Tspan8	tetraspanin 8	3.49
1460463_at	XM_001476279	H60c	histocompatibility 60c	3.51
1437760_at	NM_172693	Galnt12	UDP-N-acetyl-alpha-D-galactosamine:polypeptide N-acetylgalactosaminyltransferase 12	3.52
1435486_at	NM_001195046	Pak3	p21 protein (Cdc42/Rac)-activated kinase 3	3.52
1415810_at	NM_001111078	Uhrf1	ubiquitin-like, containing PHD and RING finger domains, 1	3.53
1416271_at	NM_022032	Perp	PERP, TP53 apoptosis effector	3.61
1448881_at	NM_017370	Hp	haptoglobin	3.63
1455186_a_at	NM_029821		RIKEN cDNA 1190003J15 gene	3.63
1433579_at	NM_178715	Tmem30b	transmembrane protein 30B	3.63
1418938_at	NM_010050	Dio2	deiodinase, iodothyronine, type II	3.69
1425753_a_at	NM_001040691	Ung	uracil DNA glycosylase	3.69
1426858_at	NM_008381	Inhbb	inhibin beta-B	3.70
1450646_at	NM_020010	Cyp51	cytochrome P450, family 51	3.80
1425538_x_at	NM_001039185	Ceacam1	carcinoembryonic antigen-related cell adhesion molecule 1	3.87
1431786_s_at	NM_029821		RIKEN cDNA 1190003J15 gene	3.90
1417541_at	NM_008234	Hells	helicase, lymphoid specific	3.91
1452716_at	NM_027464		RIKEN cDNA 5730469M10 gene	3.93
1420378_at	NM_009160	Sftpd	surfactant associated protein D	3.94
1427630_x_at	NM_001039185	Ceacam1	carcinoembryonic antigen-related cell adhesion molecule 1	3.96
1424502_at	NM_146050	Oit1	oncoprotein induced transcript 1	3.97
1423597_at	NM_001038999	Atp8a1	ATPase, aminophospholipid transporter (APLT), class I, type 8A, member 1	4.01
1449254_at	NM_009263	Spp1	secreted phosphoprotein 1	4.08
1450194_a_at	NM_010848	Myb	myeloblastosis oncogene	4.16
1418449_at	NM_133664	Lad1	ladinin	4.17
1434877_at	NM_008730	Nptx1	neuronal pentraxin 1	4.17
1418649_at	NM_028133	Egln3	EGL nine homolog 3 (C. elegans)	4.22
1425675_s_at	NM_001039185	Ceacam1	carcinoembryonic antigen-related cell adhesion molecule 1	4.24
1418979_at	NM_134072	Akr1c14	aldo-keto reductase family 1, member C14	4.27
1423078_a_at	NM_025436	Sc4mol	sterol-C4-methyl oxidase-like	4.31
1438512_at	NM_001193274	BC048679	cDNA sequence BC048679	4.32
1451895_a_at	NM_053272	Dhcr24	24-dehydrocholesterol reductase	4.36
1426302_at	NM_145403	Tmprss4	transmembrane protease, serine 4	4.45
1419332_at	NM_019397	Egfl6	EGF-like-domain, multiple 6	4.47
1416916_at	NM_001163131	Elf3	E74-like factor 3	4.50
1417314_at	NM_001142706		complement component 2 (within H-2S) /// complement factor B	4.51
1448566_at	NM_016917	Slc40a1	solute carrier family 40 (iron-regulated transporter), member 1	4.53
1426566_s_at	NM_001034029	Il17re	interleukin 17 receptor E	4.54
1451122_at	NM_145360	Idi1	isopentenyl-diphosphate delta isomerase	4.82
1447774_x_at	NM_027464		RIKEN cDNA 5730469M10 gene	4.93
1421317_x_at	NM_010848	Myb	myeloblastosis oncogene	4.98
1434280_at	BG976607			5.09
1448265_x_at	NM_007962	Mpzl2	myelin protein zero-like 2	5.23
1417895_a_at	NM_025452	Tmem54	transmembrane protein 54	5.35
1423804_a_at	NM_145360	Idi1	isopentenyl-diphosphate delta isomerase	5.57
1455531_at	NM_001114662	Mfsd4	major facilitator superfamily domain containing 4	5.67
1417061_at	NM_016917	Slc40a1	solute carrier family 40 (iron-regulated transporter), member 1	5.81
1426872_at	NM_001122603	Fcgbp	Fc fragment of IgG binding protein	5.93

Probe Set ID	Accession_ID	Gene	Description	Fold
1416236_a_at	NM_007962	Mpzl2	myelin protein zero-like 2	6.00
1455454_at	NM_001013785	Akr1c19	aldo-keto reductase family 1, member C19	6.32
1417155_at	NM_008709	Mycn	v-myc myelocytomatosis viral related oncogene, neuroblastoma derived (avian)	7.73
1424357_at	NM_144936	Tmem45b	transmembrane protein 45b	8.01
1423323_at	NM_020047	Tacstd2	tumor-associated calcium signal transducer 2	8.06
1449833_at	NM_011472	Sprr2f	small proline-rich protein 2F	8.32
1419323_at	NM_011059	Padi1	peptidyl arginine deiminase, type I	8.36
1434719_at	NM_175628	A2m	alpha-2-macroglobulin	8.53
1438555_x_at	NM_080457	Muc4	mucin 4	9.16
1424733_at	NM_001008497	P2ry14	purinergic receptor P2Y, G-protein coupled, 14	9.23
1420401_a_at	NM_019511	Ramp3	receptor (calcitonin) activity modifying protein 3	9.54
1427042_at	NM_178920	Mal2	mal, T-cell differentiation protein 2	9.55
1422604_at	NM_009474	Uox	urate oxidase	12.22
1450009_at	NM_008522	Ltf	lactotransferrin	12.75
1453503_at	NM_030061	Spink12	serine peptidase inhibitor, Kazal type 11	13.04
1423271_at	NM_008125	Gjb2	gap junction protein, beta 2	17.09
1455996_x_at	NM_009475	Prap1	proline-rich acidic protein 1	23.07
1419167_at	NM_009475	Prap1	proline-rich acidic protein 1	30.47
1416306_at	NM_017474	Clca3	chloride channel calcium activated 3	36.21
1427747_a_at	NM_008491	Lcn2	lipocalin 2	62.12
