# Supplementary figures and images for: ARID1A Is Essential for Endometrial Function during Early Pregnancy
Source: PLoS Genet. 2015 Sep 17;11(9):e1005537. doi: 10.1371/journal.pgen.1005537 (PMC4574948; doi:10.1371/journal.pgen.1005537)

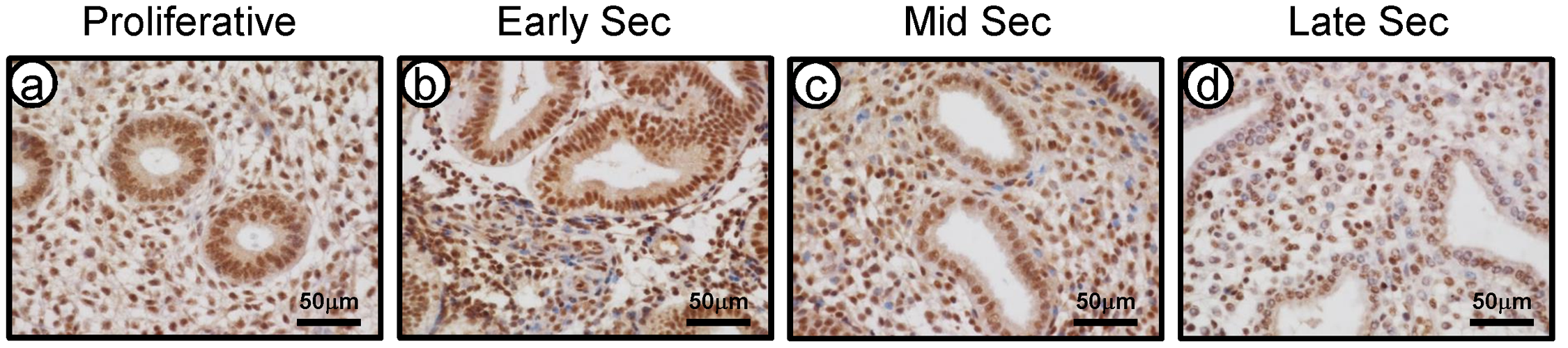

Supplement: S1 Fig — (TIF) [file pgen.1005537.s004.tif]

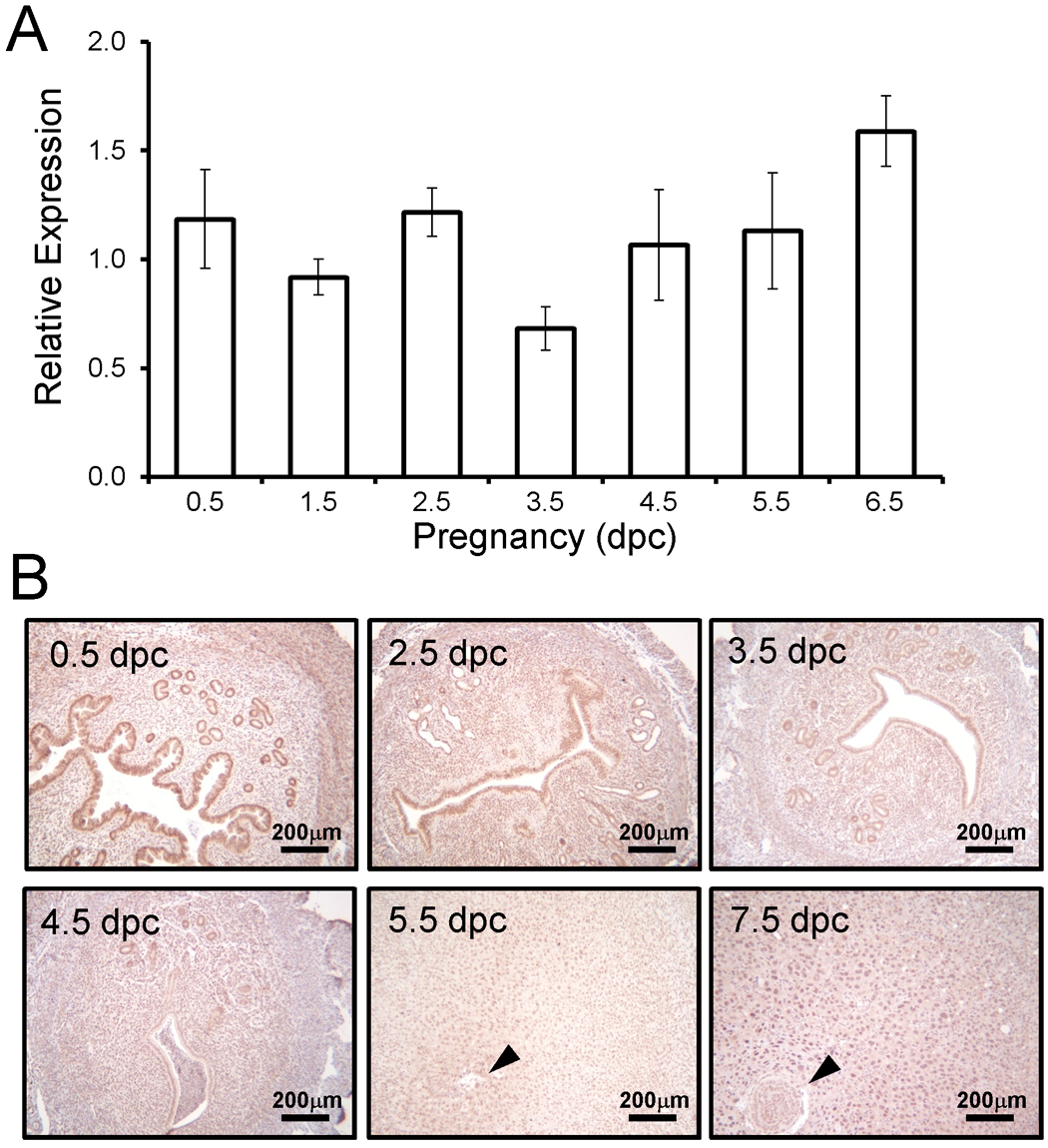

Supplement: S2 Fig — (A) The expression level of Arid1a was measured in uteri of pseudopregnancy. Total RNA used for the RT-PCR assays was prepared from pseudopregnant uteri. (B) The localization pattern of ARID1A by immunohistochemical analysis during early pregnancy. (TIF) [file pgen.1005537.s005.tif]

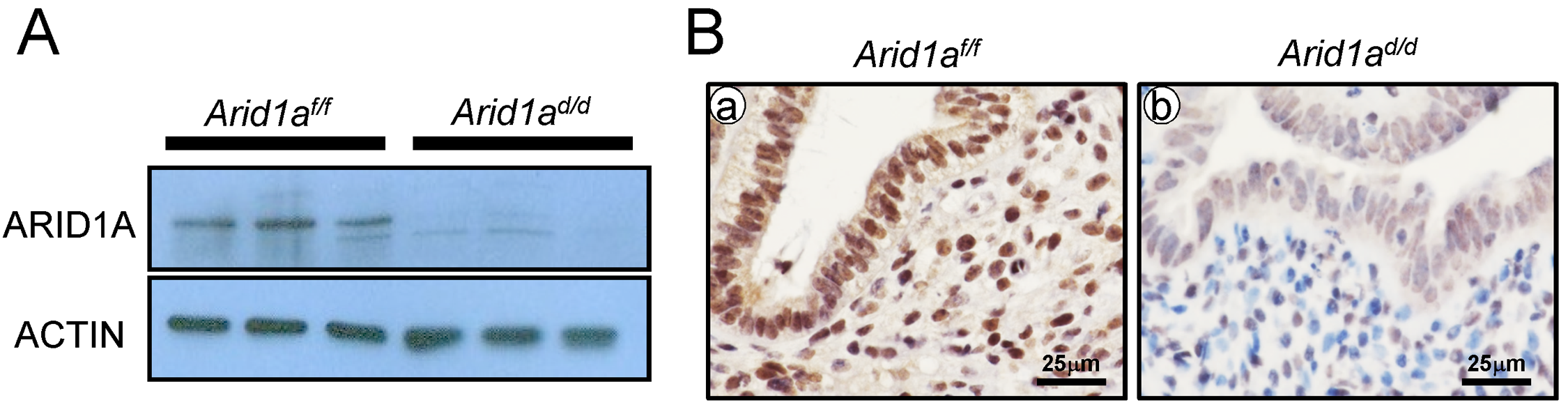

Supplement: S3 Fig — (A) Western blot analysis of ARID1A and Actin in whole uterine of control (Arid1a f/f) and Arid1a d/d mice at 6 weeks of age. Equal amounts of protein were subjected to SDS-PAGE and Western blot analysis. (B) Immunohistochemical analysis of ARID1A in control and Arid1a d/d mice (a and b). (TIF) [file pgen.1005537.s006.tif]

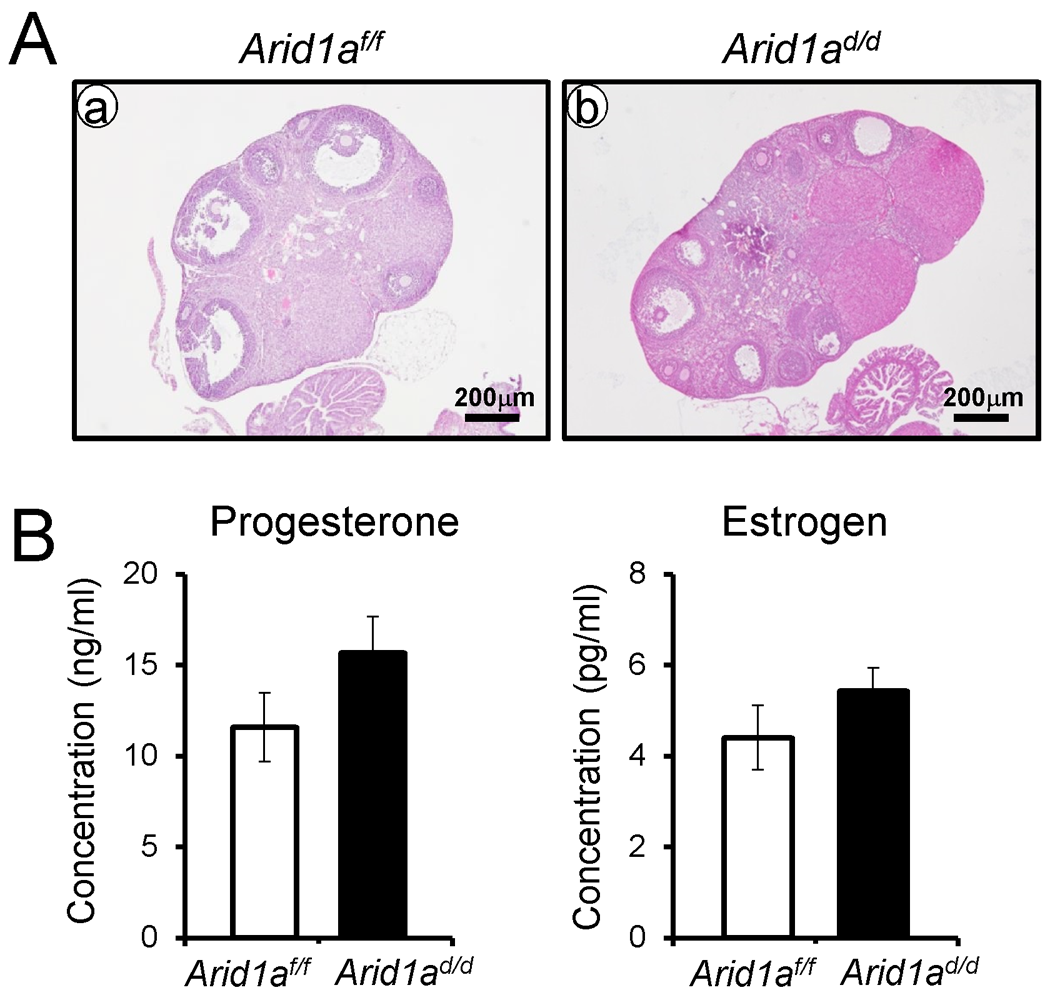

Supplement: S4 Fig — (A) Ovarian histology by H&E staining exhibited no difference between control (Arid1a f/f) (a) and Arid1a d/d mice (b). (B) The serum level of E2 and P4 were not different between control (Arid1a f/f) and Arid1a d/d mice at 3.5 dpc. The results represent the mean ± SEM. (TIF) [file pgen.1005537.s007.tif]

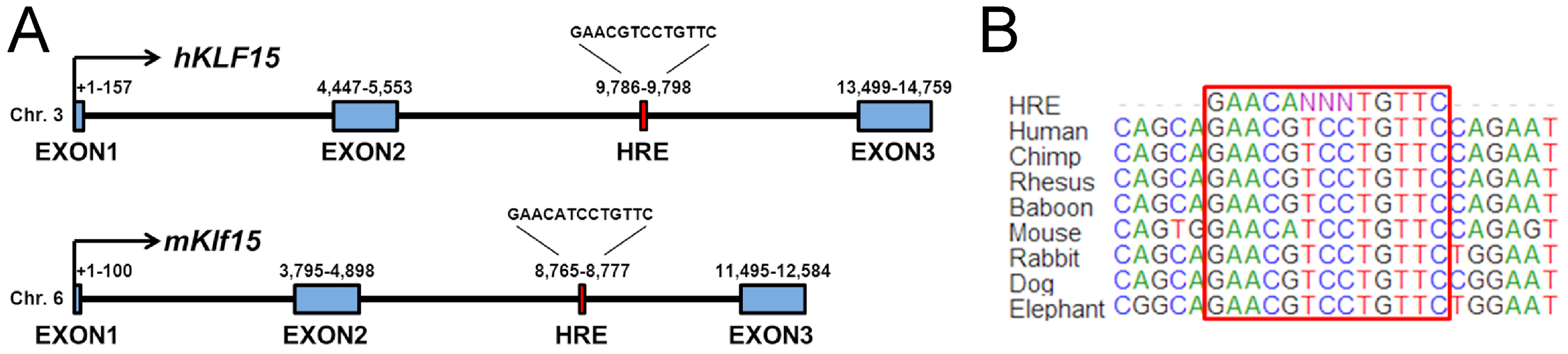

Supplement: S5 Fig — (TIF) [file pgen.1005537.s008.tif]
